# Transmission line foreign object segmentation based on RB-UNet algorithm

**DOI:** 10.7717/peerj-cs.2383

**Published:** 2024-10-10

**Authors:** Yan Wang, Qinghe Yuan, Ying Wang, Zhang Ruizhi, Qian Wu, Guoliang Feng

**Affiliations:** 1 Economic and Technological Research Institute, State Grid Heilongjiang Electric Power Co. Ltd., Harbin, Heilongjiang, China; 2 College of Information and Control Engineering, Jilin Institute of Chemical Technology, Jilin, Jilin, China; 3 School of Automation Engineering, Northeast Electric Power University, Jilin, Jilin, China

**Keywords:** Semantic segmentation, Mixture loss function, Transmission lines

## Abstract

**Background:**

The identification of foreign objects on transmission lines is crucial for their normal operation. There are risks and difficulties associated with identifying foreign objects on transmission lines due to their scattered distribution and elevated height.

**Methods:**

The dataset for this paper consists of search material from the web, including bird nests, kites, balloons, and rubbish, which are common foreign objects found on top of transmission lines, totaling 400 instances. To enhance the classical U-Net architecture, the coding component has been substituted with a ResNet50 network serving as the feature extraction module. In the decoding section, a batch normalization (BN) layer was added after each convolutional layer in the decoder to improve the model’s efficiency and generalization capacity. Additionally, a combined loss function was implemented, merging Focal loss and Dice loss, to tackle class imbalance issues and improve accuracy.

**Results:**

In summary, RB-UNet, a novel semantic segmentation network, has been introduced. The experimental results show a mIoU of 88.43%, highlighting the significant superiority of the RB-UNet approach compared to other semantic segmentation techniques for detecting foreign objects on transmission lines. The findings indicate that the proposed RB-UNet algorithm is proficient in detecting and segmenting foreign objects on transmission lines.

## Introduction

Transmission lines (Wang, Lin & Ma, 2024; Ji et al., 2023) as power channels are an important part of the power system, but they are exposed to outdoor environments for a long time (Zhao, 2024) and various accidents may occur, such as wildfires (Wei et al., 2023), component faults (Song et al., 2024), icing (Daolei et al., 2023), line shaking (An et al., 2024), and foreign object hanging (Liu et al., 2023). Among these, foreign object suspension is the most common. Common foreign object detections include bird nest detection (Dong et al., 2023), balloon detection (Yanzhen et al., 2022), kite detection (Wang et al., 2023), and rubbish detection (Shuang & Yu, 2022). To avoid interference from foreign objects on transmission lines (Sui et al., 2021; Hua et al., 2022), routine inspections are arranged. Inspection methods include manual inspection, drone inspection (Cailin et al., 2022; Wang, Shen & Zhou, 2024; Han et al., 2023), helicopter inspection (Du et al., 2019), and online video monitoring technology (Xiaohua et al., 2021; Gl, Luo & Gui, 2021; Laninga et al., 2023). Drone inspection and online video monitoring technology are the most commonly used methods; both of them apply image recognition technology to ensure the normal operation of the transmission lines (Dong et al., 2021).

With the recent development of deep learning technology in recent years (Khirwadkar & Deepa, 2024; Rahman et al., 2022), the technology for foreign object image recognition on transmission lines has also become more diverse. Nan et al. (2024) formed AS-Unet by introducing ASPP and SE modules into U-Net and then constructed the AS-Unet++ structure by using AS-Unet from different layers, effectively improving the recognition accuracy of transmission line images. According to this paper, it is known that the U-Net network as a base network is more advantageous for the task of semantic segmentation of transmission lines. Zhang et al. (2023) proposed a lightweight detection network, YOLOv5-IC-ST, which incorporated the improved versions of CSPDarknet and Swin Transformer into YOLOv5, reducing the influence of background factors on the model and improving the model’s feature extraction capabilities. Through this paper, it can be observed that the segmentation performance of the network can be increased by enhancing the feature extraction capability and thus improving the segmentation performance of the network. Jiang, Zhao & Wang (2023) improved the YOLOv7 model by introducing the dynamic convolution kernel ODconv and Alpha_GIoU loss function, which can effectively identify small targets and improve robustness. Through this paper, it can be observed that the recognition of small objects can be improved by employing different loss functions.

Although the aforementioned techniques have proven to be effective in recognizing foreign objects, they often lack accuracy in recognition and do not tackle the issue of class imbalance. Furthermore, U-Net networks are not efficient in handling complex image segmentation tasks, and the network convergence is slow. Additionally, obtaining transmission line images is challenging, and the types are frequently inconsistent. Therefore, this paper proposes an RB-UNet semantic segmentation model to achieve foreign object segmentation on transmission lines. The RB-UNet is an enhanced version of the classic U-Net network and contains the following key designs: (1) For U-Net semantic segmentation, the original network is primarily utilized in medical imaging. While it performs well with images having simple backgrounds, its segmentation effectiveness is limited with complex images due to insufficient feature extraction capabilities. To address this, an image classification network is incorporated to enhance feature extraction. The ResNet50 residual network is known for its strong feature extraction capabilities owing to its residual structure. Therefore, we opt for the U-Net network version of the ResNet50 network as the backbone network, utilizing ResNet50 as the feature extractor to enhance the model’s ability to extract image features. (2) The semantic segmentation task is more complex compared to image classification and target detection tasks. It often requires more resources, has longer training times, slower convergence, and involves a significant amount of image annotation tasks. To expedite model convergence, minimize resource and time wastage, alleviate task burden, and enhance stability, the addition of a batch normalization (BN) layer to the original network structure is proposed. This is achieved by incorporating a BN layer after each convolutional layer in the decoding block to enhance the convergence and generalization of the model. (3) For the category imbalance problem in the previous semantic segmentation task, variations in shooting angles, foreign object sizes, and other factors led to differences in the number of extractable pixel points for each image. Consequently, some categories had a significantly larger number of samples, while others had very few. This issue is unavoidable, prompting us to design and implement a unique loss function to address this challenge. Therefore, in order to improve model accuracy and address class imbalance issues, a mixture loss function that combines Dice loss and Focal loss has been introduced. This mixture loss function focuses the model’s attention on classes that have fewer pixel samples.

The institutional organisation of this paper consists of four parts, firstly the first part is the introduction, which describes the purpose and significance of the research as well as the methodology. The second part is the dataset and methodology, which describes the composition and production of the dataset, illustrates the improved methodology and network. The third part is the experimental part, which gives the experimental setup as well as the evaluation indexes, and lists the model experimental data as well as the corresponding ablation experiments and comparative experimental data. The fourth part is the conclusion, which summarises the work done in this paper, the final result data of the model, and the future directions.

## Materials & Methods

### The classical U-Net network model

**Figure 1:**
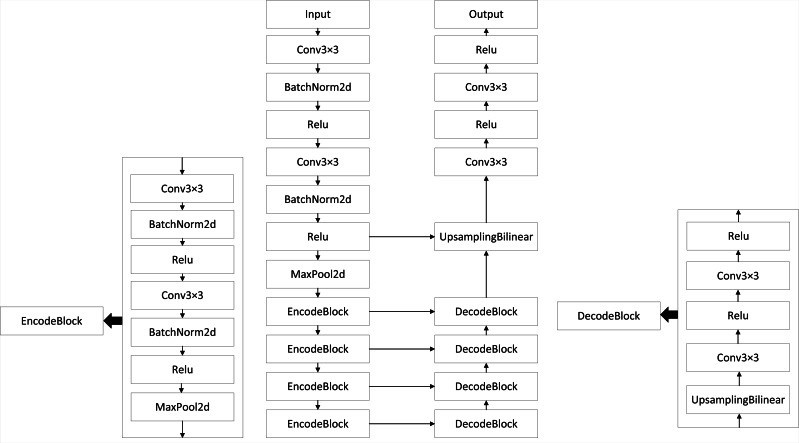
Architecture of the classical U-Net network.

Segmentation of foreign objects in transmission lines is a challenging task that requires an advanced semantic segmentation model. The U-Net network is selected as the foundational network for this segmentation task. The U-Net network consists of an encoder and a decoder. The encoder is responsible for extracting semantic features from the input images, while the decoder gradually upscales the low-resolution feature maps from the encoder back to the original image size. The encoder and decoder are connected through skip connections, allowing the decoder to access feature representations from the encoder and incorporate additional semantic information. The architecture of the U-Net network is illustrated in Fig. 1, showcasing the classical U-Net network architecture at the center, the EncodeBlock architecture on the left, and the DecodeBlock architecture on the right.

### ResNet50 network

In order to enhance the feature extraction capability of the U-Net network, it was necessary to replace the encoder section of the U-Net network with the ResNet50 network. ResNet50 is a specific type of residual network consisting of 50 convolutional neural network layers. It is characterized by residual blocks, global average pooling layers, and fully connected layers. The ResNet50 network is divided into five stages. The first stage, conv1, comprises a convolutional layer, a BN layer, and a ReLu layer. The convolutional layer uses a 7 × 7 kernel with a stride of 2. The subsequent four stages, conv2-x, conv3-x, conv4-x, and conv5-x, all contain residual blocks, with varying numbers of blocks in each stage. Specifically, there are 3, 4, 6, and 3 residual blocks in conv2-x, conv3-x, conv4-x, and conv5-x, respectively, totaling 16 residual blocks. The ResNet50 network is commonly employed for image classification tasks due to its exceptional feature extraction capability stemming from its residual structure.

### BN layer

In order to address the issue of slow convergence in the U-Net network, the properties of the BN layer are utilized. The BN layer is a technique that can expedite network convergence and enhance generalization capabilities. Training becomes more challenging with an excessively deep neural network, leading to significantly longer convergence times. Therefore, the BN layer is typically placed between the convolutional and activation layers to mitigate this issue. It standardizes each small batch of input data to have a mean close to 0 and a variance close to 1. The BN layer enhances both model stability and generalization capacity.

### RB-UNet network

**Figure 2:**
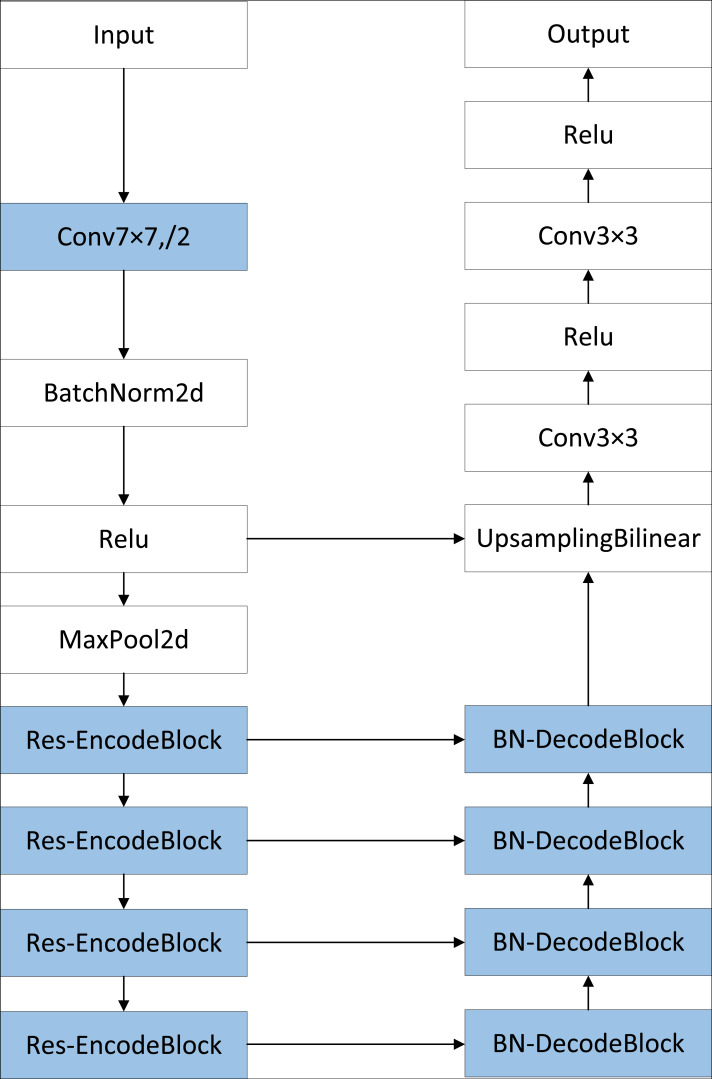
Structure of the RB-UNet.

The U-Net network was originally designed for the recognition and segmentation of medical images. However, it faced challenges when dealing with images with complex backgrounds due to limitations in feature extraction, category imbalance, and model overfitting. To address these issues and achieve better performance in recognizing foreign objects on power transmission lines with intricate backgrounds, a more sophisticated network is necessary. The proposed semantic segmentation model, RB-UNet, builds upon improvements to the original U-Net. Figure 2 illustrates the framework of RB-UNet, highlighting the enhanced and replaced sections in blue. In contrast to the original U-Net, the RB-UNet network utilizes ResNet50 as the feature extraction network for the encoder section, incorporating the residual structure to create the RB-UNet’s encoding block, Res-EncodeBlock. To enhance model stability, a BN layer is inserted between each convolutional layer and the activation layer in the DecodeBlock of the decoder section, resulting in BN-DecodeBlock. Additionally, to address the class imbalance problem, a hybrid loss function combining Dice loss and Focal loss is introduced.

### Res-EncodeBlock

**Figure 3:**
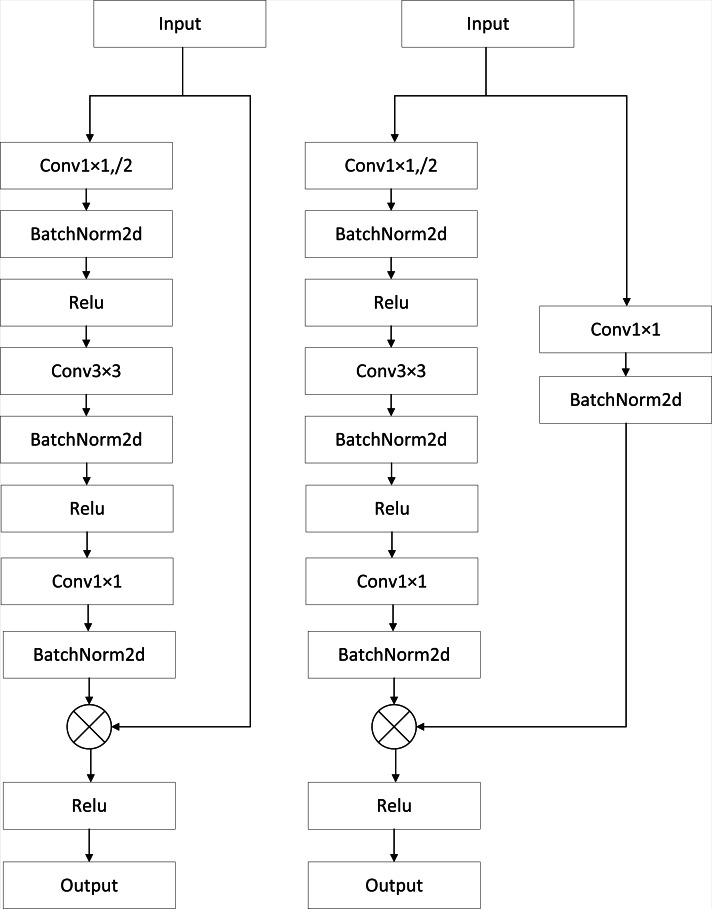
ResNet50 residual block.

ResNet50 is introduced as the U-Net network’s encoder to improve the extraction of semantic features from images, lessen reliance on a large amount of labeled data, and enhance model performance and generalization ability. This addresses the issue of the classic U-Net network’s weak feature extraction capability. The ResNet50 network is composed of five stages: conv1, conv2-x, conv3-x, conv4-x, and conv5-x. As illustrated in Fig. 2, the first two convolutional layers of the original U-Net network are first replaced by the convolutional layers in conv1. The U-Net network’s EncodeBlock is then replaced with each of the final four stages of the ResNet50 network, creating the encoding block Res-EncodeBlock. Each Res-EncodeBlock contains a different number of ResNet50 residual blocks; the number of residual blocks are 3, 4, 6, and 3 in that order, to be used for better extraction of image feature information. Figure 3 displays the ResNet50 residual blocks’ structure. The residual block on the left is made up of three convolutional layers: a 1 × 1 convolutional layer for dimensionality reduction, a 3 × 3 convolutional layer for feature extraction, and a 1 × 1 convolutional layer for dimensionality increase, as well as the corresponding BN layer, ReLu layer, and skip connection. An extra 1x1 convolutional layer and BN layer are included in the residual block on the right, above the skip connection. They are used to modify the feature map dimensions in cases where the input and output dimensions do not match.

### BN-DecodeBlock

**Figure 4:**
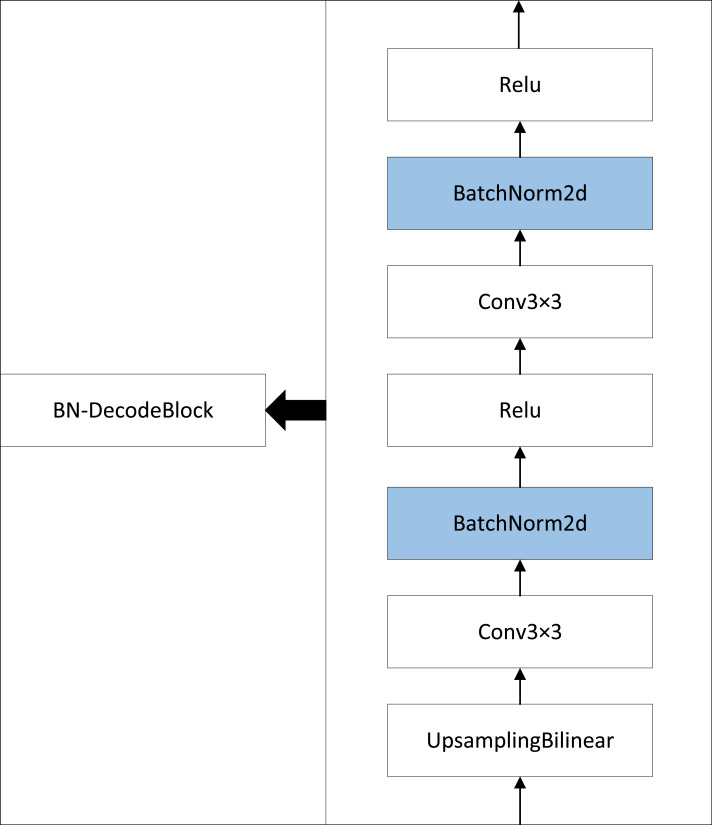
BN-DecodeBlock architecture composition.

To address the limitations of the classic U-Net network in terms of stability and generalization capacity, this paper introduces the BN layer to the original U-Net model. As illustrated in Fig. 4, incorporating a BN layer after the convolutional layers in the DecodeBlock normalizes the outputs of the convolutional layers. This transformation results in a standard normal distribution with a mean of 0 and variance of 1. This approach can help the model to increase stability and converge quickly.

### The mixed loss function

One of the loss functions frequently used in semantic segmentation is CE loss. The objective of this loss function is to minimize the difference between the predicted and true labels.

(1) \begin{eqnarray*}{L}_{CE \left( p,y \right) }=-\sum _{i}{y}_{i}\log \nolimits \left( {p}_{i} \right) \end{eqnarray*}

where *L*_*CE*_ represents the cross-entropy loss value, *y*_*i*_ denotes the true label value of class *i*, and *p*_*i*_ represents the predicted label value of class *i*.

When used in semantic segmentation tasks, the Dice loss function is frequently employed to evaluate model performance by quantifying the overlap between true and predicted labels. By effectively handling class imbalance in semantic segmentation, the Dice loss function enhances model Precision and mIoU.

(2) \begin{eqnarray*}{L}_{D}=1- \frac{2\times TP}{2\times TP+FP+FN} \end{eqnarray*}

To enhance accuracy and address class imbalance, the Focal loss function assigns greater weight to harder-to-classify and less frequent categories. This approach assists the model in managing the distribution imbalance between the numerous background categories and the limited target categories in semantic segmentation tasks, thereby enhancing the model’s learning capacity.

(3) \begin{eqnarray*}{L}_{F} \left( {p}_{t} \right) =-{\alpha }_{t} \left( 1-{p}_{t} \right) \gamma \log \nolimits \left( {p}_{t} \right) \end{eqnarray*}

where *p*_*t*_ represents the probability of predicting this category, *α*_*t*_ denotes the weight of this, and *γ* represents the exponential parameter.

To tackle the issue of class imbalance, one approach is to address it through the loss function. CE loss primarily emphasizes pixel-level classification accuracy, but its effectiveness diminishes on imbalanced datasets and small targets. In contrast, Dice loss evaluates the level of overlap between the predicted outcomes and the actual labels, resulting in superior performance with balanced datasets. Focal loss excels in handling class imbalance by assigning greater weights to challenging samples, thereby mitigating the issue of low accuracy for scarce samples. Therefore, combining Dice loss and Focal loss into a composite loss function can better solve the category imbalance problem.

(4) \begin{eqnarray*}{L}_{mix}={L}_{D}+{L}_{F}.\end{eqnarray*}

### Evaluation metrics

Based on the confusion matrix, the metrics mIoU, Recall, F1-score, Precision, and PA are frequently used in semantic segmentation. These five indicators allow for a comprehensive evaluation of the accuracy and performance of the semantic segmentation model. They provide insights into various aspects of the model’s actual performance.

(1) mIoU: Indicates the degree of overlap between the predicted segmentation results and the true segmentation results by calculation.

(5) \begin{eqnarray*}mIoU= \frac{1}{k+1} \sum _{i=0}^{k} \frac{TP}{TP+FP+FN} \end{eqnarray*}

(2) Recall: It shows the percentage of real positive samples that were accurately predicted to be positive out of true positive samples.

(6) \begin{eqnarray*}\text{Recall}= \frac{TP}{TP+FN} \end{eqnarray*}

(3) F1-score: It represents the harmonic mean of Recall and Precision.

(7) \begin{eqnarray*}F1socre=2\times \frac{\text{Recall}\times \text{Precision}}{\text{Recall}+\text{Precision}} \end{eqnarray*}

(4) Precision: It shows the likelihood that samples that are expected to be positive will, in fact, be positive.

(8) \begin{eqnarray*}\text{Precision}= \frac{TP}{TP+FP} \end{eqnarray*}

(5) PA: It represents the percentage of samples that were correctly classified out of all the samples.

(9) \begin{eqnarray*}PA= \frac{TP+TN}{TP+TN+FP+FN} \end{eqnarray*}

### Dataset

Due to the unavailability of a publicly accessible dataset of foreign objects on transmission lines for training purposes, this study collected images of foreign object interference on transmission lines from the internet to create a dataset. Although the foreign object dataset in this paper is collected from the internet, the images in the dataset are real-world images, so there is no need to worry about the disconnect between the internet and the real world. This dataset includes four categories: bird nests, balloons, kites, and rubbish. These images vary in the distance and size of the target object. A total of 400 photos were chosen, with 100 for each category, as illustrated in Table 1. From these images, 80 were selected for the training set and 20 for the test set. Using labeling tools like Labelme, the dataset was tagged at the pixel level, resulting in 400 pixel-level annotated images—100 for each category. Unlike image classification and object detection tasks, semantic segmentation models focus more on the number of pixels. The number of object pixels supplied to the model varies for each image in the foreign object dataset on transmission lines due to variables such as the size and distance at which the objects were photographed. This implies that the issues of small sample numbers and class imbalance need to be addressed in the semantic segmentation model discussed in this paper. Although the dataset is relatively small and encounters the issue of class imbalance, it still presents various scenarios that can assist in training the model to effectively identify and segment foreign objects in different situations.

**Table 1:** Sample dataset division.

Dataset	Bird’s nest	Balloon	Kite	Rubbish	Total
Training set	80	80	80	80	320
Test set	20	20	20	20	80

### Training models and experimental settings

The training hardware utilized an NVIDIA GeForce RTX 4090 GPU for training. The dataset was divided into training and test sets using an 8:2 ratio. Throughout the experiments, the input image resolution was standardized to 512 × 512. The training process employed an Adam optimizer with a batch size of 24 and lasted for 100 epochs. The minimum learning rate was set to be equal to the maximum learning rate multiplied by 0.01, with the maximum learning rate set to 1e−4.

## Results

Accuracy metrics were obtained by training the RB-UNet network under the experimental parameters, as outlined in Table 2. The accuracy measurements for the background are the highest due to its larger pixel sample count. In contrast, the accuracy metrics for bird nests are the lowest. Bird nests present challenges due to their complex appearances, irregular shapes, and frequent obstruction by towers, making it difficult to discern the full appearance or divide nests into distinct parts, thus increasing the segmentation difficulty. Similarly, the accuracy metrics for the rubbish category are also poor because most rubbish consists of small, irregularly shaped pieces, posing challenges for recognition. Kites and balloons, on the other hand, have regular shapes and occupy a significant portion of the photos, resulting in relatively good accuracy metrics compared to the other two categories.

**Table 2:** Accuracy metrics.

Accuracy metrics	Background	Bird’s nest	Kite	Balloon	Rubbish	Average %
mIoU	0.96	0.77	0.93	0.95	0.81	88.43
Precision	0.99	0.83	0.96	0.97	0.89	92.72
F1-score	0.98	0.87	0.96	0.97	0.90	93.69
Recall	0.97	0.93	0.96	0.98	0.91	94.77
PA	–	–	–	–	–	96.43

## Discussion

**Figure 5:**
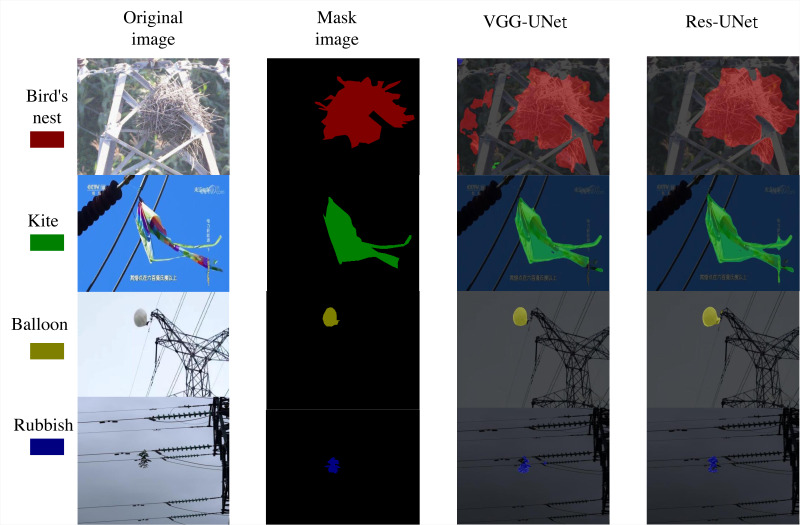
Predicted results comparison. Image source credit: https://aistudio.baidu.com/datasetdetail/212110, CC BY-NC-SA 4.0.

The enhanced U-Net architecture typically involves replacing the encoder section to improve feature extraction capabilities. In most cases, the VGG16 and ResNet50 networks are selected as alternatives for this component. Consequently, an experiment was conducted to compare the performance of the two backbone networks using the VGG-UNet and Res-UNet models. The VGG-UNet model entails substituting the encoder part of the U-Net with the VGG-16 network architecture. The Res-UNet model replaces the encoder part of the original U-Net model with the ResNet50 network. As illustrated in Table 3, the comparative analysis of experimental data shows that the evaluation metrics for the Res-UNet architecture generally outperform those of the VGG-UNet architecture. As shown in Fig. 5, the segmentation effect graphs of the two models are compared, and it can be seen that the segmentation effect of Res-UNet is better. Primarily showcasing residual network characteristics, the Res-UNet structure ensures effective feature learning by addressing the vanishing gradient issue efficiently during network training. While the VGG-UNet structure also leverages a robust feature extractor in VGG16, its performance in complex scenarios is limited by its relatively simple structure. In summary, opting for ResNet50 as the feature extraction component of the U-Net network is more appropriate for the task of segmenting foreign objects in transmission lines and yields superior segmentation outcomes.

**Table 3:** Comparative experiment of backbone networks.

Model	mIoU	Precision	F1-score	Recall	PA
Res-UNet	82.79	86.47	90.21	94.70	94.85
VGG-UNet	81.95	85.58	89.61	94.74	94.82

**Figure 6:**
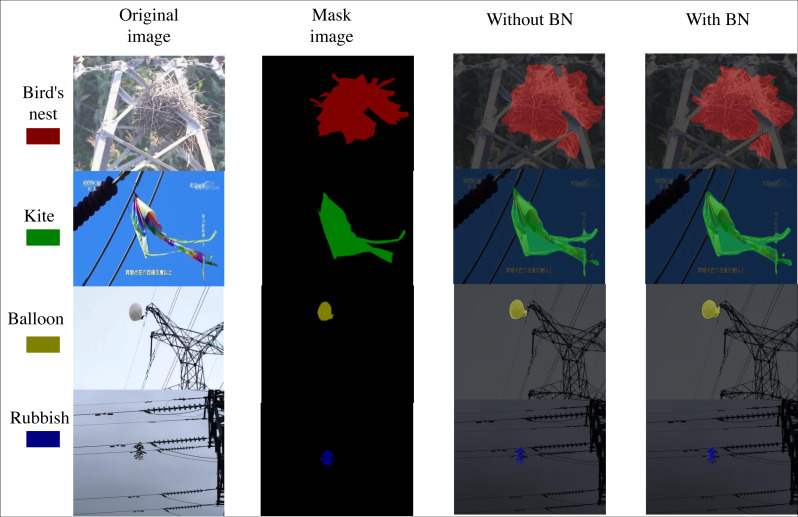
Comparison of the segmentation effect in the BN layer ablation experiment. Image source credit: https://aistudio.baidu.com/datasetdetail/212110, CC BY-NC-SA 4.0.

In order to verify whether adding a BN layer to the decoding block improves model stability and speeds up convergence, experiments were conducted for both scenarios, as shown in Table 4. Evaluation measures showed that the model with the additional BN layer in the decoding block performed better than the model without the BN layer: improvements of 2.88% in mIoU, 2.66% in Precision, 1.82% in F1-score, 0.67% in Recall, and 0.85% in PA. The specific segmentation effect is shown in Fig. 6. In conclusion, incorporating a BN layer into the decoding block can boost the learning and generalization abilities of the model, enhance performance, improve model stability, decrease dependence on initialization, and offer a form of regularization.

**Table 4:** The comparative experiment of with and without a BN layer.

Model	mIoU	Precision	F1-score	Recall	PA
Without BN layer	82.79	86.47	90.21	94.70	94.85
With BN layer	85.67	89.13	92.03	95.37	95.70

**Figure 7:**
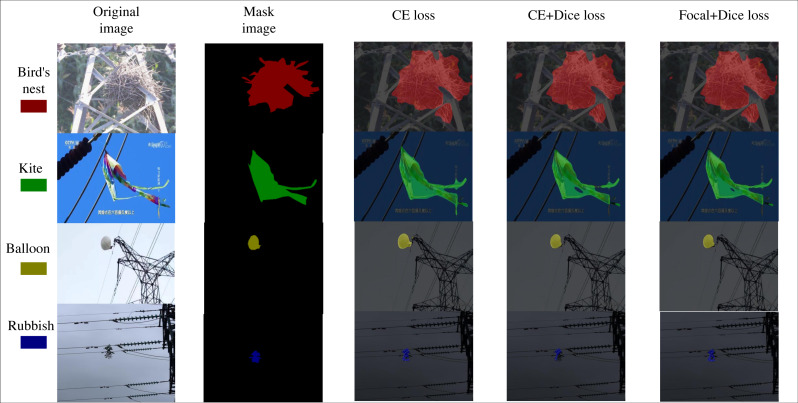
Comparison of segmentation effects of loss function ablation experiments. Image source credit: https://aistudio.baidu.com/datasetdetail/212110, CC BY-NC-SA 4.0.

Experiments were conducted to compare CE loss, CE loss + Dice loss and Dice loss + Focal loss in order to analyse the impact of the three loss functions on the model performance, to determine whether they are effective in solving the category imbalance problem and to identify the best loss function. The specific segmentation effect is shown in Fig. 7. The goal of these experiments was to verify whether the combination of Dice loss and Focal loss in the mixed loss function improves model performance. Table 5 illustrates that there was some performance improvement in the mixed loss function (Dice loss + Focal loss) compared to CE loss. All evaluation indicators showed improvements when compared to CE loss and Dice loss. Among several combinations of loss functions, it is evident that Dice loss +Focal loss is more effective in addressing class imbalance issues. This combination directs the model’s focus towards categories with limited sample sizes. By mitigating class imbalance, the model’s performance improves, leading to an enhancement in the mIoU value. Overall, it can be concluded that the mixed loss function (Dice loss + Focal loss) provides a slight overall gain in model segmentation capabilities. The experimental data demonstrates that the mixed loss function (Dice loss + Focal loss) is more suitable for the segmentation task in this study.

**Table 5:** Comparative experiment of loss functions.

Loss function	mIoU	Precision	F1-score	Recall	PA
CE loss	82.79	86.47	90.21	94.70	94.85
CE+Dice loss	85.43	90.51	91.84	93.40	95.58
Focal+Dice loss	86.09	91.24	92.26	93.45	95.87

In order to assess the performance of the RB-UNet model and the feasibility of improvements, we compared its performance with four mainstream semantic segmentation models: Res-PSPNet, MobileNetv2-PSPNet, Xception-Deeplabv3+Net, and MobileNetv2-Deeplabv3+Net. These versions perform better than the ordinary models because they have been optimized using their original network structure. By contrasting the RB-UNet model with these four models, the progress and utility of the model can be better understood, showcasing the complete benefits and advancements of the RB-UNet model. In Res-PSPNet and MobileNetv2-PSPNet, ResNet50 and MobileNetv2 are used as encoders for the PSPNet model, respectively. In Xception-Deeplabv3+Net and Mobilenetv2-Deeplabv3+Net, Xception and MobileNetv2 were used as encoders for the Deeplabv3+Net model, respectively. As shown in Table 6, RB-UNet scores 88.43%, 92.72%, 93.69%, 94.77%, and 96.43% on mIoU, Precision, F1-score, Recall, and PA metrics, respectively, which is better than the other four models.

**Table 6:** Comparative experiment of mainstream networks.

Model	mIoU	Precision	F1-score	Recall	PA
Mobilenetv2-PSPNet	49.68	75.38	56.70	52.58	93.54
Xception-Deeplabv3+Net	66.14	93.38	72.97	68.89	95.91
Res-PSPNet	80.42	90.05	88.76	87.57	95.42
Mobilenetv2-Deeplabv3+Net	86.12	93.56	92.35	91.23	96.75
RB-UNet	88.43	92.72	93.69	94.77	96.43

When compared with the MobileNetv2-Deeplabv3+Net model, the RB-UNet model showed higher mIoU, F1-score, and recall, albeit with a slight decrease in precision and PA. Nevertheless, the RB-UNet model is generally better than the MobileNetv2-Deeplabv3+Net since mIoU offers a more thorough evaluation of the model.

It is evident that the proposed model surpasses MobileNetv2-PSPNet and Xception-Deeplabv3+Net. However, the experimental results of MobileNetv2-PSPNet and Xception-Deeplabv3+Net are notably lower. This suggests that the feature extraction network’s insufficient capability and slow convergence speed lead to poor learning outcomes. This underscores the significance of the feature extraction network in the overall network performance. Furthermore, comparing MobileNetv2-PSPNet with Res-PSPNet reinforces this perspective. The performance variation within the same PSPNet network emphasizes the critical role of the feature extraction network selection and underscores the superiority of ResNet50. Collectively, this clearly illustrates the advantage of opting for ResNet50 as the feature extraction network to enhance feature extraction capabilities in this study.

The experimental values of the two enhanced Deeplabv3+Net models are inferior to the model in this study due to the slow convergence rate during training. The main reason is that the BN layer structure is added to the model in this paper to speed up the convergence. Considering the limitation of the same limited training resources and datasets, the experimental results of the two enhanced Deeplabv3+Net models are lower than the performance achieved in this study, which demonstrates the effectiveness of adding BN layers to the proposed model.

**Figure 8:**
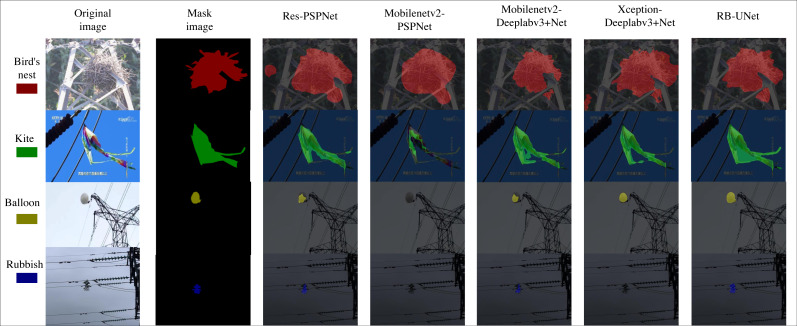
Predicted results comparison. Image source credit: https://aistudio.baidu.com/datasetdetail/212110, CC BY-NC-SA 4.0.

As illustrated in Fig. 8, the predicted results from RB-UNet and the other four models are compared. Different colors are assigned to bird nests, kites, balloons, and rubbish, represented by red, green, yellow, and blue, respectively. The corresponding color is used to indicate the presence of the foreign object in the image, specifying its type, shape, size, and position in the generated results. The graphic clearly shows that the RB-UNet model provides more accurate and intricate foreign object segmentation. The segmentation results of the other four models are all slightly worse. Res-PSPNet and MobileNetv2-DeepLabv3+Net, which have better segmentation results, can correctly identify foreign objects, but the segmentation results lack detail. On the other hand, the two networks MobileNetv2-PSPNet and Xception-DeepLabv3+Net, which have poor segmentation results, fail to identify foreign objects in the segmented image, and their performance is very low. This indicates that the RB-UNet model outperforms the other models in terms of performance. Furthermore, there is noticeable enhancement in addressing the category imbalance issue, accelerating convergence, and achieving a more refined segmentation effect. In summary, the proposed RB-UNet model exhibits superior performance in detecting and segmenting foreign objects on power transmission lines.

## Conclusions

The transmission line is an essential component of the power system for transmitting and distributing electrical energy. The identification and segmentation of foreign objects on power transmission lines can reduce human and material waste. It also provides real-time information about the type, size, and shape of foreign objects, enabling appropriate measures to be taken for resolution. Therefore, it is crucial for the regular operation and maintenance of electricity transmission lines to successfully identify and segment foreign objects on those lines. This paper proposes the RB-UNet semantic segmentation network. To enhance the model’s ability to extract image features and obtain more feature information, the ResNet50 network was chosen for the coding part of the segmentation network. Network convergence is expedited by adding a BN layer to the DecodeBlock, which improves the model’s stability and performance. Additionally, to tackle the issue of foreign objects causing class imbalance on power transmission lines, a mixed loss function that combines Dice loss and Focal loss is employed. With an mIoU of 88.43%, Precision of 92.72%, F1-score of 93.69%, Recall of 94.77%, and PA of 96.43%, the RB-UNet semantic segmentation model effectively segments and recognizes foreign objects on power transmission lines. This paper has made significant improvements in addressing the issue of class imbalance and accelerating convergence. However, the segmentation effect on complex images is not very precise, and the edge processing is relatively coarse. Additionally, due to the large size of the semantic segmentation model, it requires more resources. Future research should focus on refining the edge processing for complex image segmentation and reducing the model size.

##  Supplemental Information

10.7717/peerj-cs.2383/supp-1 Data S1 Data

10.7717/peerj-cs.2383/supp-2 Supplemental Information 2 Code

## Additional Information and Declarations

### Data Availability

The following information was supplied regarding data availability: The raw data and codes are available in the Supplementary Files. The Transmission Channel Hidden Danger Detection-Preliminary Dataset is available at: https://aistudio.baidu.com/datasetdetail/212110.
