# Supplementary figures and images for: Transmission line foreign object segmentation based on RB-UNet algorithm (part 2 of 2)
Source: PeerJ Comput Sci. 2024 Oct 10;10:e2383. doi: 10.7717/peerj-cs.2383 (PMC11622974; doi:10.7717/peerj-cs.2383)

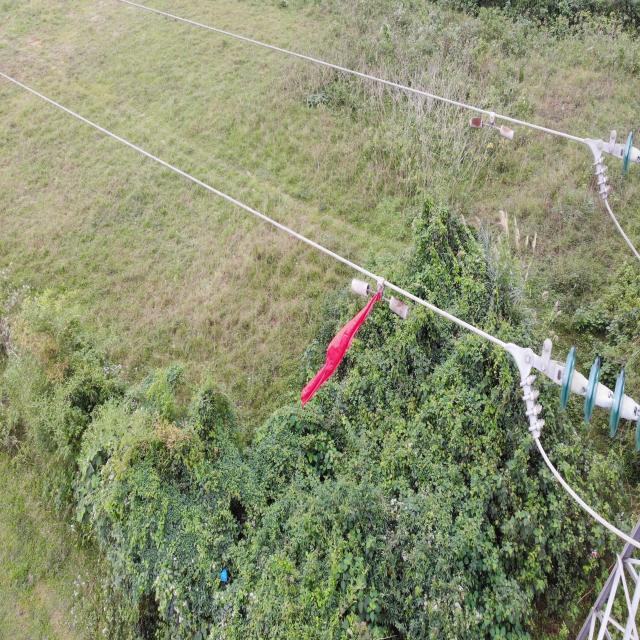

Supplement: Data S1 [file peerj-cs-10-2383-s001.zip › JPEGImages/DJI_0017_JPG.jpg]

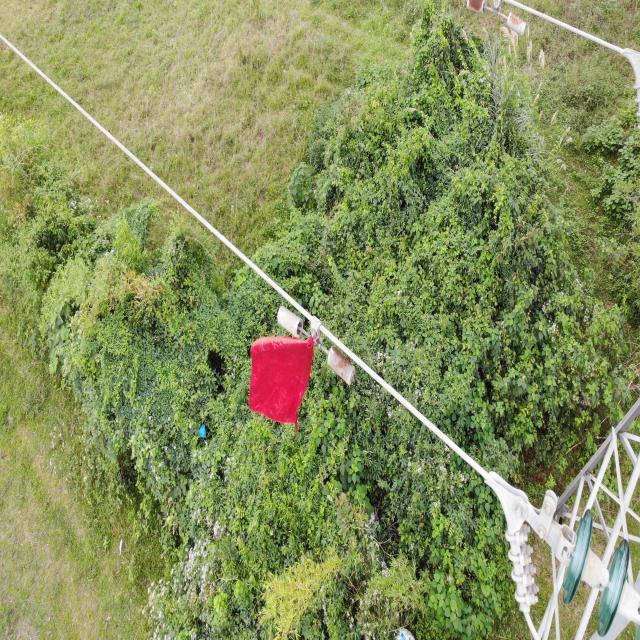

Supplement: Data S1 [file peerj-cs-10-2383-s001.zip › JPEGImages/DJI_0019_JPG.jpg]

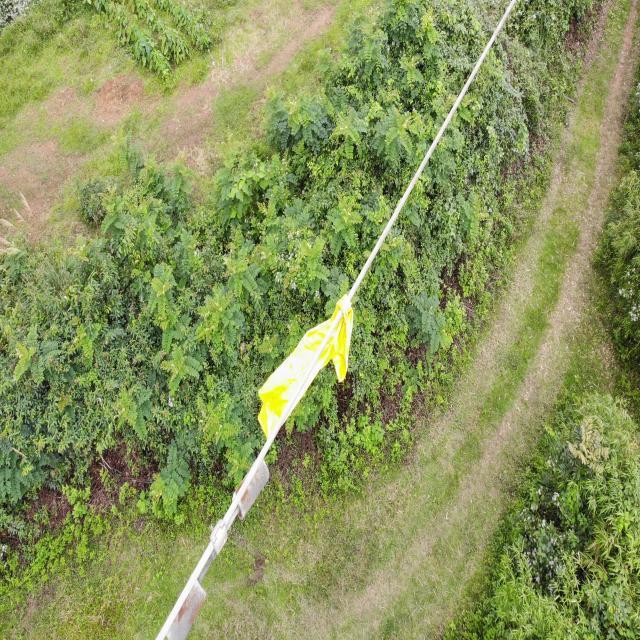

Supplement: Data S1 [file peerj-cs-10-2383-s001.zip › JPEGImages/DJI_0021_JPG.jpg]

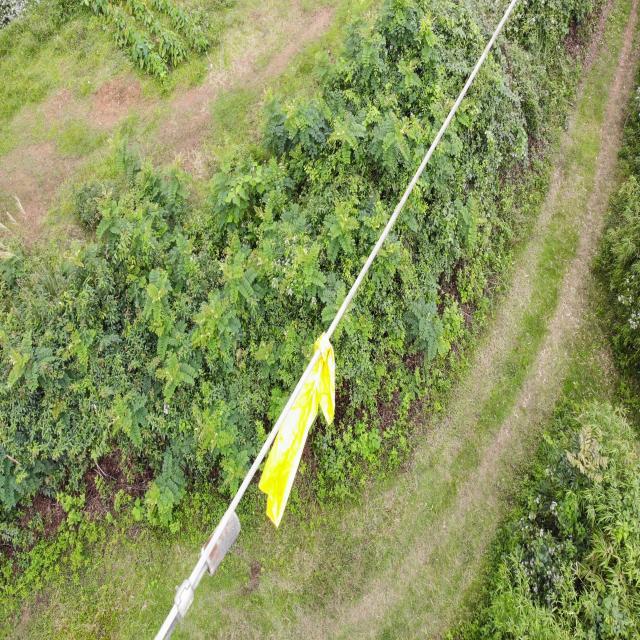

Supplement: Data S1 [file peerj-cs-10-2383-s001.zip › JPEGImages/DJI_0023_JPG.jpg]

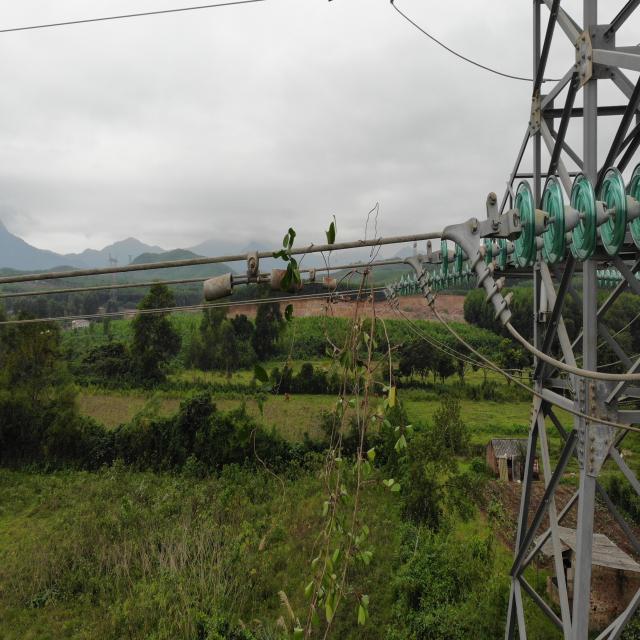

Supplement: Data S1 [file peerj-cs-10-2383-s001.zip › JPEGImages/DJI_0900_JPG.jpg]

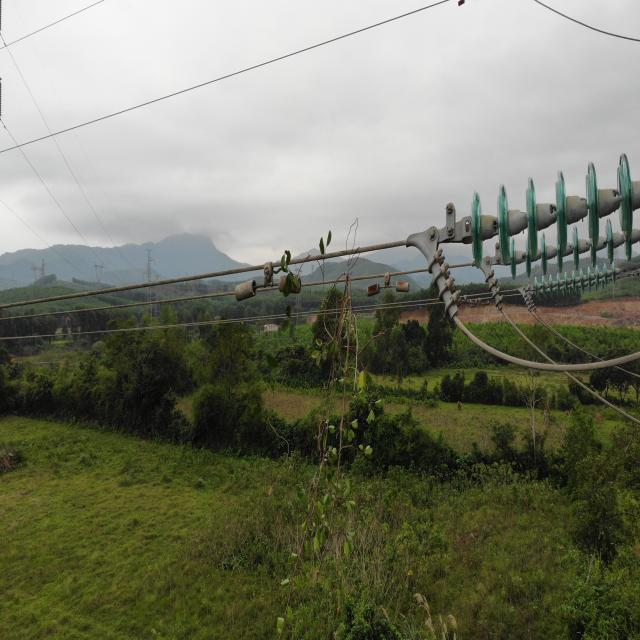

Supplement: Data S1 [file peerj-cs-10-2383-s001.zip › JPEGImages/DJI_0902_JPG.jpg]

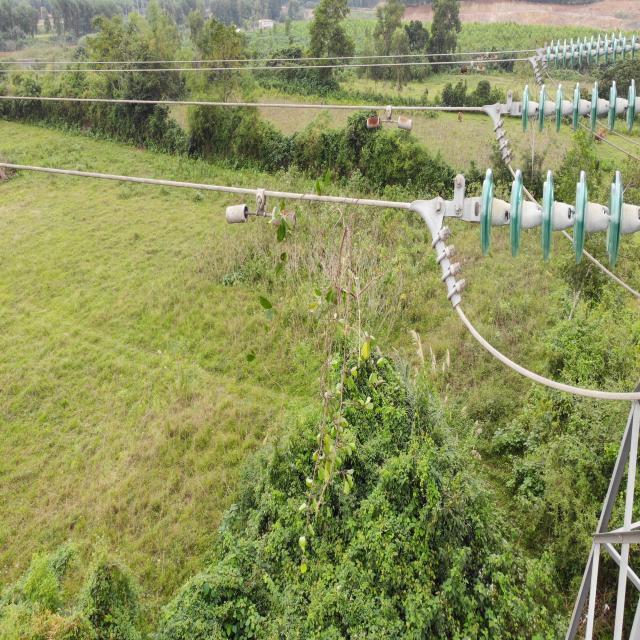

Supplement: Data S1 [file peerj-cs-10-2383-s001.zip › JPEGImages/DJI_0904_JPG.jpg]

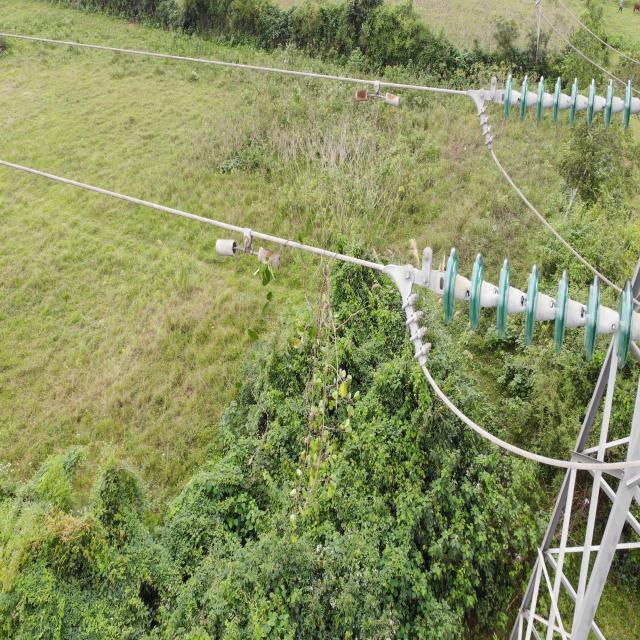

Supplement: Data S1 [file peerj-cs-10-2383-s001.zip › JPEGImages/DJI_0906_JPG.jpg]

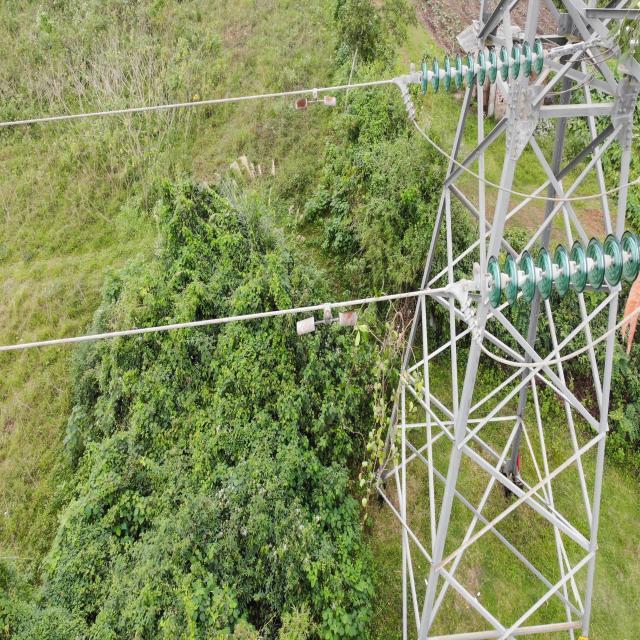

Supplement: Data S1 [file peerj-cs-10-2383-s001.zip › JPEGImages/DJI_0908_JPG.jpg]

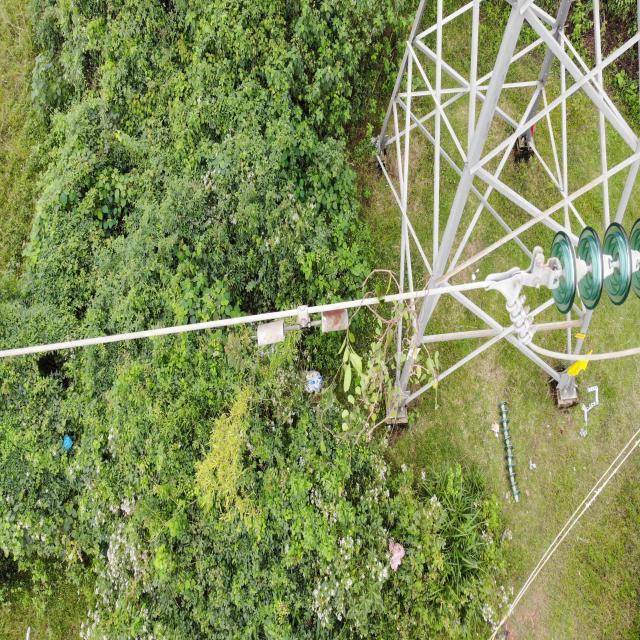

Supplement: Data S1 [file peerj-cs-10-2383-s001.zip › JPEGImages/DJI_0910_JPG.jpg]

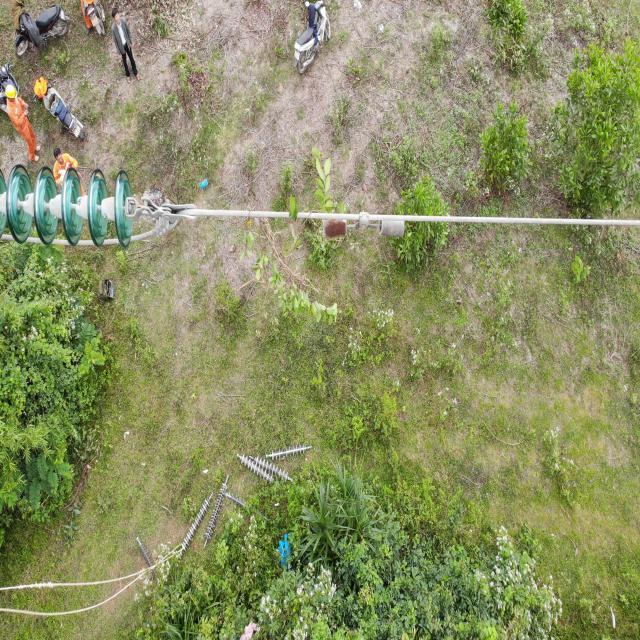

Supplement: Data S1 [file peerj-cs-10-2383-s001.zip › JPEGImages/DJI_0912_JPG.jpg]

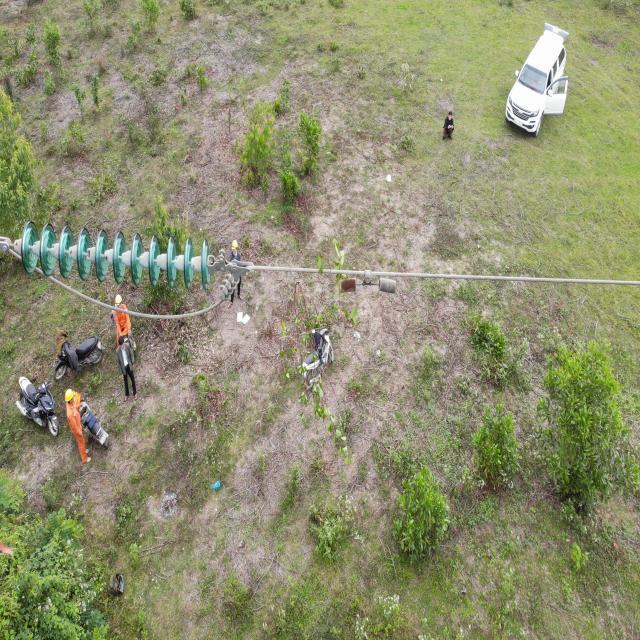

Supplement: Data S1 [file peerj-cs-10-2383-s001.zip › JPEGImages/DJI_0914_JPG.jpg]

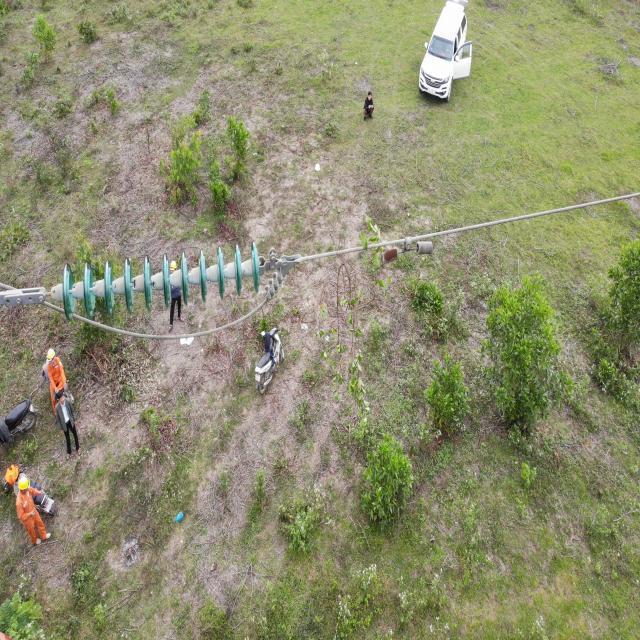

Supplement: Data S1 [file peerj-cs-10-2383-s001.zip › JPEGImages/DJI_0916_JPG.jpg]

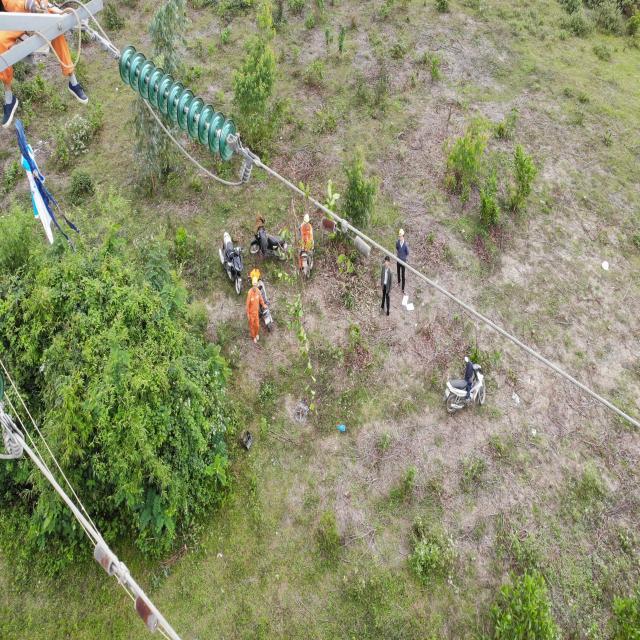

Supplement: Data S1 [file peerj-cs-10-2383-s001.zip › JPEGImages/DJI_0918_JPG.jpg]

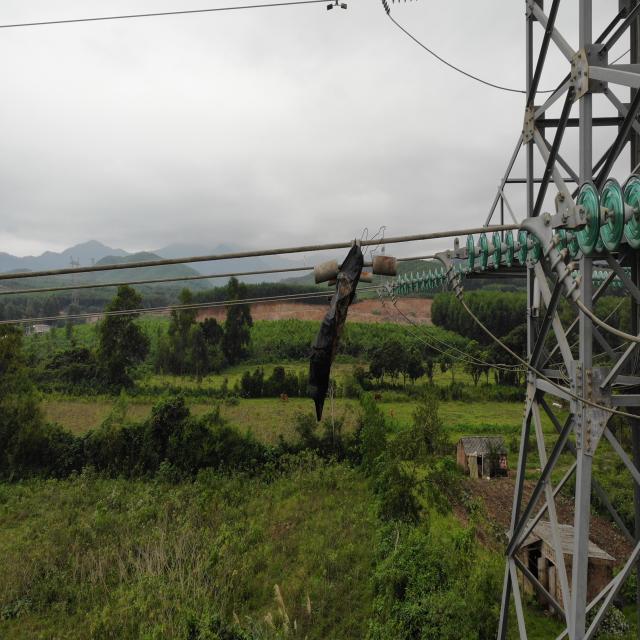

Supplement: Data S1 [file peerj-cs-10-2383-s001.zip › JPEGImages/DJI_0920_JPG.jpg]

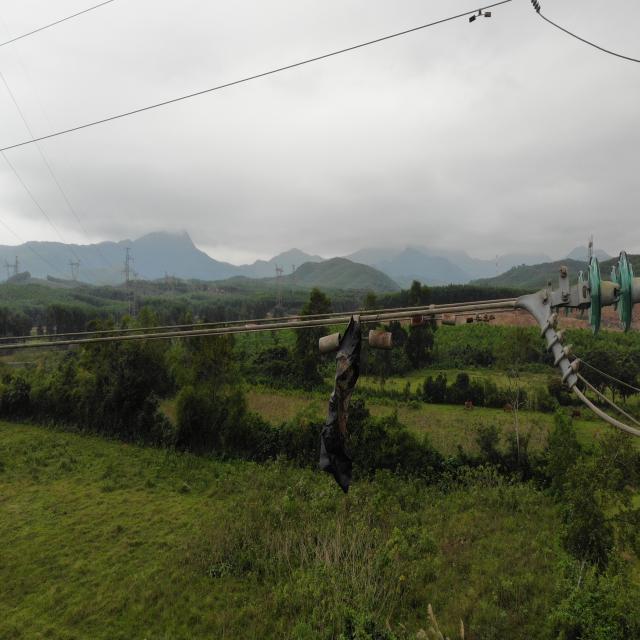

Supplement: Data S1 [file peerj-cs-10-2383-s001.zip › JPEGImages/DJI_0922_JPG.jpg]

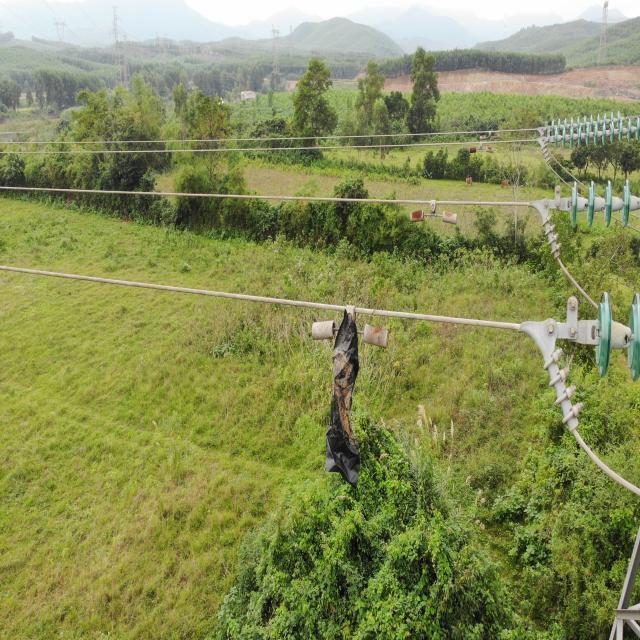

Supplement: Data S1 [file peerj-cs-10-2383-s001.zip › JPEGImages/DJI_0924_JPG.jpg]

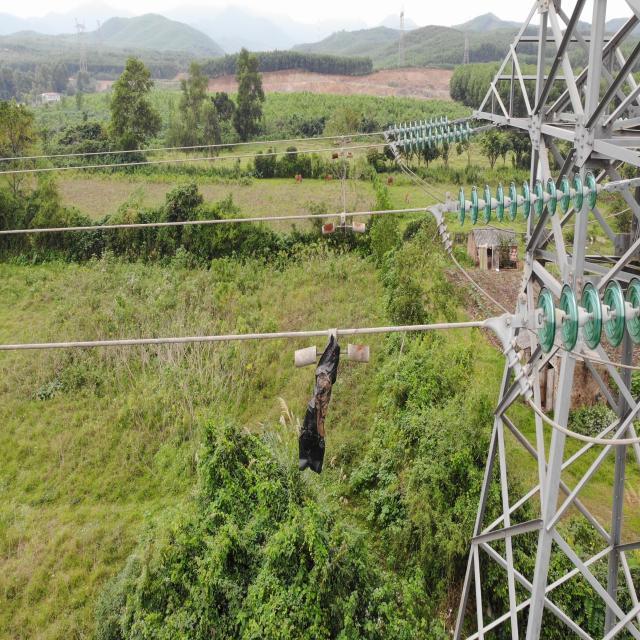

Supplement: Data S1 [file peerj-cs-10-2383-s001.zip › JPEGImages/DJI_0926_JPG.jpg]

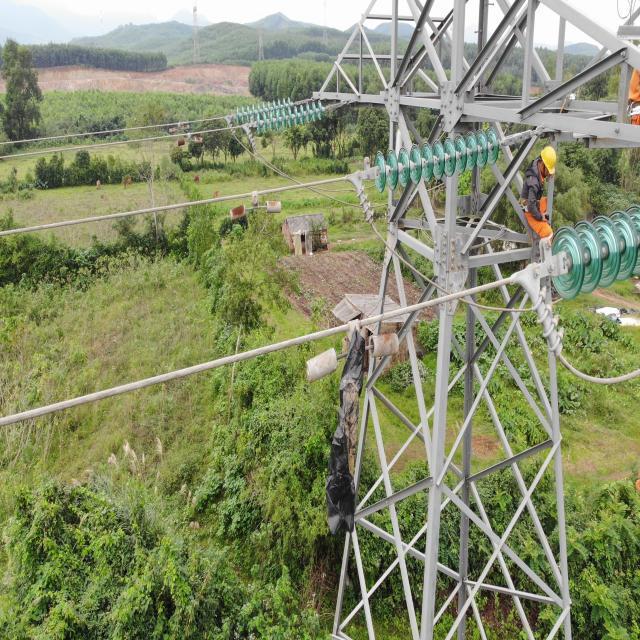

Supplement: Data S1 [file peerj-cs-10-2383-s001.zip › JPEGImages/DJI_0928_JPG.jpg]

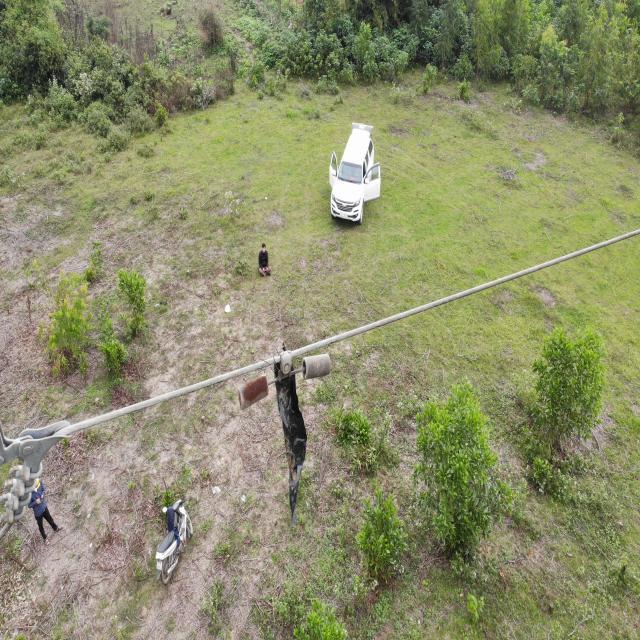

Supplement: Data S1 [file peerj-cs-10-2383-s001.zip › JPEGImages/DJI_0930_JPG.jpg]

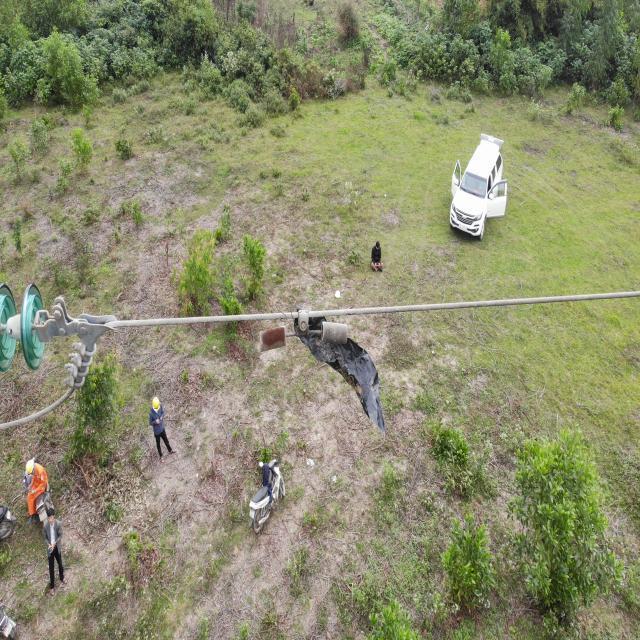

Supplement: Data S1 [file peerj-cs-10-2383-s001.zip › JPEGImages/DJI_0932_JPG.jpg]

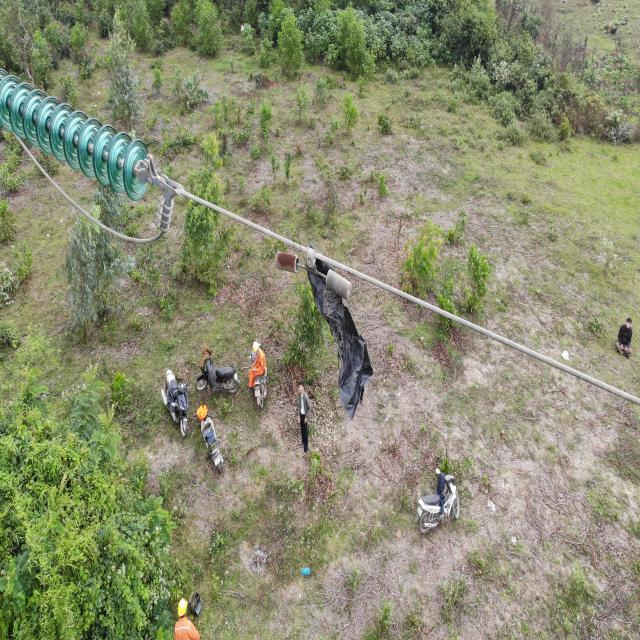

Supplement: Data S1 [file peerj-cs-10-2383-s001.zip › JPEGImages/DJI_0934_JPG.jpg]

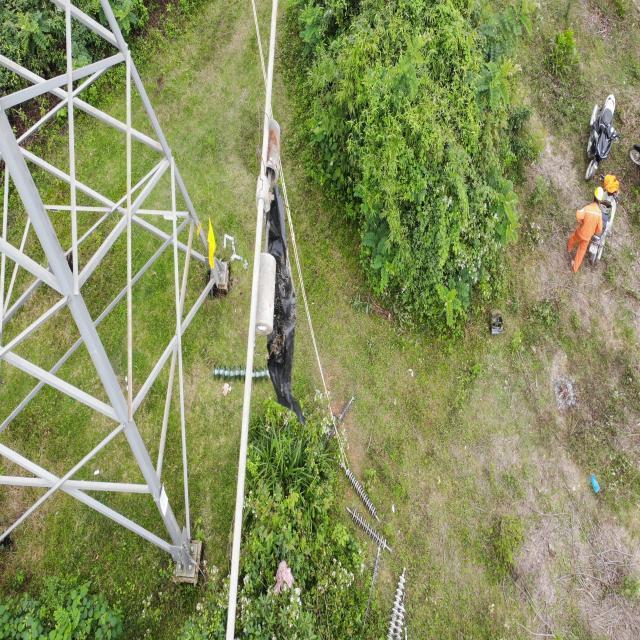

Supplement: Data S1 [file peerj-cs-10-2383-s001.zip › JPEGImages/DJI_0936_JPG.jpg]

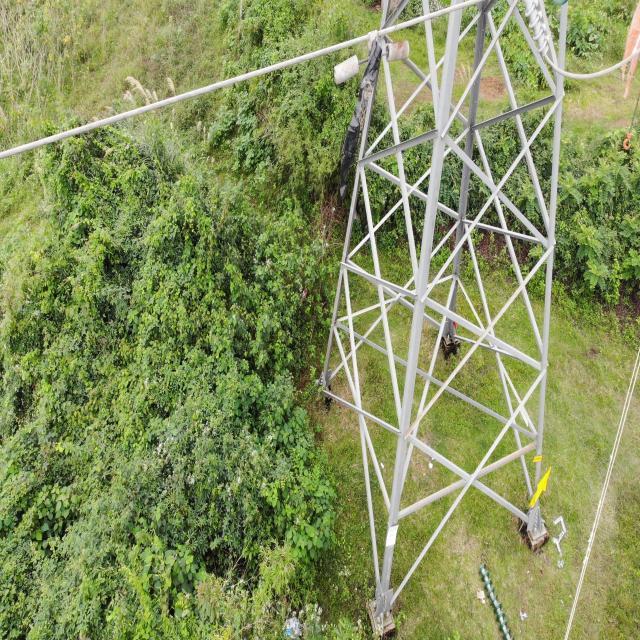

Supplement: Data S1 [file peerj-cs-10-2383-s001.zip › JPEGImages/DJI_0938_JPG.jpg]

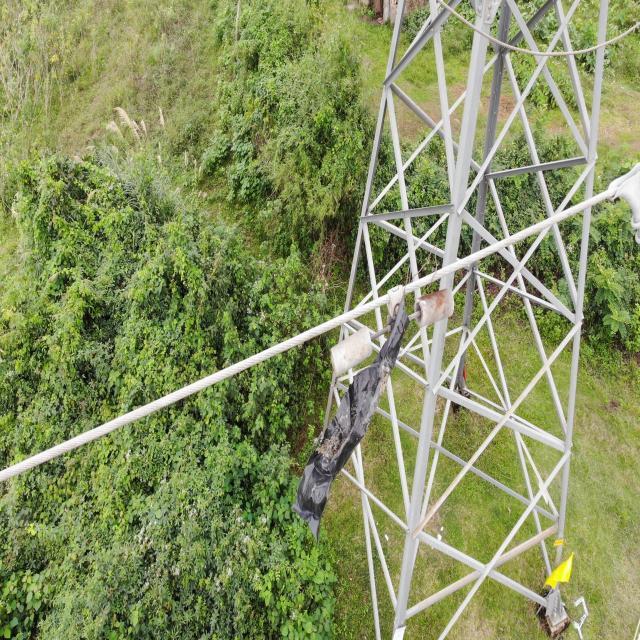

Supplement: Data S1 [file peerj-cs-10-2383-s001.zip › JPEGImages/DJI_0940_JPG.jpg]

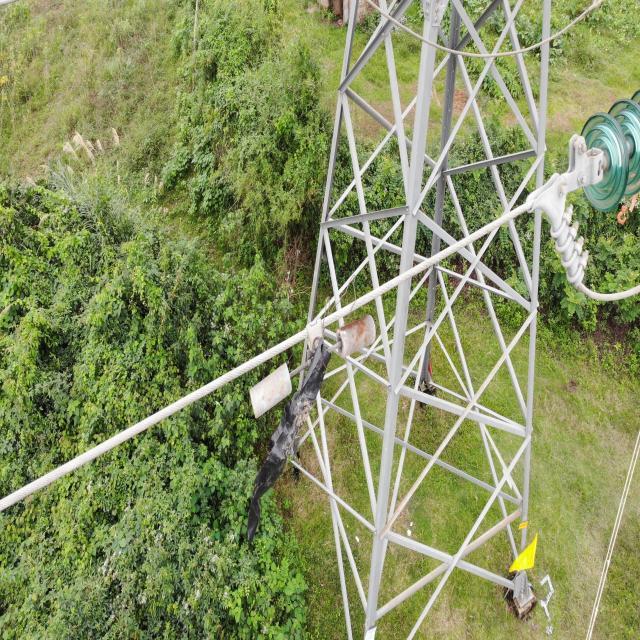

Supplement: Data S1 [file peerj-cs-10-2383-s001.zip › JPEGImages/DJI_0942_JPG.jpg]

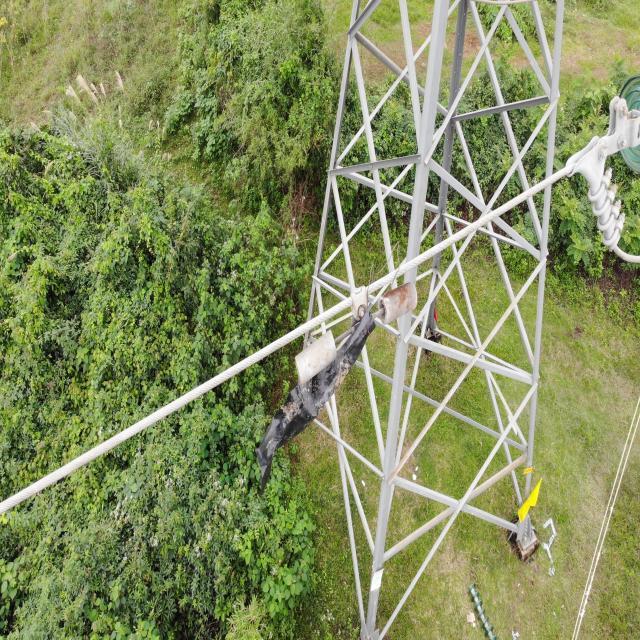

Supplement: Data S1 [file peerj-cs-10-2383-s001.zip › JPEGImages/DJI_0944_JPG.jpg]

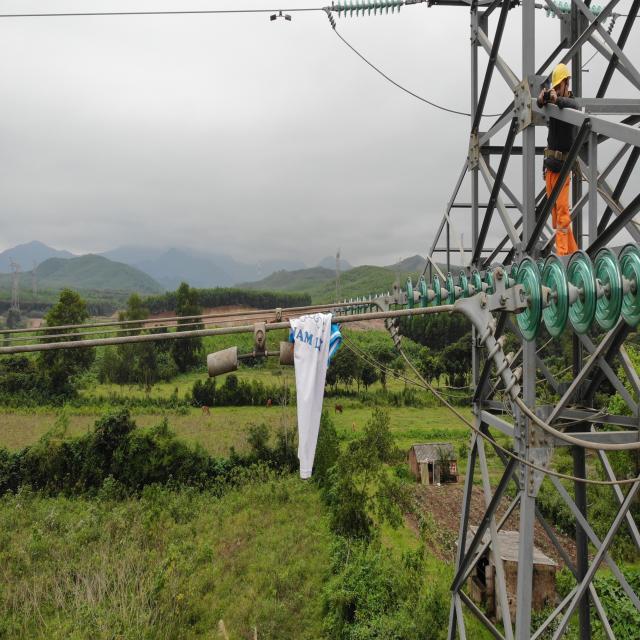

Supplement: Data S1 [file peerj-cs-10-2383-s001.zip › JPEGImages/DJI_0946_JPG.jpg]

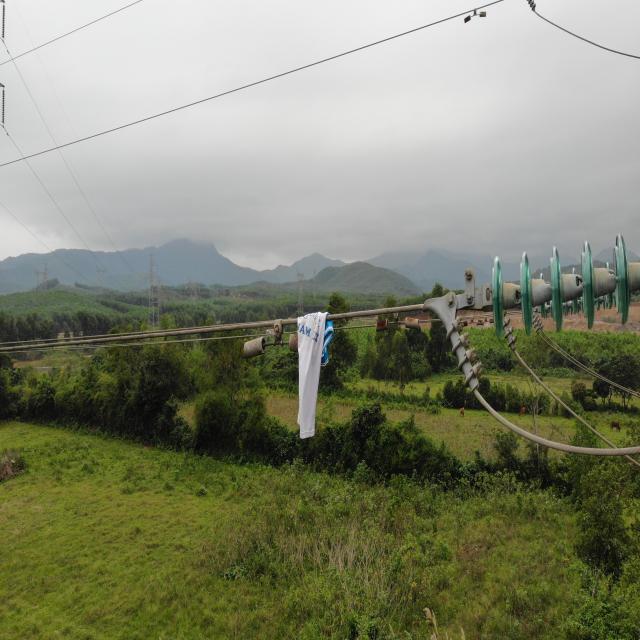

Supplement: Data S1 [file peerj-cs-10-2383-s001.zip › JPEGImages/DJI_0950_JPG.jpg]

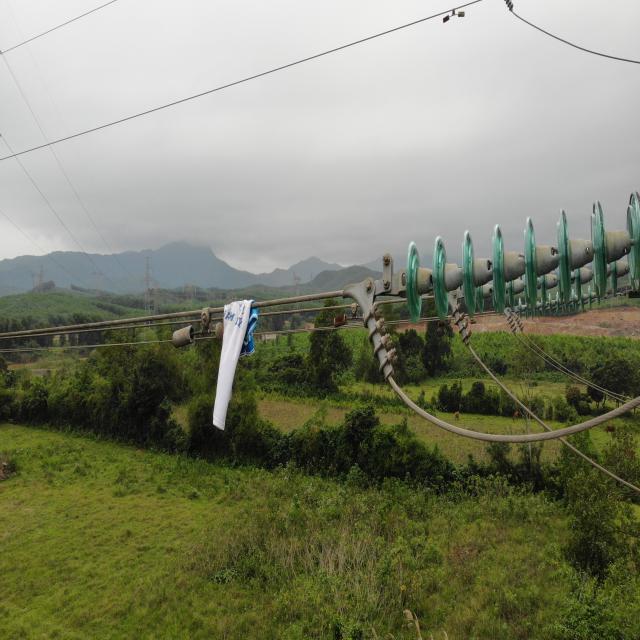

Supplement: Data S1 [file peerj-cs-10-2383-s001.zip › JPEGImages/DJI_0952_JPG.jpg]

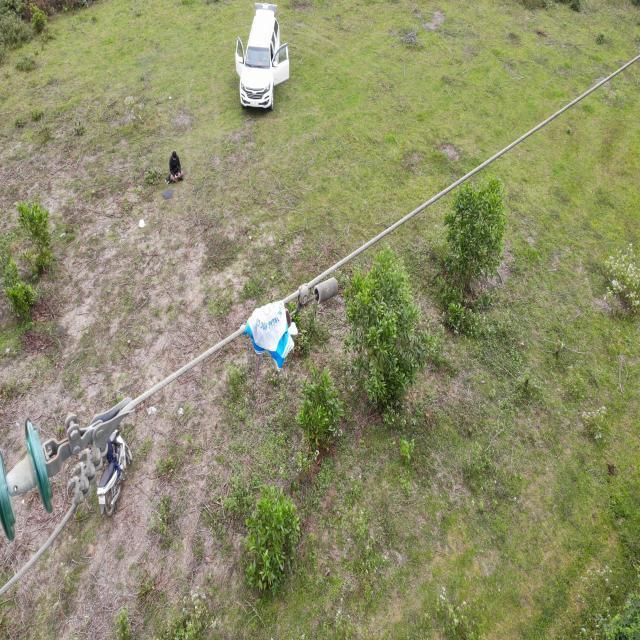

Supplement: Data S1 [file peerj-cs-10-2383-s001.zip › JPEGImages/DJI_0964_JPG.jpg]

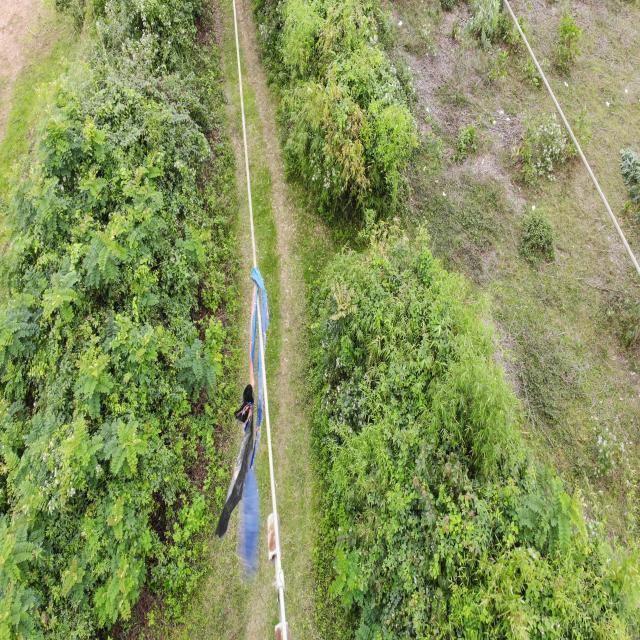

Supplement: Data S1 [file peerj-cs-10-2383-s001.zip › JPEGImages/DJI_0984_JPG.jpg]

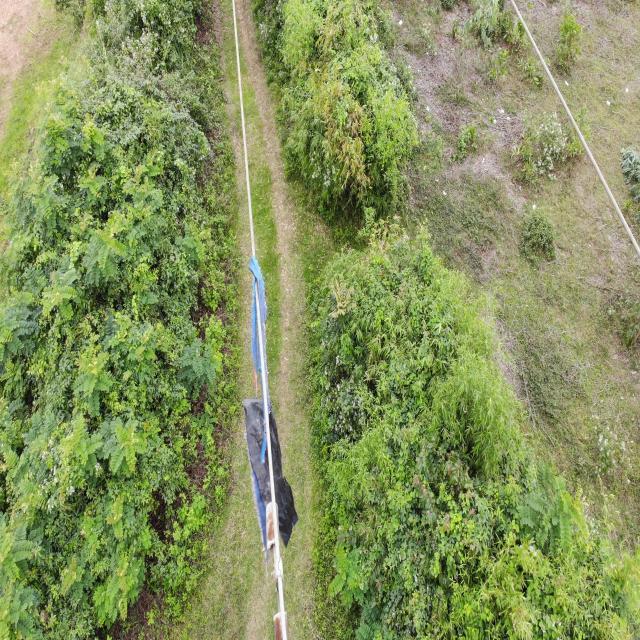

Supplement: Data S1 [file peerj-cs-10-2383-s001.zip › JPEGImages/DJI_0986_JPG.jpg]

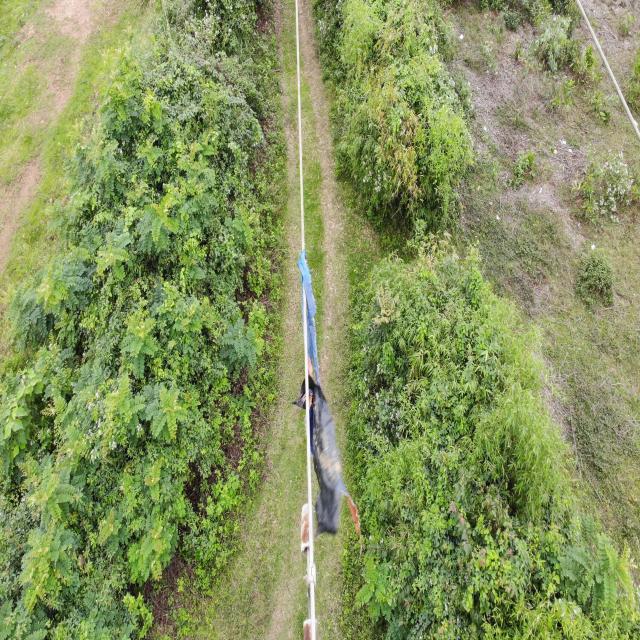

Supplement: Data S1 [file peerj-cs-10-2383-s001.zip › JPEGImages/DJI_0988_JPG.jpg]

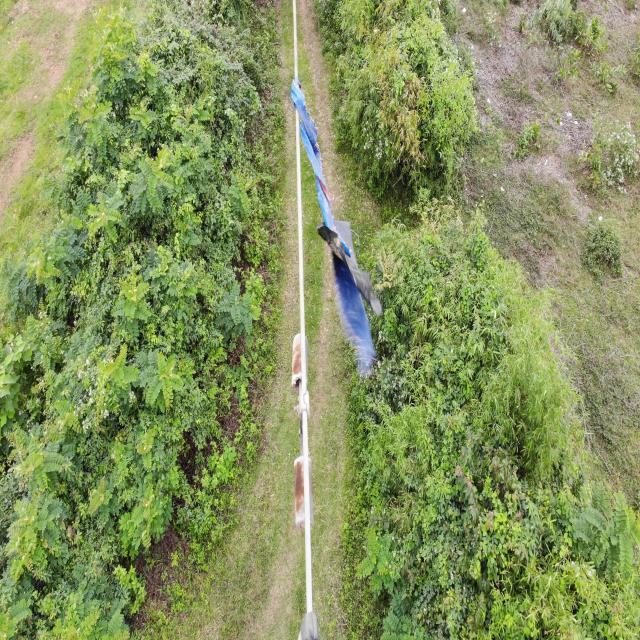

Supplement: Data S1 [file peerj-cs-10-2383-s001.zip › JPEGImages/DJI_0990_JPG.jpg]

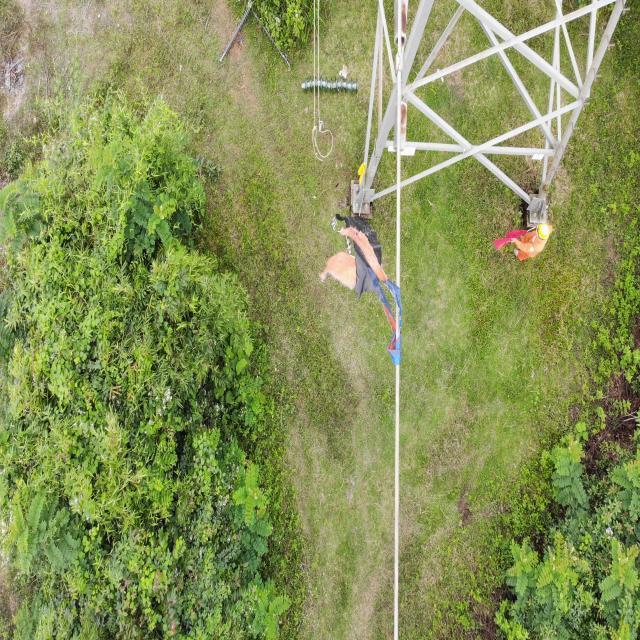

Supplement: Data S1 [file peerj-cs-10-2383-s001.zip › JPEGImages/DJI_0992_JPG.jpg]

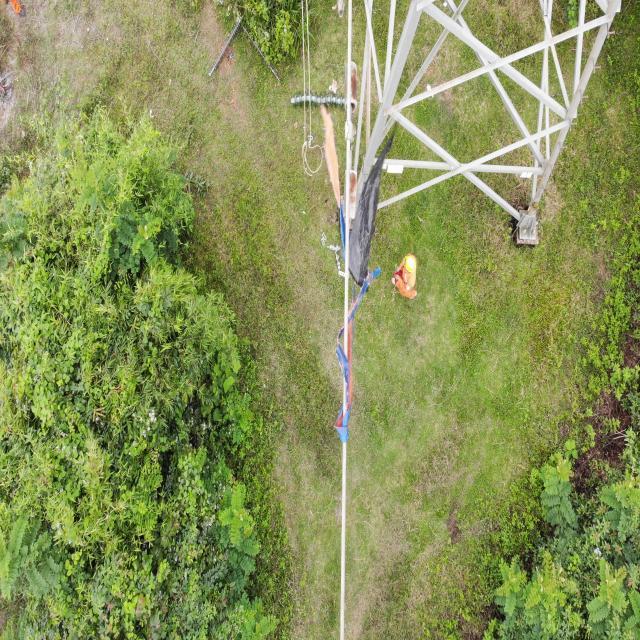

Supplement: Data S1 [file peerj-cs-10-2383-s001.zip › JPEGImages/DJI_0994_JPG.jpg]

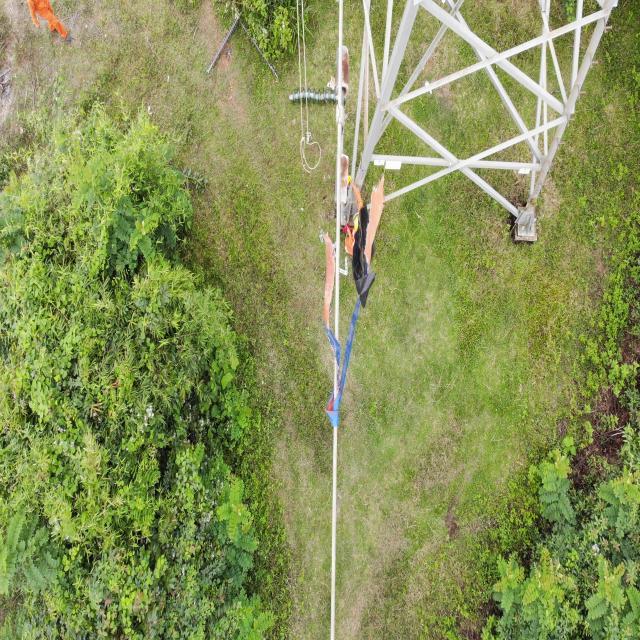

Supplement: Data S1 [file peerj-cs-10-2383-s001.zip › JPEGImages/DJI_0996_JPG.jpg]

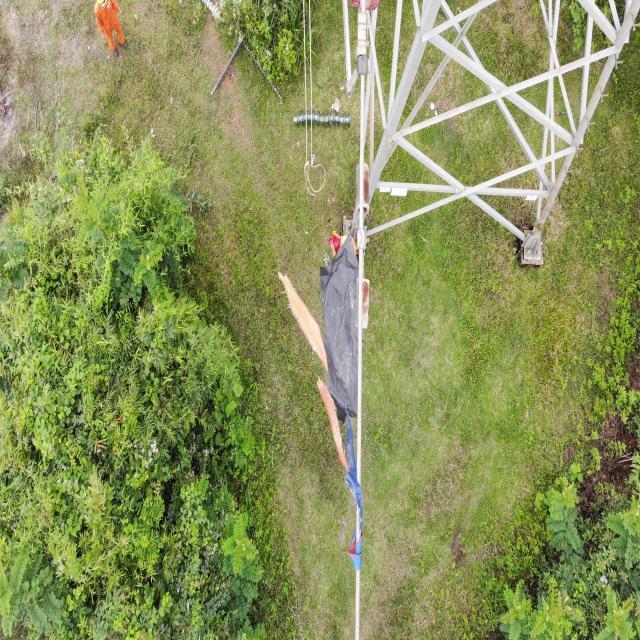

Supplement: Data S1 [file peerj-cs-10-2383-s001.zip › JPEGImages/DJI_0998_JPG.jpg]

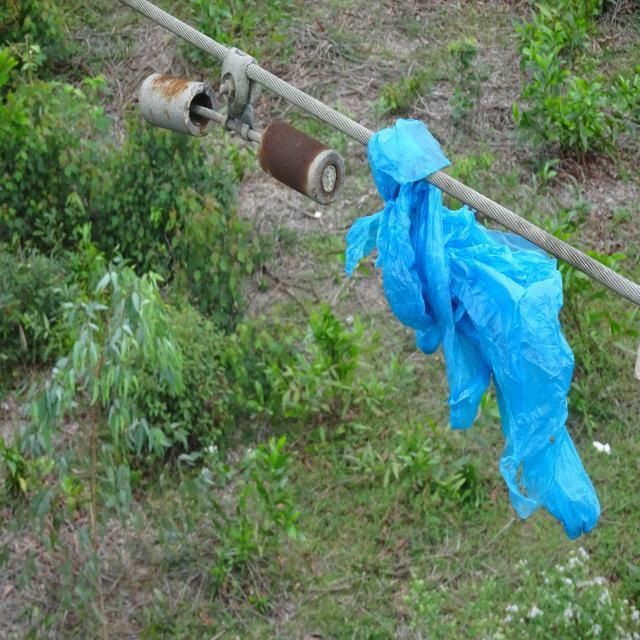

Supplement: Data S1 [file peerj-cs-10-2383-s001.zip › JPEGImages/DSC01423_JPG.jpg]

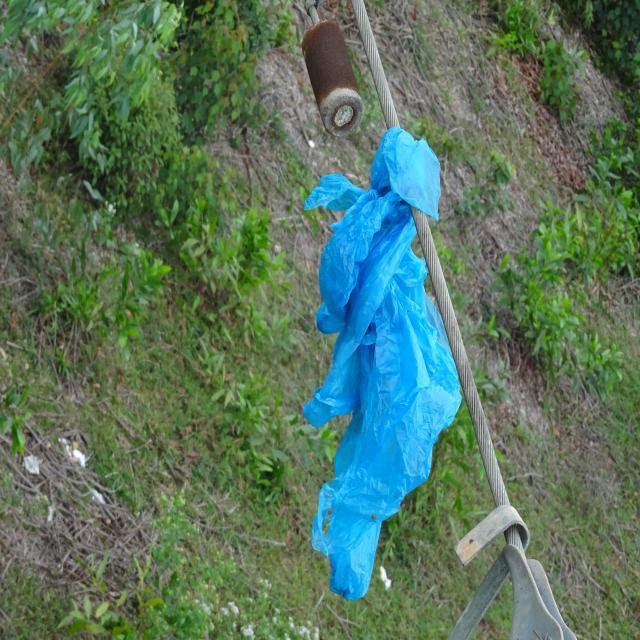

Supplement: Data S1 [file peerj-cs-10-2383-s001.zip › JPEGImages/DSC01424_JPG.jpg]

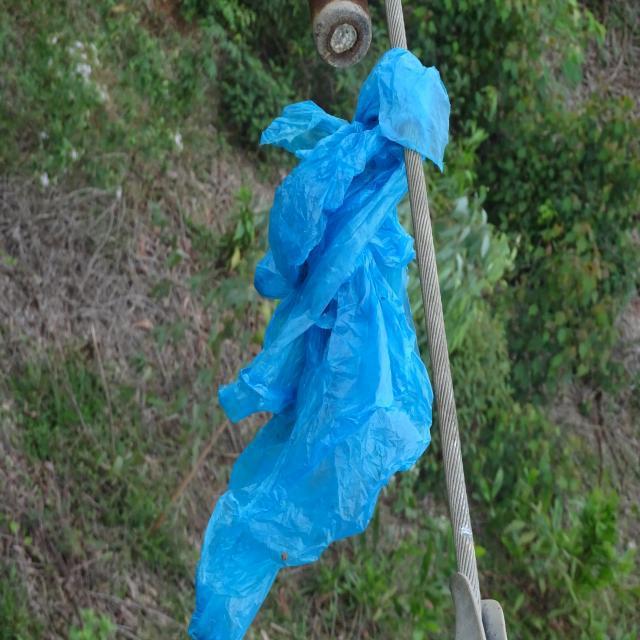

Supplement: Data S1 [file peerj-cs-10-2383-s001.zip › JPEGImages/DSC01425_JPG.jpg]

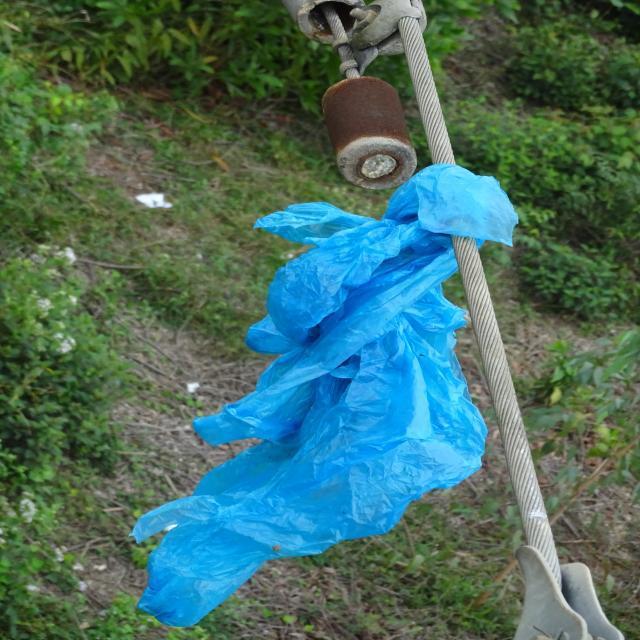

Supplement: Data S1 [file peerj-cs-10-2383-s001.zip › JPEGImages/DSC01426_JPG.jpg]

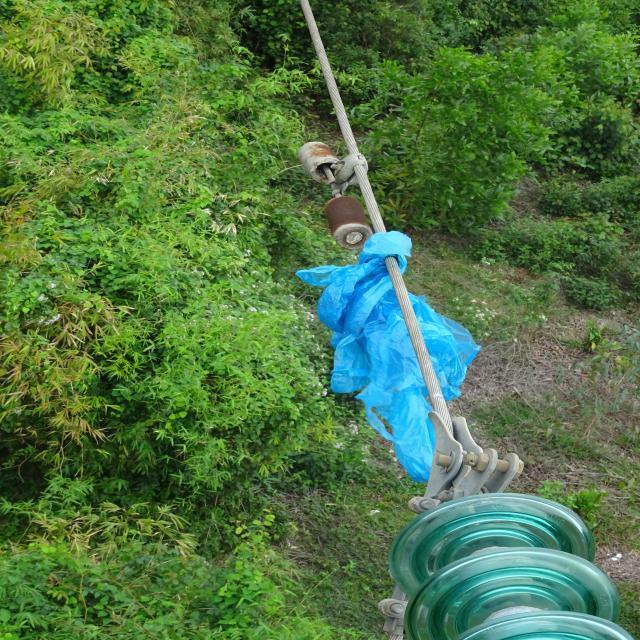

Supplement: Data S1 [file peerj-cs-10-2383-s001.zip › JPEGImages/DSC01427_JPG.jpg]

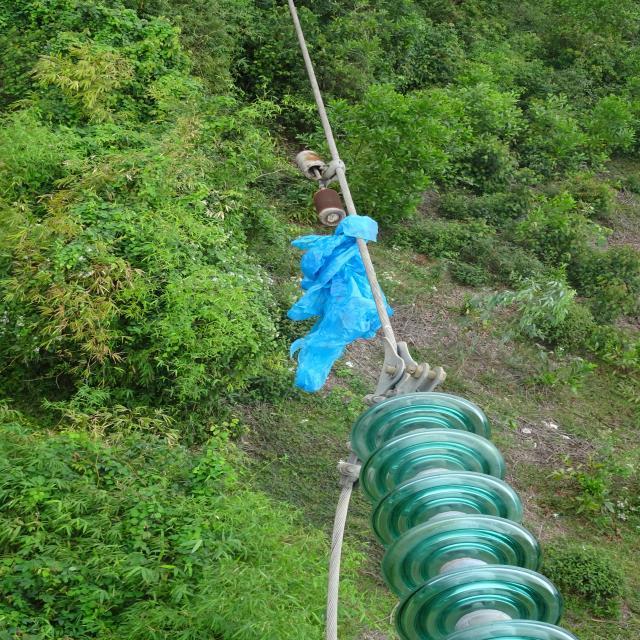

Supplement: Data S1 [file peerj-cs-10-2383-s001.zip › JPEGImages/DSC01428_JPG.jpg]

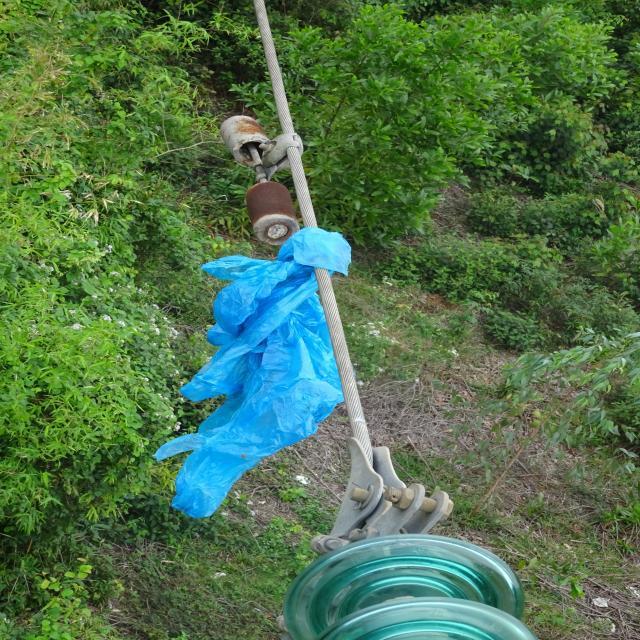

Supplement: Data S1 [file peerj-cs-10-2383-s001.zip › JPEGImages/DSC01429_JPG.jpg]

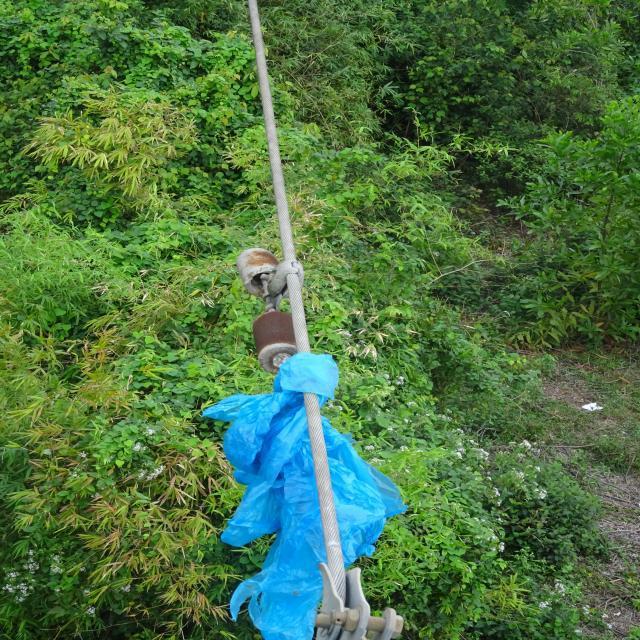

Supplement: Data S1 [file peerj-cs-10-2383-s001.zip › JPEGImages/DSC01430_JPG.jpg]

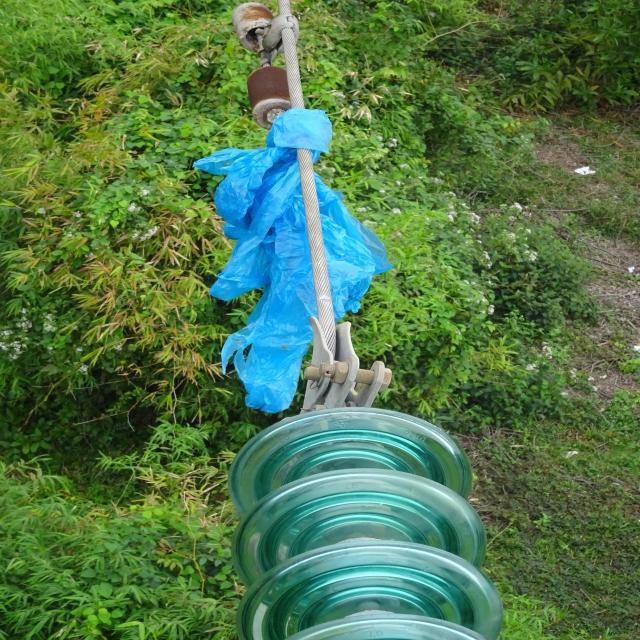

Supplement: Data S1 [file peerj-cs-10-2383-s001.zip › JPEGImages/DSC01431_JPG.jpg]

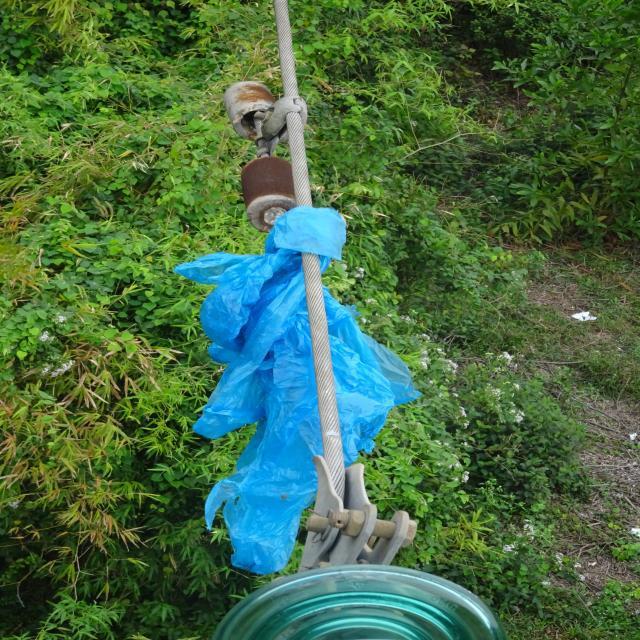

Supplement: Data S1 [file peerj-cs-10-2383-s001.zip › JPEGImages/DSC01432_JPG.jpg]

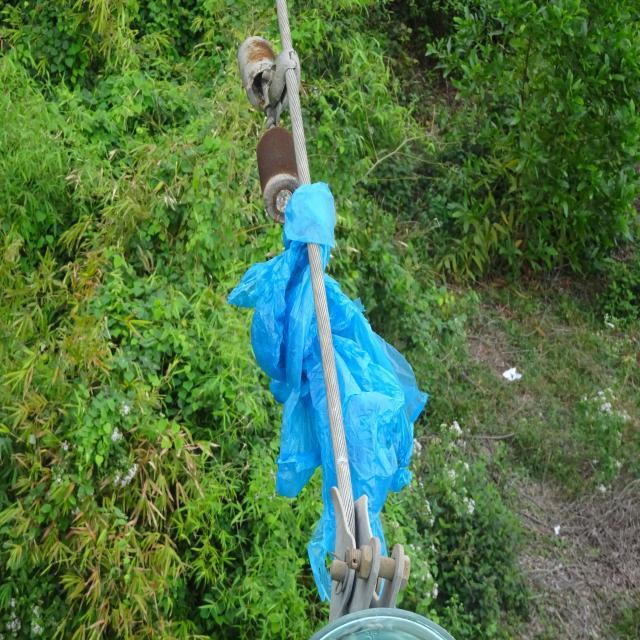

Supplement: Data S1 [file peerj-cs-10-2383-s001.zip › JPEGImages/DSC01433_JPG.jpg]

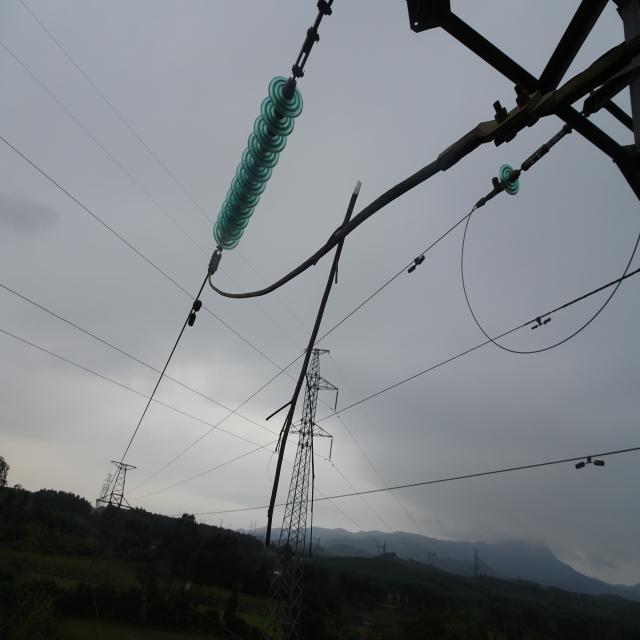

Supplement: Data S1 [file peerj-cs-10-2383-s001.zip › JPEGImages/DSC01434_JPG.jpg]

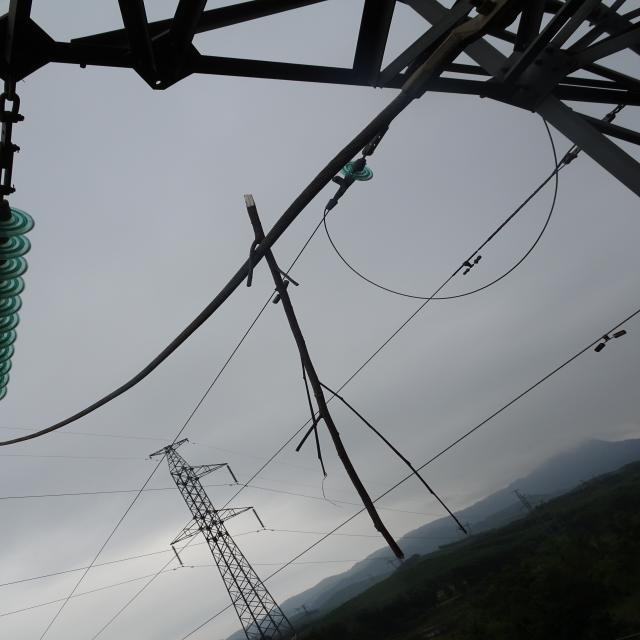

Supplement: Data S1 [file peerj-cs-10-2383-s001.zip › JPEGImages/DSC01435_JPG.jpg]

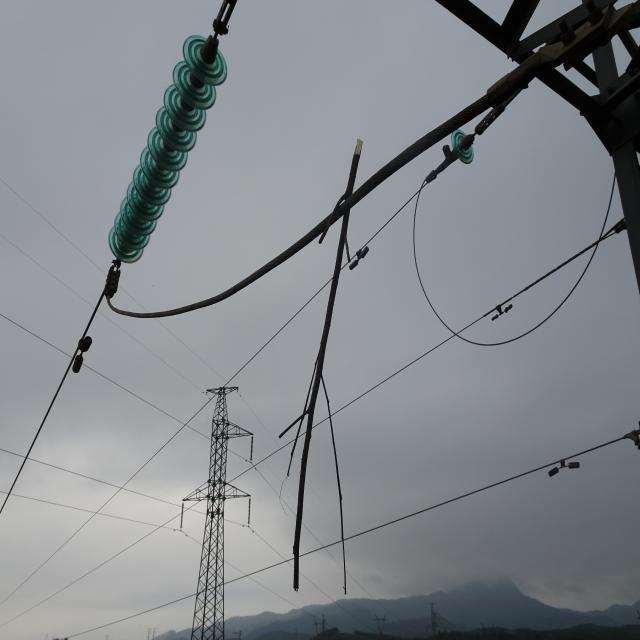

Supplement: Data S1 [file peerj-cs-10-2383-s001.zip › JPEGImages/DSC01436_JPG.jpg]

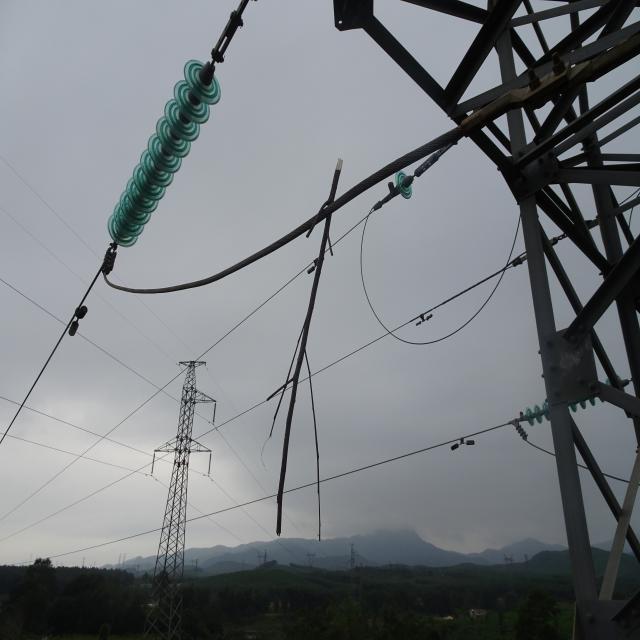

Supplement: Data S1 [file peerj-cs-10-2383-s001.zip › JPEGImages/DSC01437_JPG.jpg]

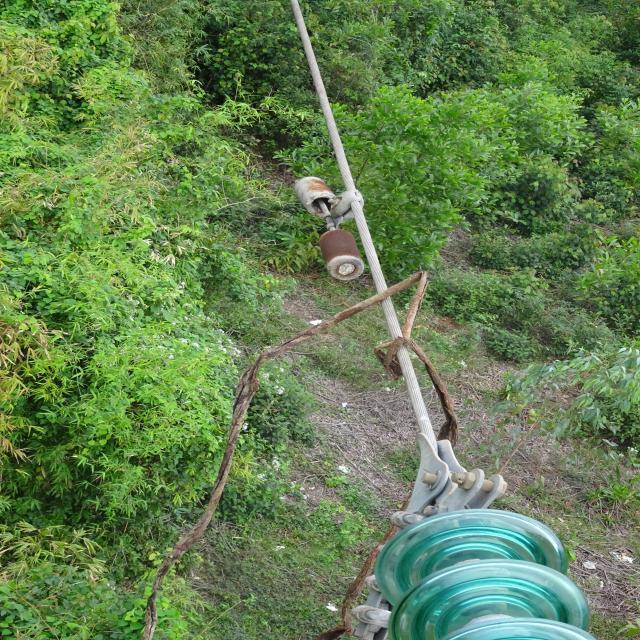

Supplement: Data S1 [file peerj-cs-10-2383-s001.zip › JPEGImages/DSC01438_JPG.jpg]

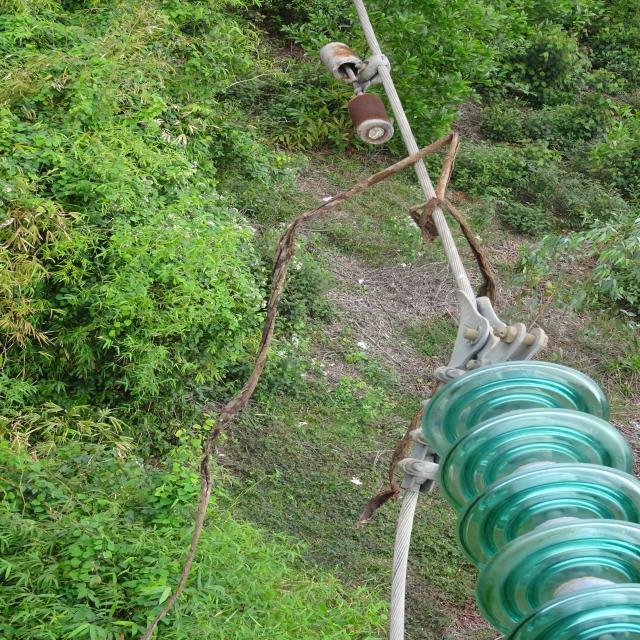

Supplement: Data S1 [file peerj-cs-10-2383-s001.zip › JPEGImages/DSC01439_JPG.jpg]

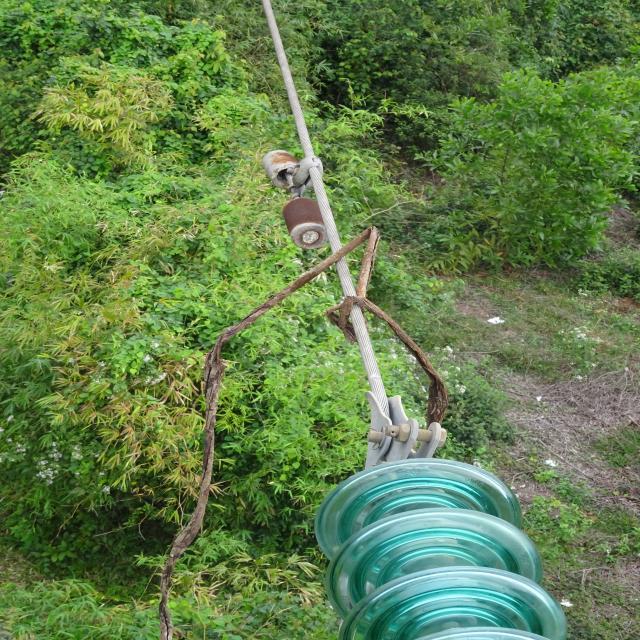

Supplement: Data S1 [file peerj-cs-10-2383-s001.zip › JPEGImages/DSC01440_JPG.jpg]

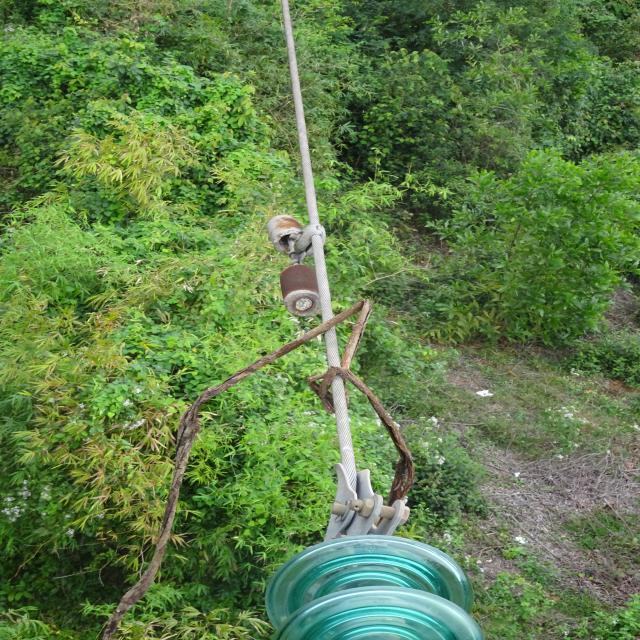

Supplement: Data S1 [file peerj-cs-10-2383-s001.zip › JPEGImages/DSC01441_JPG.jpg]

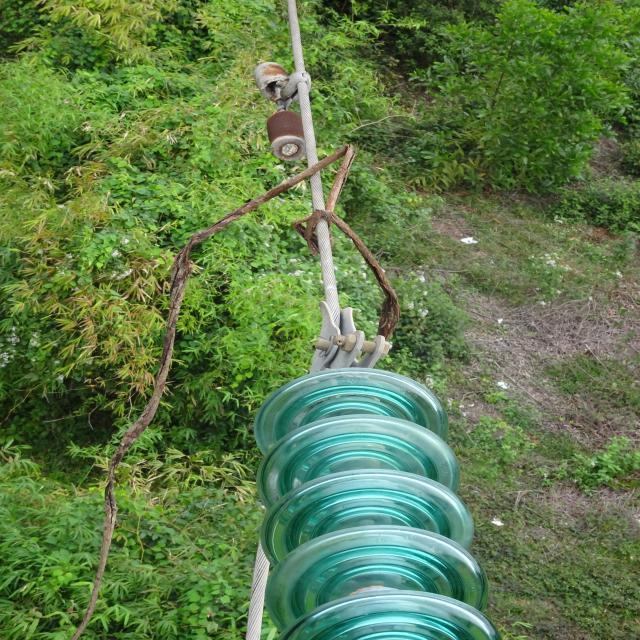

Supplement: Data S1 [file peerj-cs-10-2383-s001.zip › JPEGImages/DSC01442_JPG.jpg]

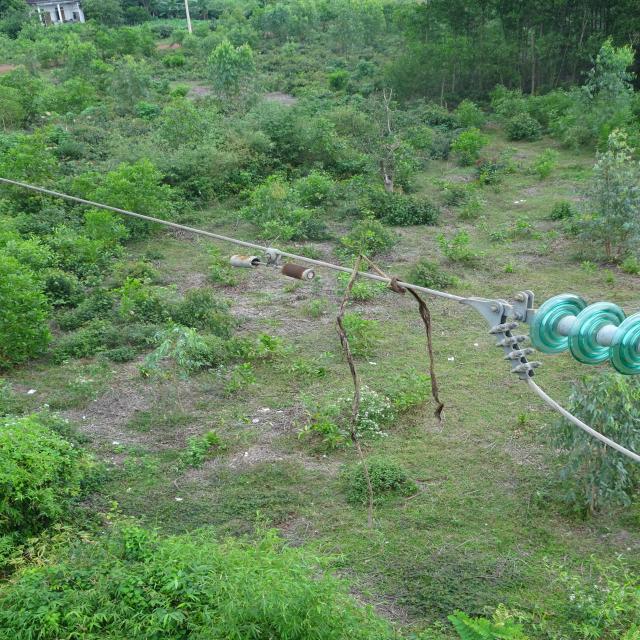

Supplement: Data S1 [file peerj-cs-10-2383-s001.zip › JPEGImages/DSC01443_JPG.jpg]

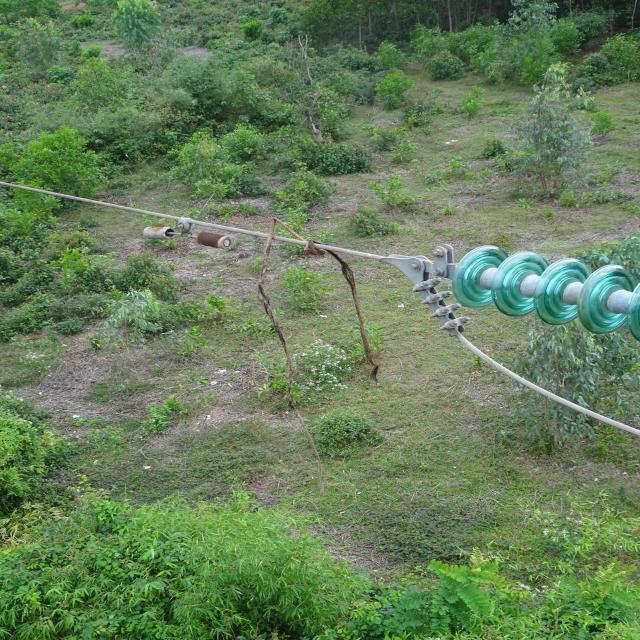

Supplement: Data S1 [file peerj-cs-10-2383-s001.zip › JPEGImages/DSC01444_JPG.jpg]

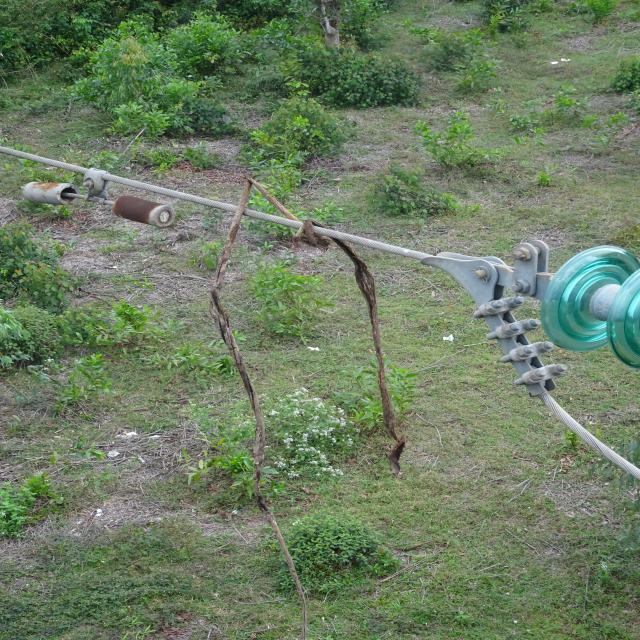

Supplement: Data S1 [file peerj-cs-10-2383-s001.zip › JPEGImages/DSC01445_JPG.jpg]

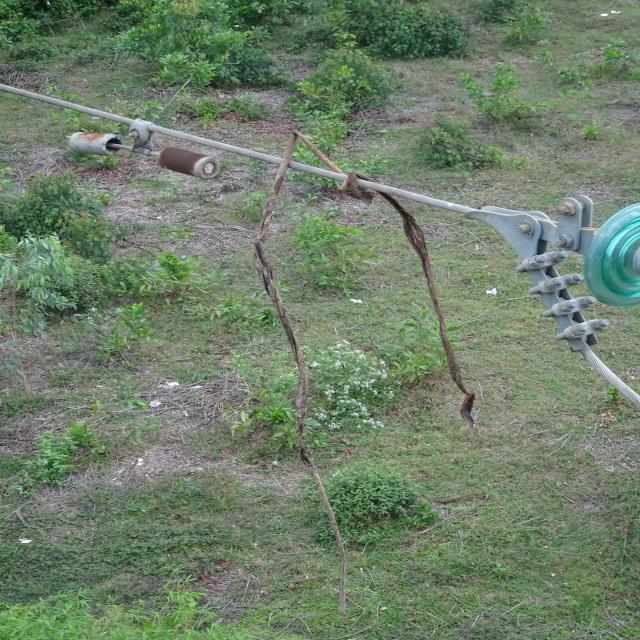

Supplement: Data S1 [file peerj-cs-10-2383-s001.zip › JPEGImages/DSC01446_JPG.jpg]

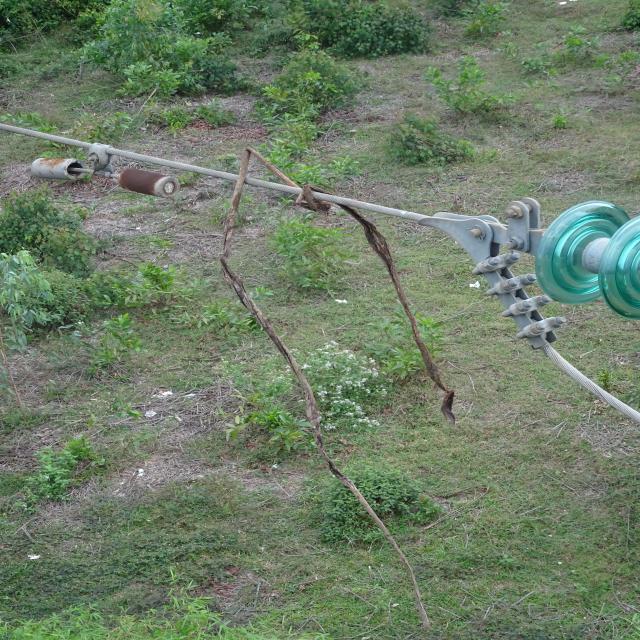

Supplement: Data S1 [file peerj-cs-10-2383-s001.zip › JPEGImages/DSC01447_JPG.jpg]

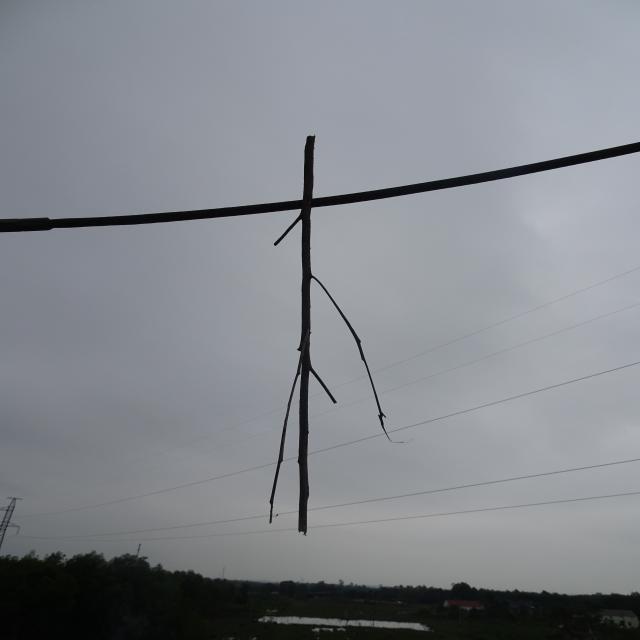

Supplement: Data S1 [file peerj-cs-10-2383-s001.zip › JPEGImages/DSC01448_JPG.jpg]

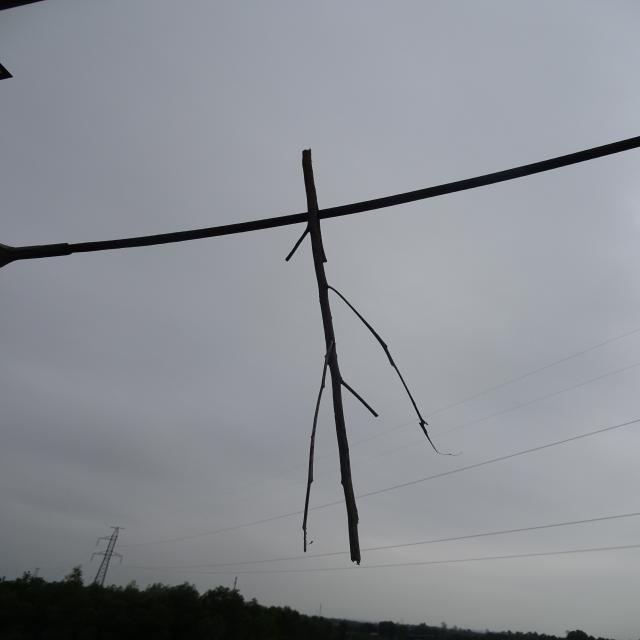

Supplement: Data S1 [file peerj-cs-10-2383-s001.zip › JPEGImages/DSC01449_JPG.jpg]

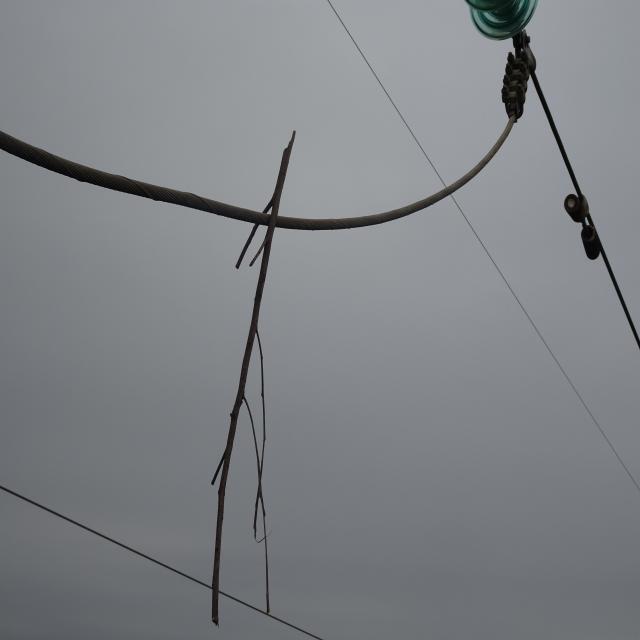

Supplement: Data S1 [file peerj-cs-10-2383-s001.zip › JPEGImages/DSC01452_JPG.jpg]

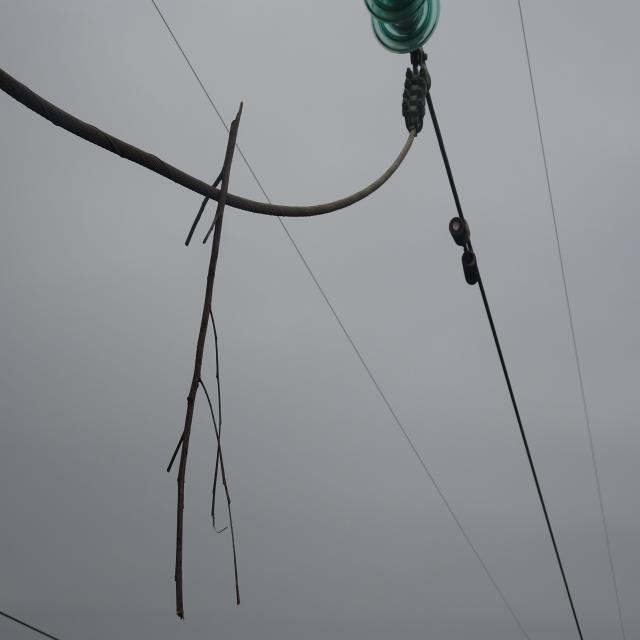

Supplement: Data S1 [file peerj-cs-10-2383-s001.zip › JPEGImages/DSC01453_JPG.jpg]

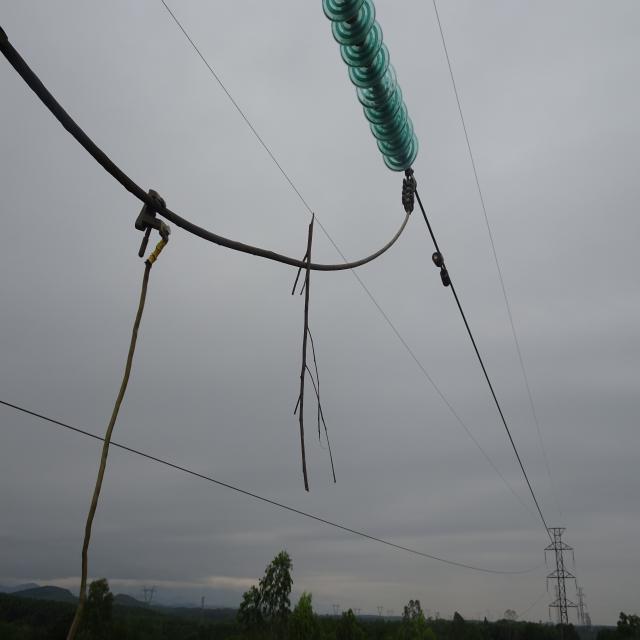

Supplement: Data S1 [file peerj-cs-10-2383-s001.zip › JPEGImages/DSC01454_JPG.jpg]

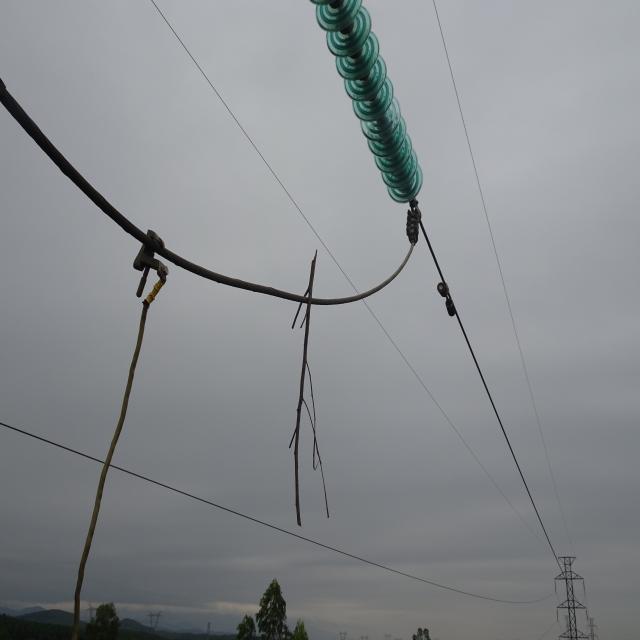

Supplement: Data S1 [file peerj-cs-10-2383-s001.zip › JPEGImages/DSC01455_JPG.jpg]

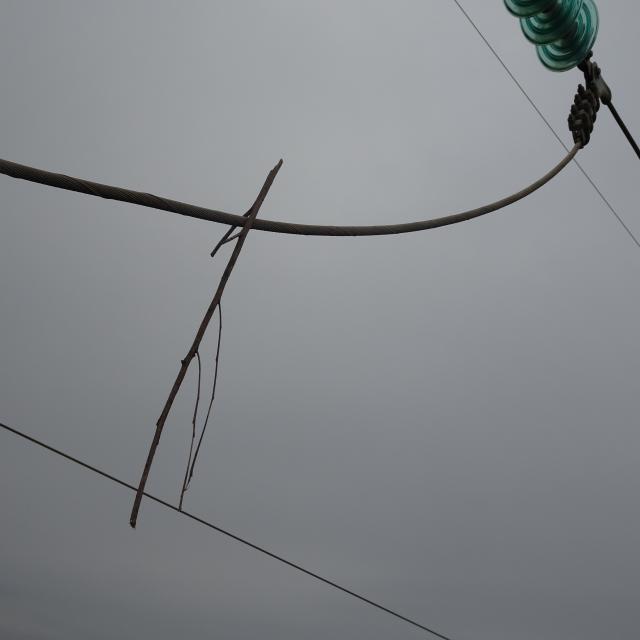

Supplement: Data S1 [file peerj-cs-10-2383-s001.zip › JPEGImages/DSC01456_JPG.jpg]

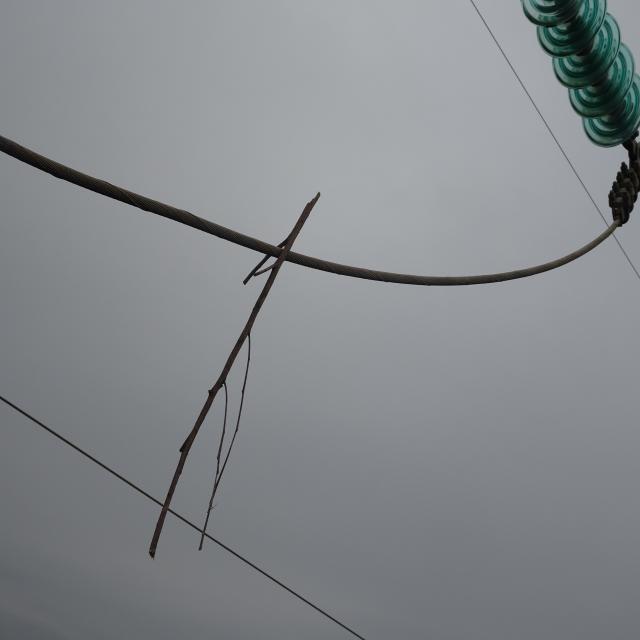

Supplement: Data S1 [file peerj-cs-10-2383-s001.zip › JPEGImages/DSC01457_JPG.jpg]

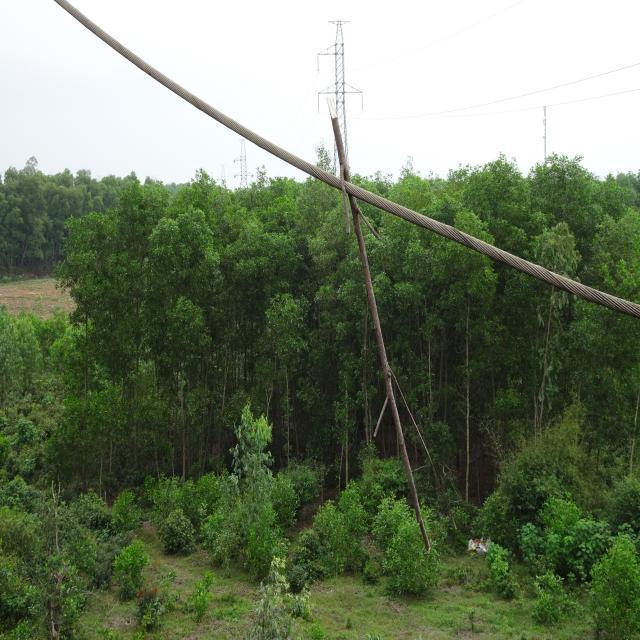

Supplement: Data S1 [file peerj-cs-10-2383-s001.zip › JPEGImages/DSC01482_JPG.jpg]

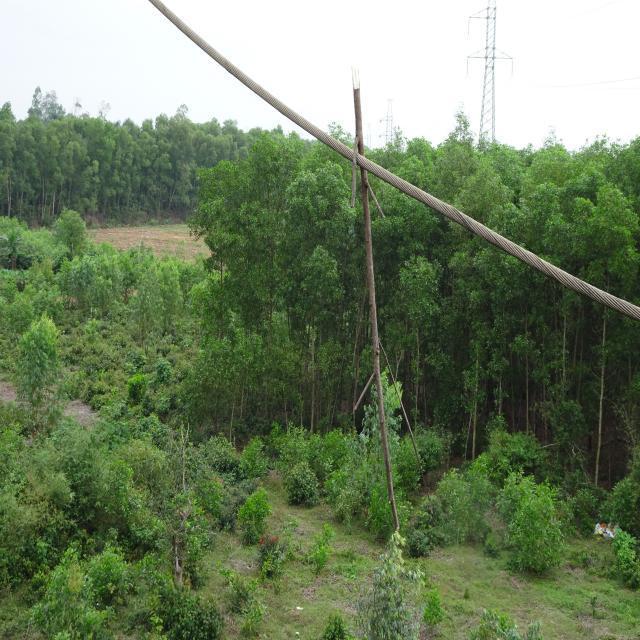

Supplement: Data S1 [file peerj-cs-10-2383-s001.zip › JPEGImages/DSC01483_JPG.jpg]

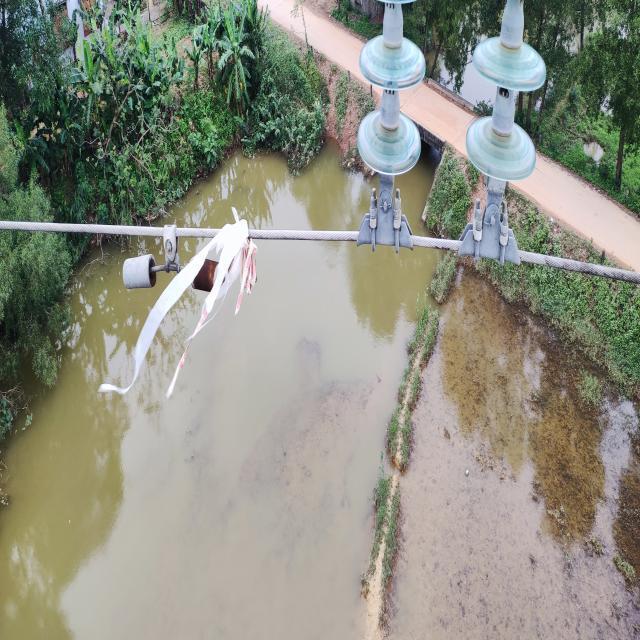

Supplement: Data S1 [file peerj-cs-10-2383-s001.zip › JPEGImages/IMG_20221208_085356_jpg.jpg]

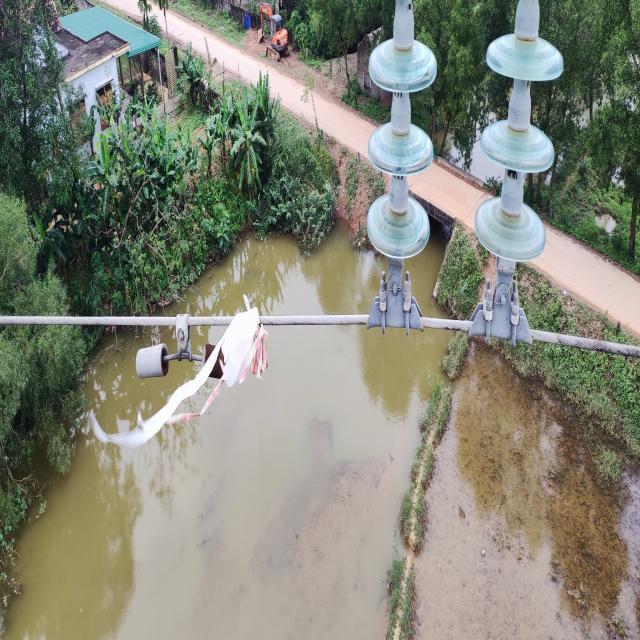

Supplement: Data S1 [file peerj-cs-10-2383-s001.zip › JPEGImages/IMG_20221208_085358_jpg.jpg]

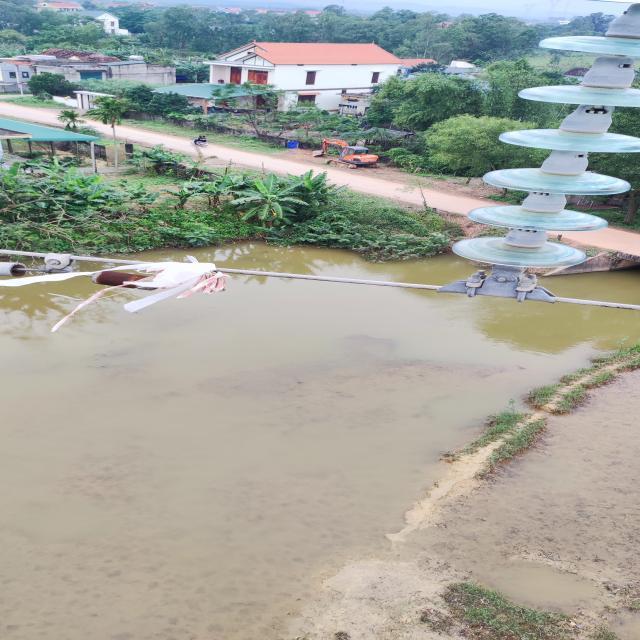

Supplement: Data S1 [file peerj-cs-10-2383-s001.zip › JPEGImages/IMG_20221208_085416_jpg.jpg]

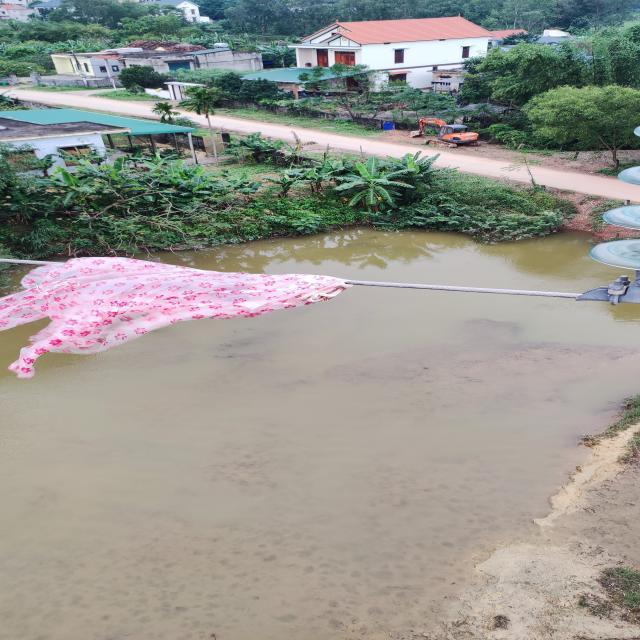

Supplement: Data S1 [file peerj-cs-10-2383-s001.zip › JPEGImages/IMG_20221208_085933_jpg.jpg]

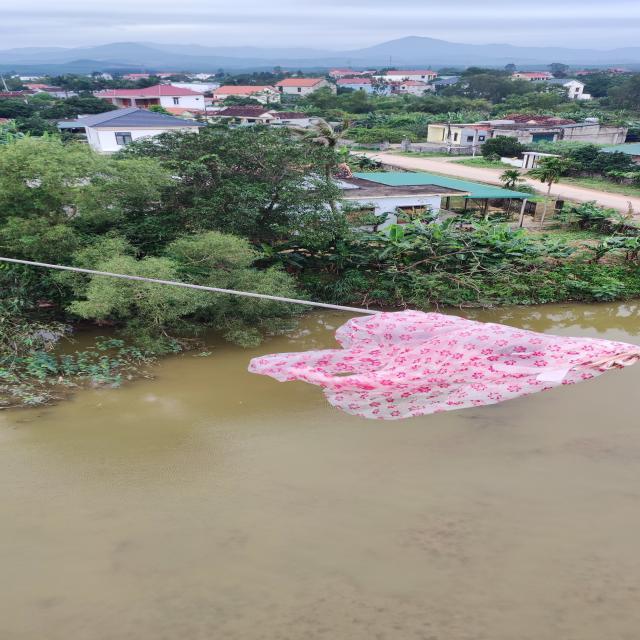

Supplement: Data S1 [file peerj-cs-10-2383-s001.zip › JPEGImages/IMG_20221208_085938_jpg.jpg]

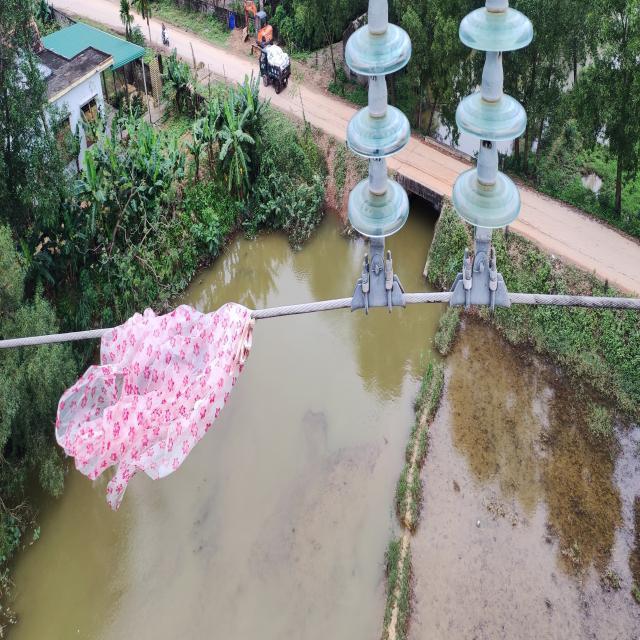

Supplement: Data S1 [file peerj-cs-10-2383-s001.zip › JPEGImages/IMG_20221208_085954_jpg.jpg]

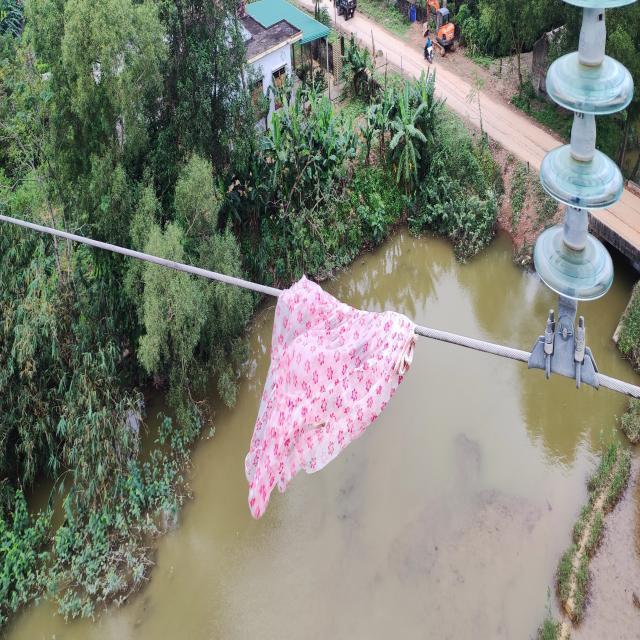

Supplement: Data S1 [file peerj-cs-10-2383-s001.zip › JPEGImages/IMG_20221208_090002_jpg.jpg]

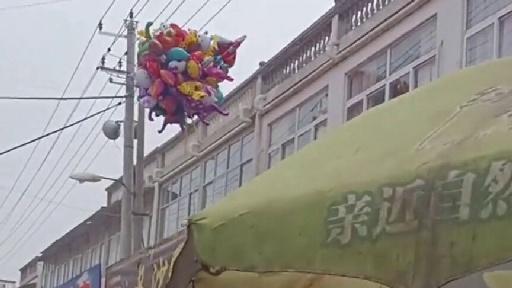

Supplement: Data S1 [file peerj-cs-10-2383-s001.zip › JPEGImages/balloon_234.jpg]

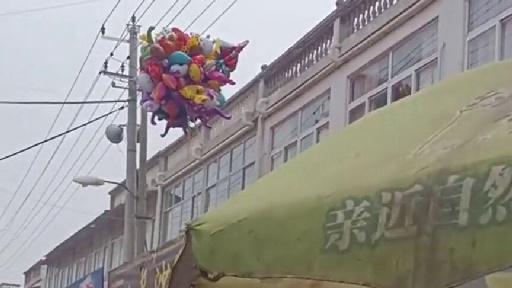

Supplement: Data S1 [file peerj-cs-10-2383-s001.zip › JPEGImages/balloon_235.jpg]

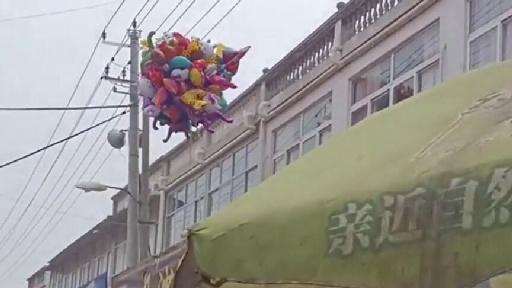

Supplement: Data S1 [file peerj-cs-10-2383-s001.zip › JPEGImages/balloon_236.jpg]

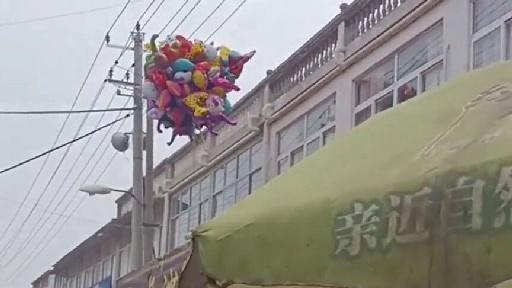

Supplement: Data S1 [file peerj-cs-10-2383-s001.zip › JPEGImages/balloon_237.jpg]

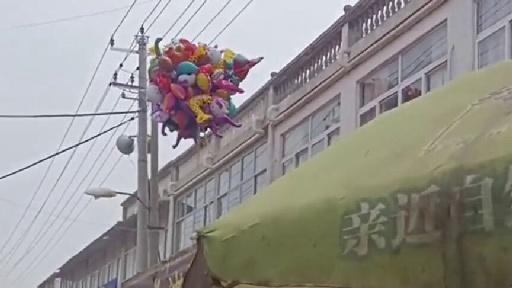

Supplement: Data S1 [file peerj-cs-10-2383-s001.zip › JPEGImages/balloon_238.jpg]

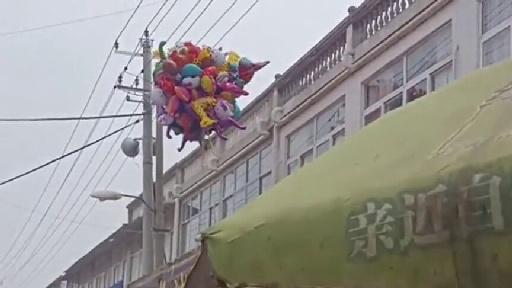

Supplement: Data S1 [file peerj-cs-10-2383-s001.zip › JPEGImages/balloon_239.jpg]

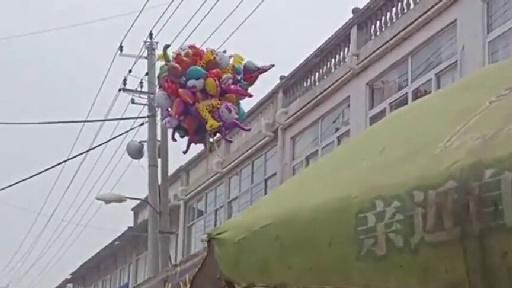

Supplement: Data S1 [file peerj-cs-10-2383-s001.zip › JPEGImages/balloon_240.jpg]

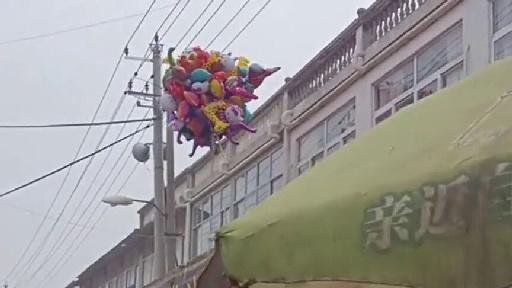

Supplement: Data S1 [file peerj-cs-10-2383-s001.zip › JPEGImages/balloon_241.jpg]

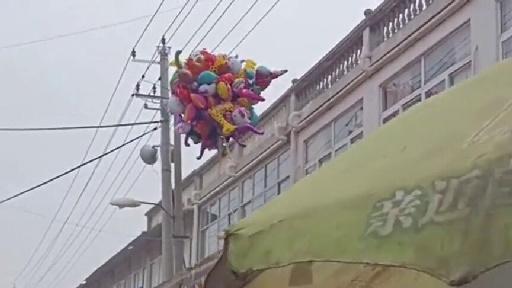

Supplement: Data S1 [file peerj-cs-10-2383-s001.zip › JPEGImages/balloon_242.jpg]

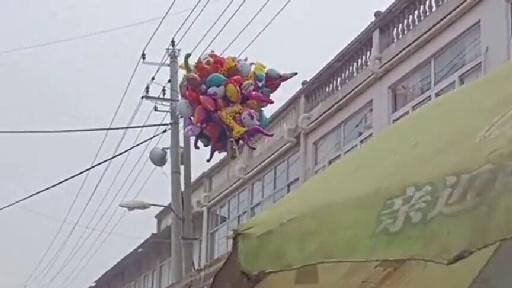

Supplement: Data S1 [file peerj-cs-10-2383-s001.zip › JPEGImages/balloon_243.jpg]

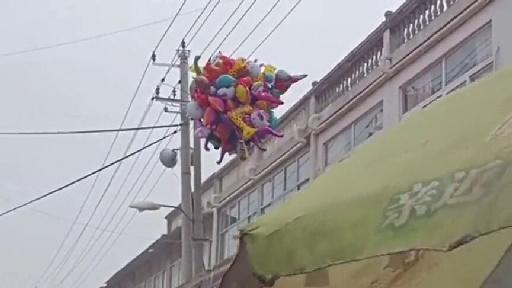

Supplement: Data S1 [file peerj-cs-10-2383-s001.zip › JPEGImages/balloon_244.jpg]

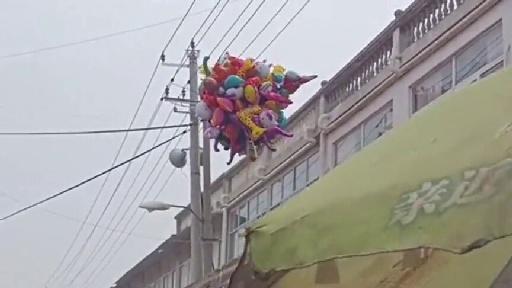

Supplement: Data S1 [file peerj-cs-10-2383-s001.zip › JPEGImages/balloon_245.jpg]

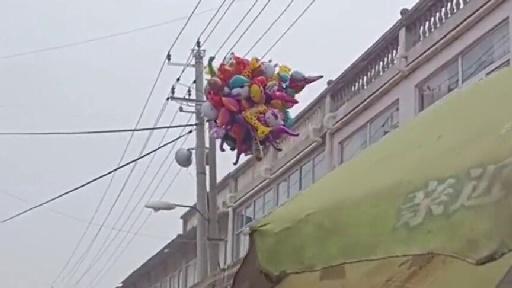

Supplement: Data S1 [file peerj-cs-10-2383-s001.zip › JPEGImages/balloon_246.jpg]

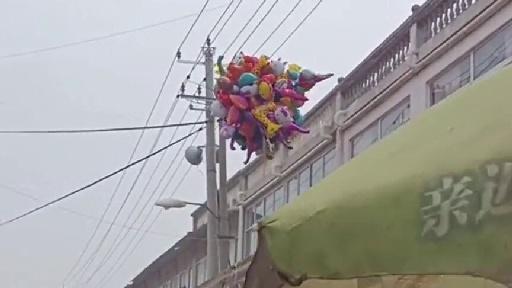

Supplement: Data S1 [file peerj-cs-10-2383-s001.zip › JPEGImages/balloon_247.jpg]

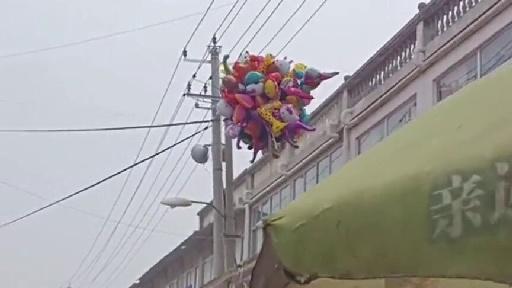

Supplement: Data S1 [file peerj-cs-10-2383-s001.zip › JPEGImages/balloon_248.jpg]
