# Supplementary figures and images for: Transmission line foreign object segmentation based on RB-UNet algorithm (part 1 of 2)
Source: PeerJ Comput Sci. 2024 Oct 10;10:e2383. doi: 10.7717/peerj-cs.2383 (PMC11622974; doi:10.7717/peerj-cs.2383)

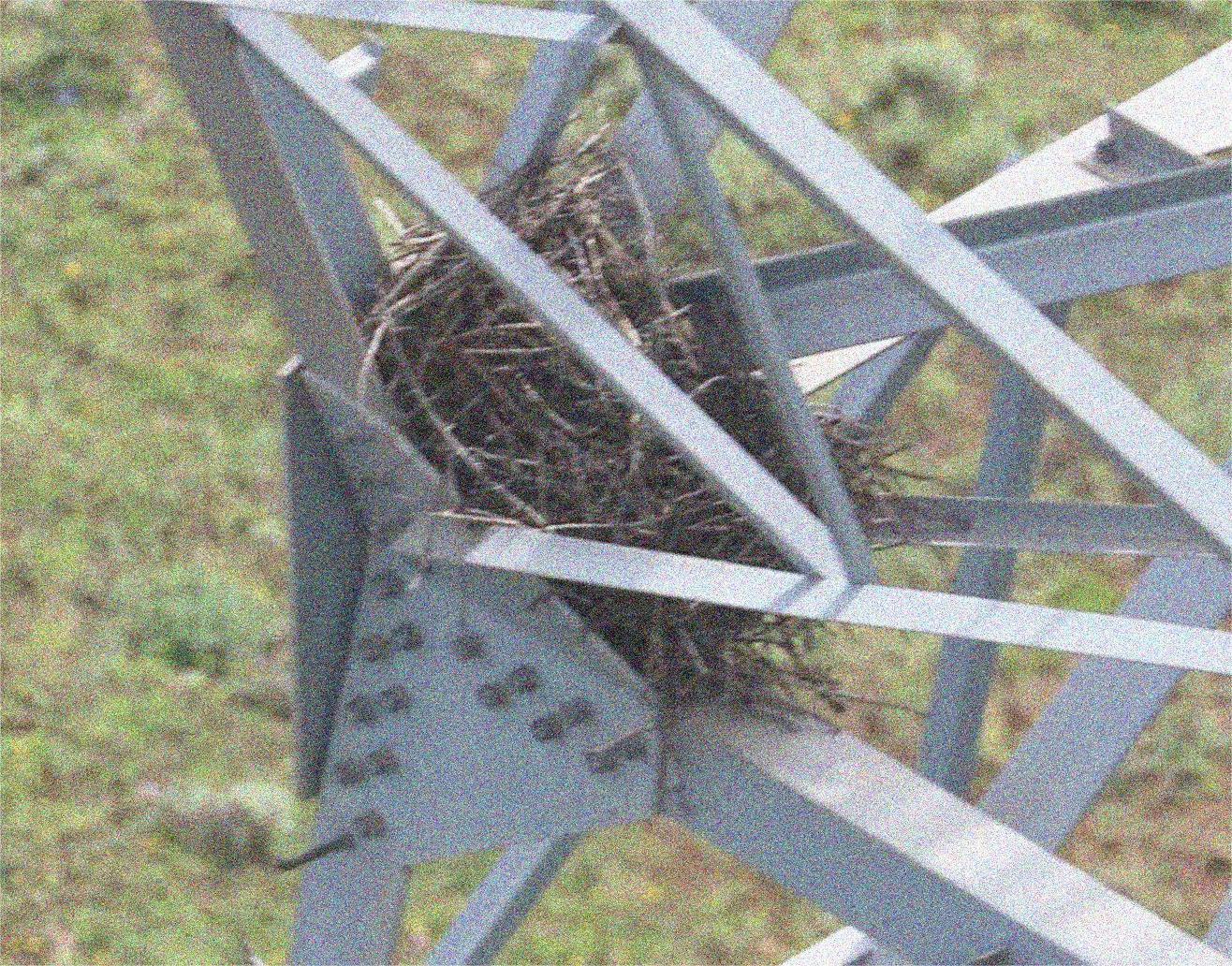

Supplement: Data S1 [file peerj-cs-10-2383-s001.zip › JPEGImages/0DLO5eu9cmov18kNWtgE2qFTfQChVU73rlJwp4SB.jpg]

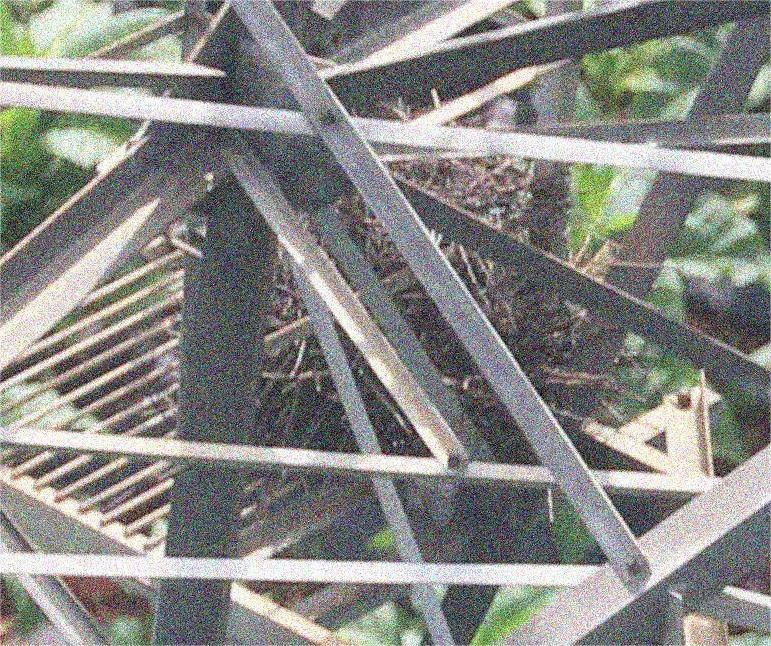

Supplement: Data S1 [file peerj-cs-10-2383-s001.zip › JPEGImages/0HTEZnvKuj1PAdqz236pUFgD5JoNracOIyXm9MSf.jpg]

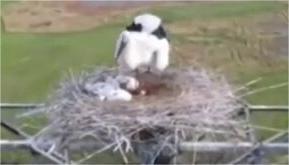

Supplement: Data S1 [file peerj-cs-10-2383-s001.zip › JPEGImages/0JbDO6c4Ga3y7eRTSBmZYqxzFwohfVjEKvMtigdN.jpg]

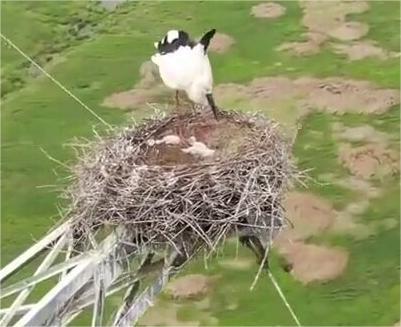

Supplement: Data S1 [file peerj-cs-10-2383-s001.zip › JPEGImages/0SHoFfw2T7byZlOAgIkC9XudijNGRxhrYpE5Qva6.jpg]

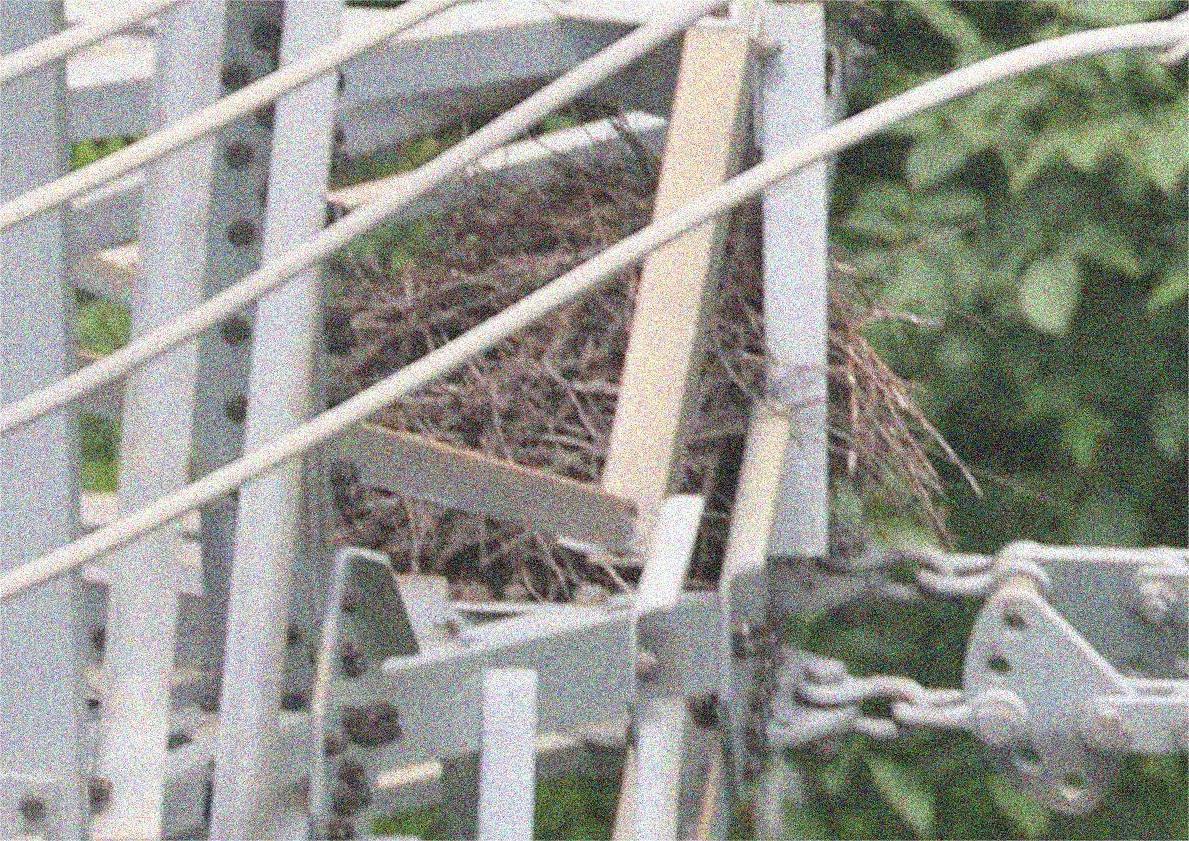

Supplement: Data S1 [file peerj-cs-10-2383-s001.zip › JPEGImages/0bEmQc2ksXjghIC8doyKp3AYlMDftB5RquPTLare.jpg]

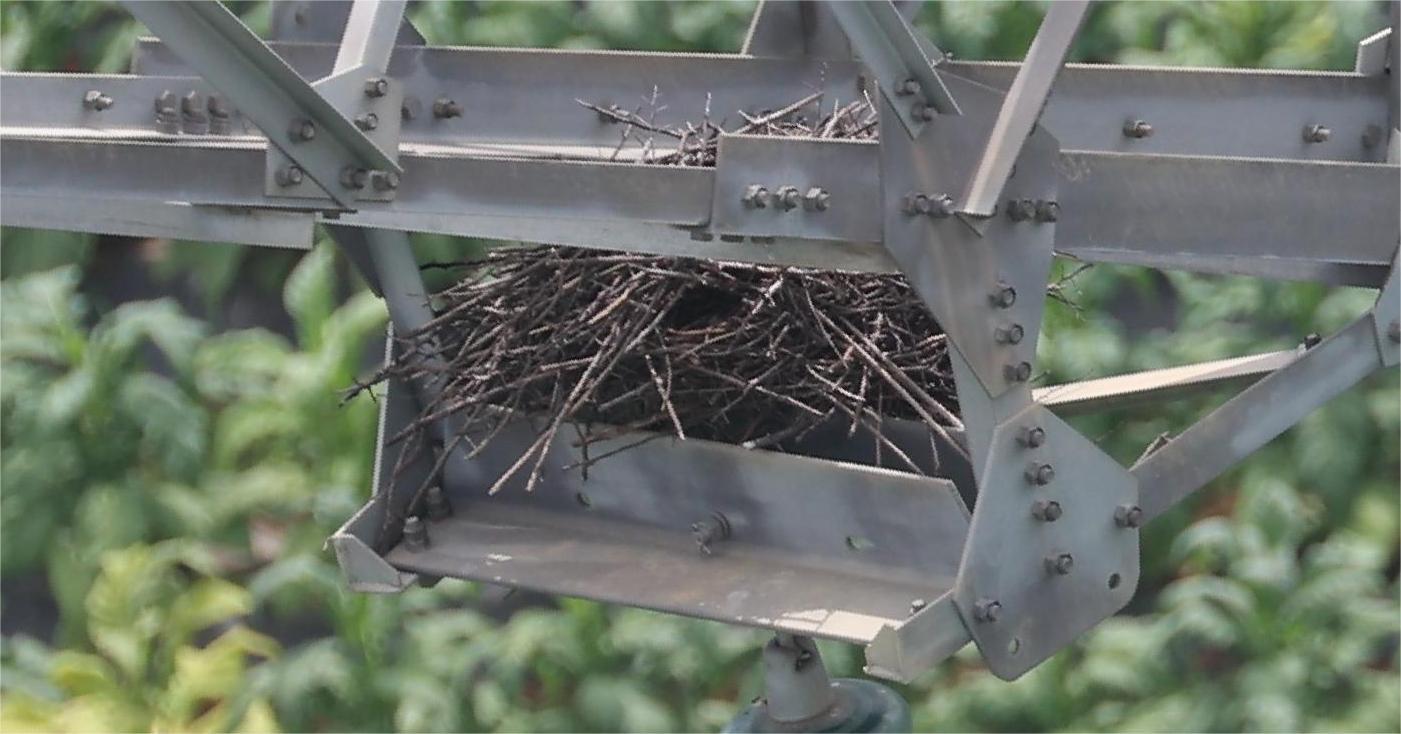

Supplement: Data S1 [file peerj-cs-10-2383-s001.zip › JPEGImages/0fhWXFA45IombLsqwNJu9kOtxMUP1YzeycH8gda6.jpg]

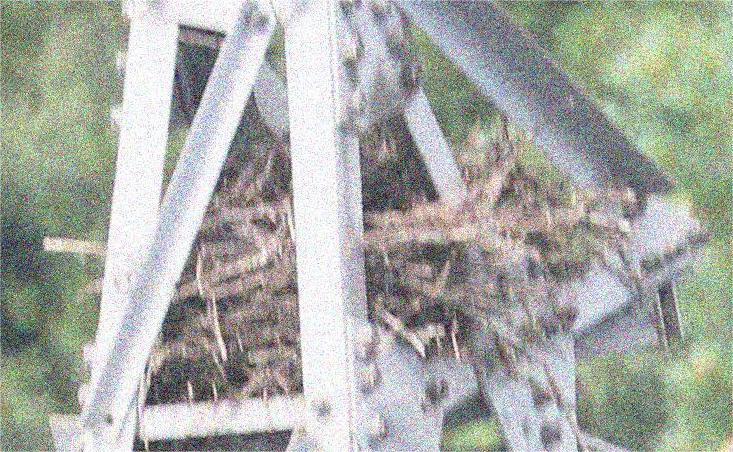

Supplement: Data S1 [file peerj-cs-10-2383-s001.zip › JPEGImages/0kcjRPVl9fpdamXTxWZnyhAioE2H6brLKISwFq7O.jpg]

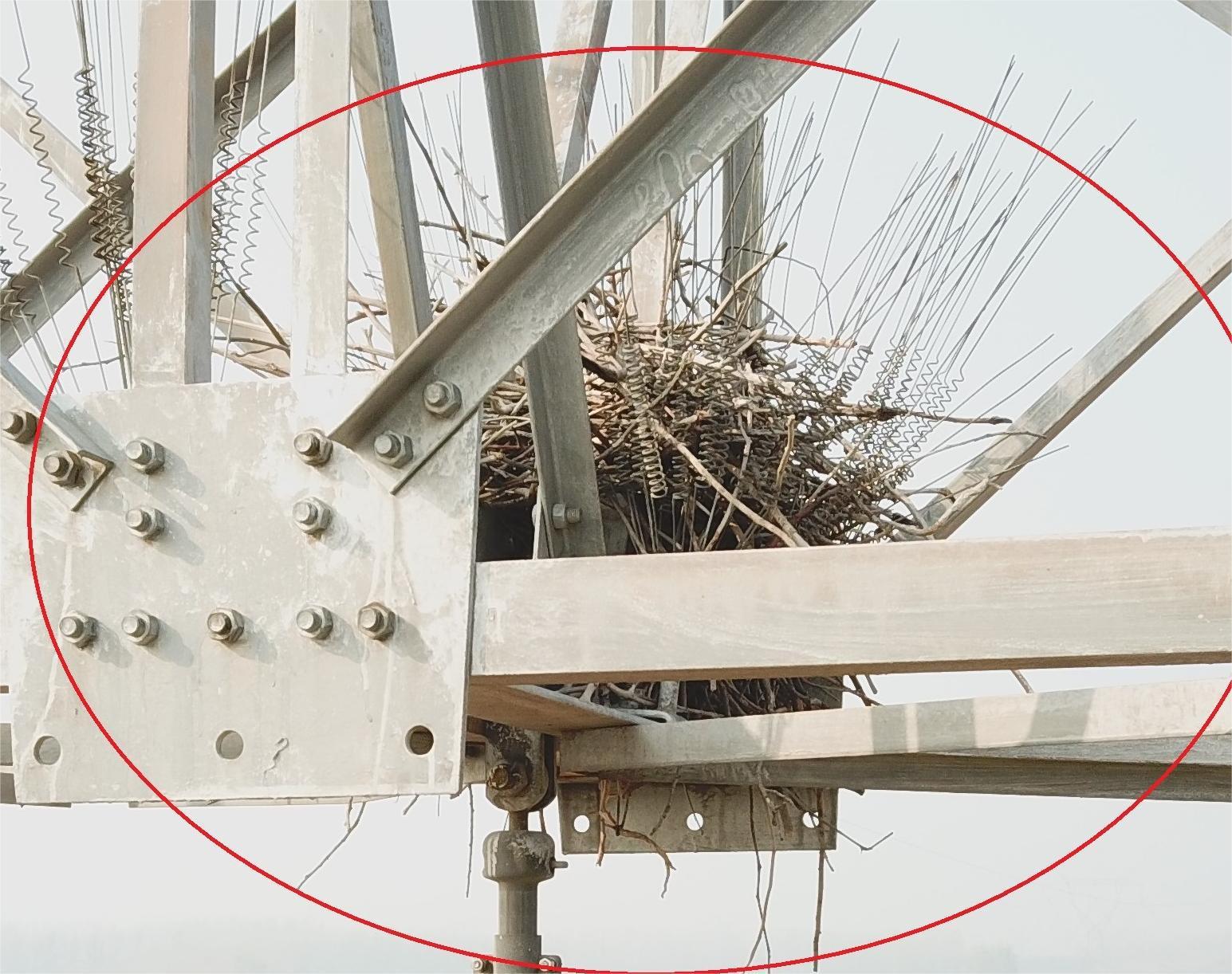

Supplement: Data S1 [file peerj-cs-10-2383-s001.zip › JPEGImages/0tbjXqQ3vdgk61uwRFY5lfiLZN9xI8BphEOeDKSG.jpg]

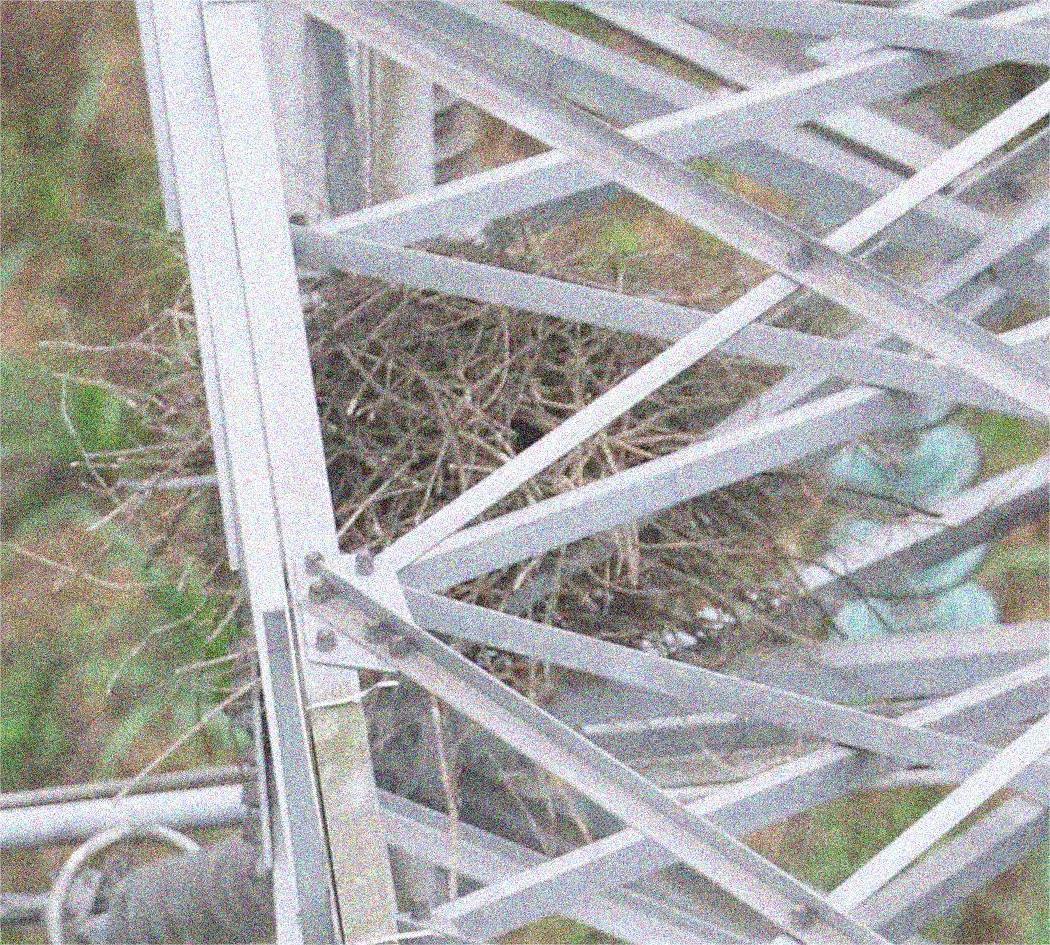

Supplement: Data S1 [file peerj-cs-10-2383-s001.zip › JPEGImages/0uPNH67ezwYcWC2VfXQOSh8LUG4qmkoTEbytpgxB.jpg]

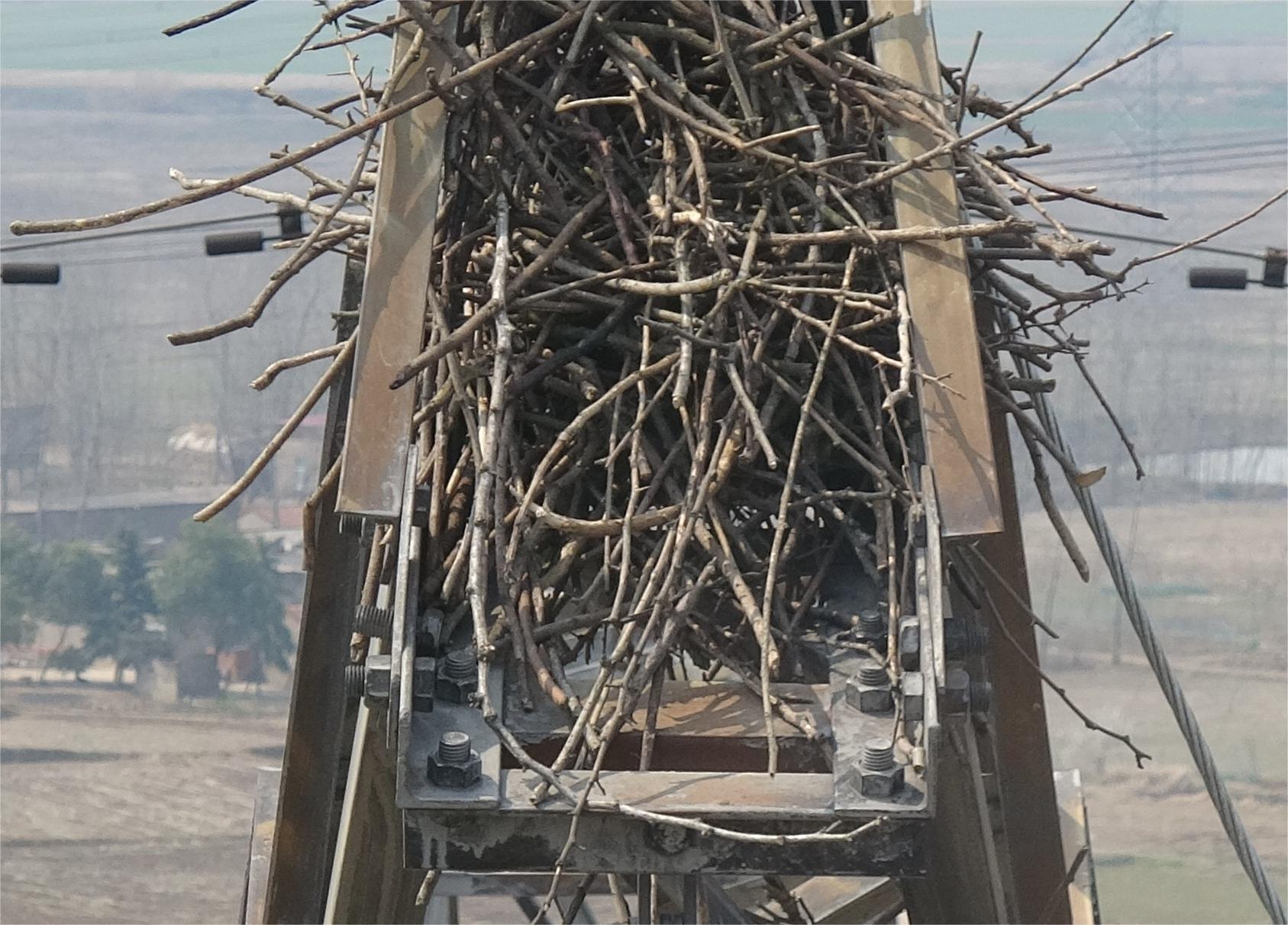

Supplement: Data S1 [file peerj-cs-10-2383-s001.zip › JPEGImages/1519784881134-ganta.jpg]

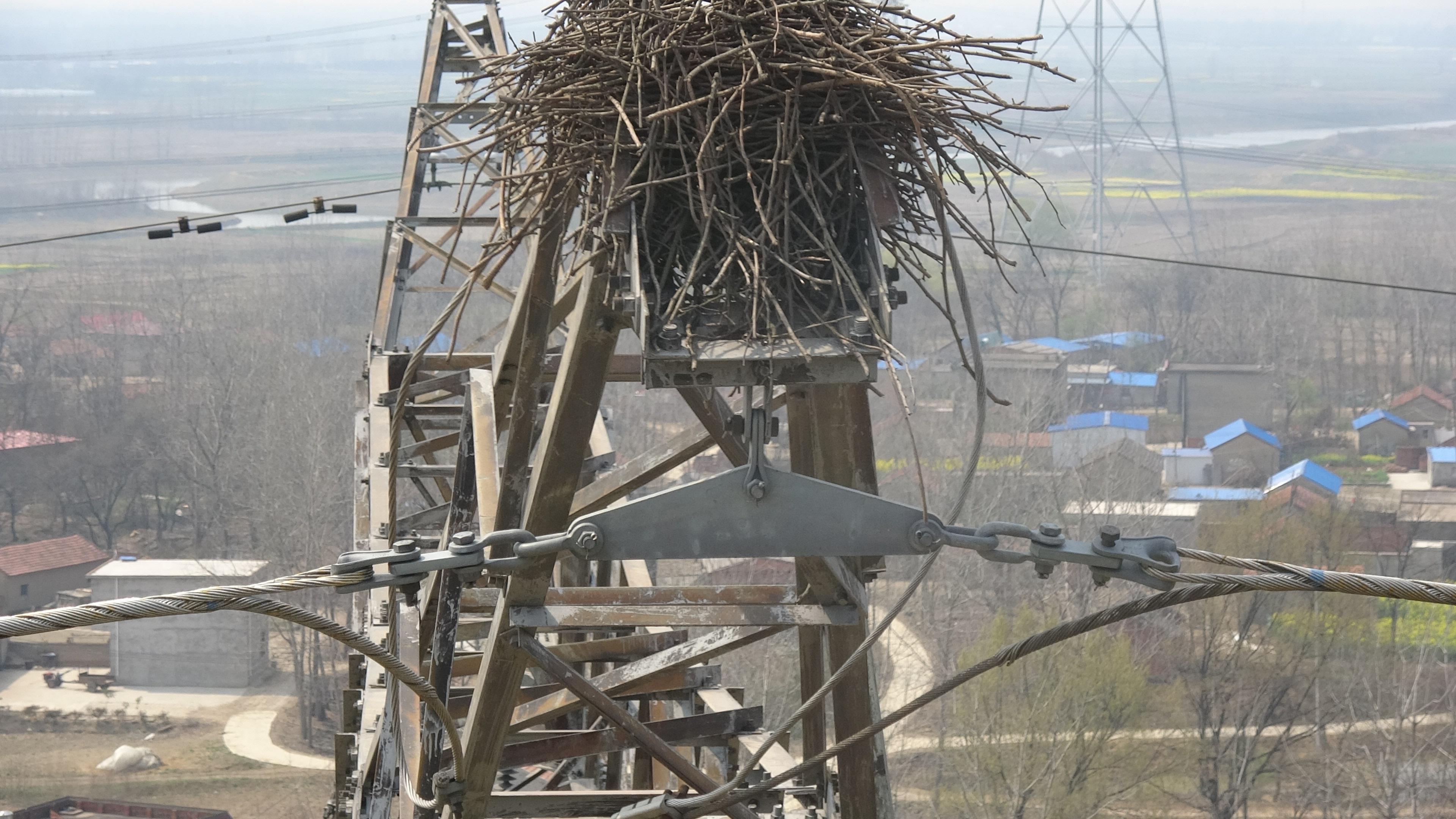

Supplement: Data S1 [file peerj-cs-10-2383-s001.zip › JPEGImages/1519784932111-ganta.jpg]

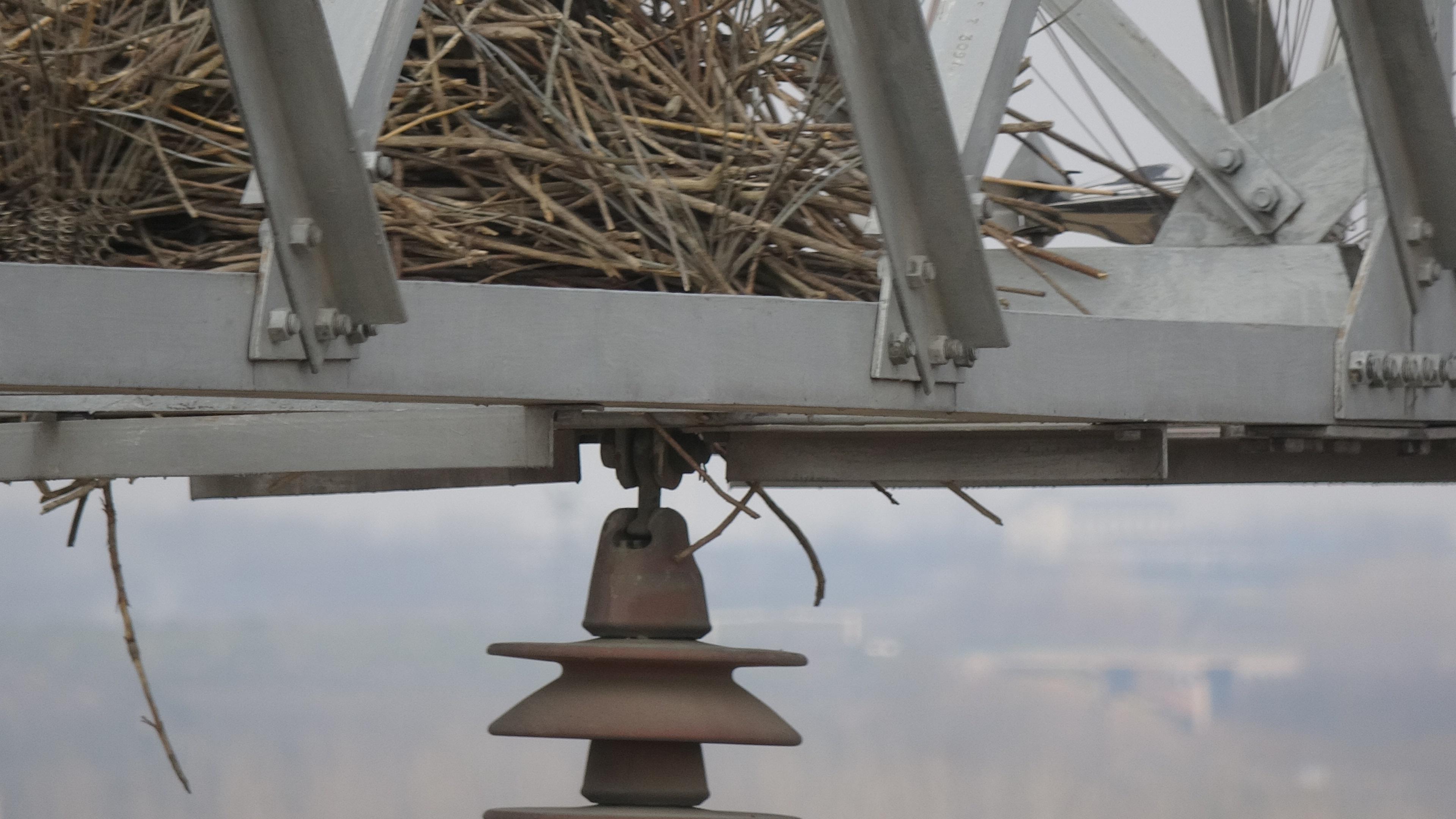

Supplement: Data S1 [file peerj-cs-10-2383-s001.zip › JPEGImages/1519785134350-ganta.jpg]

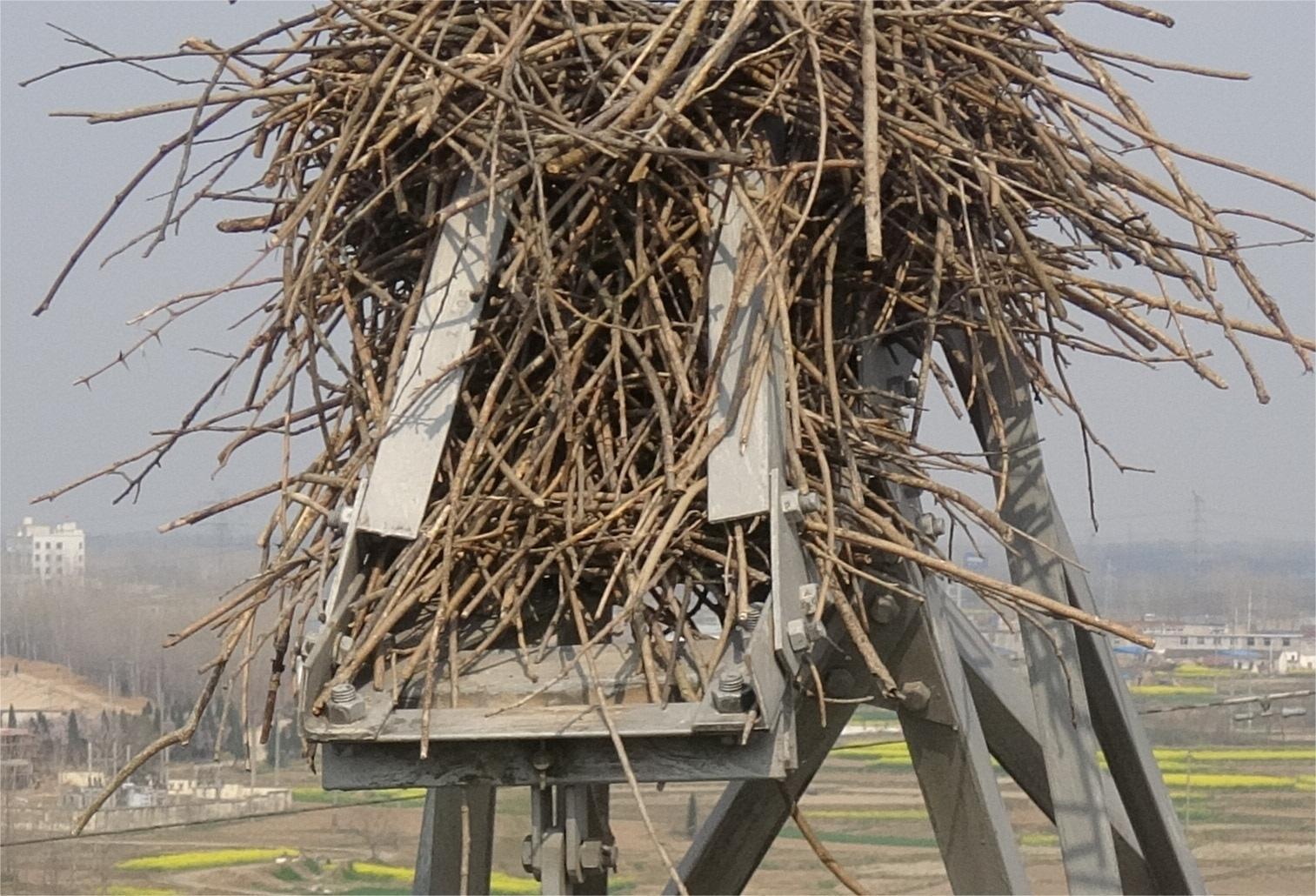

Supplement: Data S1 [file peerj-cs-10-2383-s001.zip › JPEGImages/1519785161606-ganta.jpg]

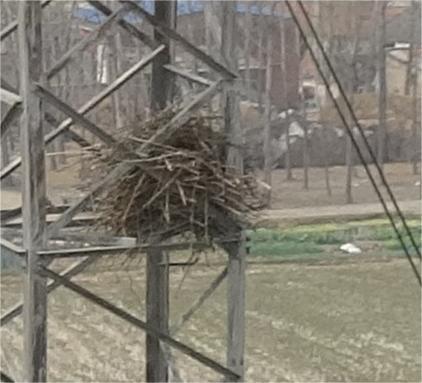

Supplement: Data S1 [file peerj-cs-10-2383-s001.zip › JPEGImages/1519785456687-ganta.jpg]

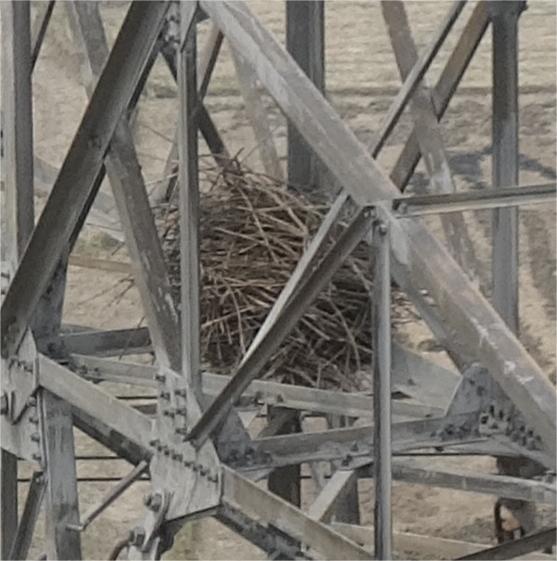

Supplement: Data S1 [file peerj-cs-10-2383-s001.zip › JPEGImages/1519785514026-ganta.jpg]

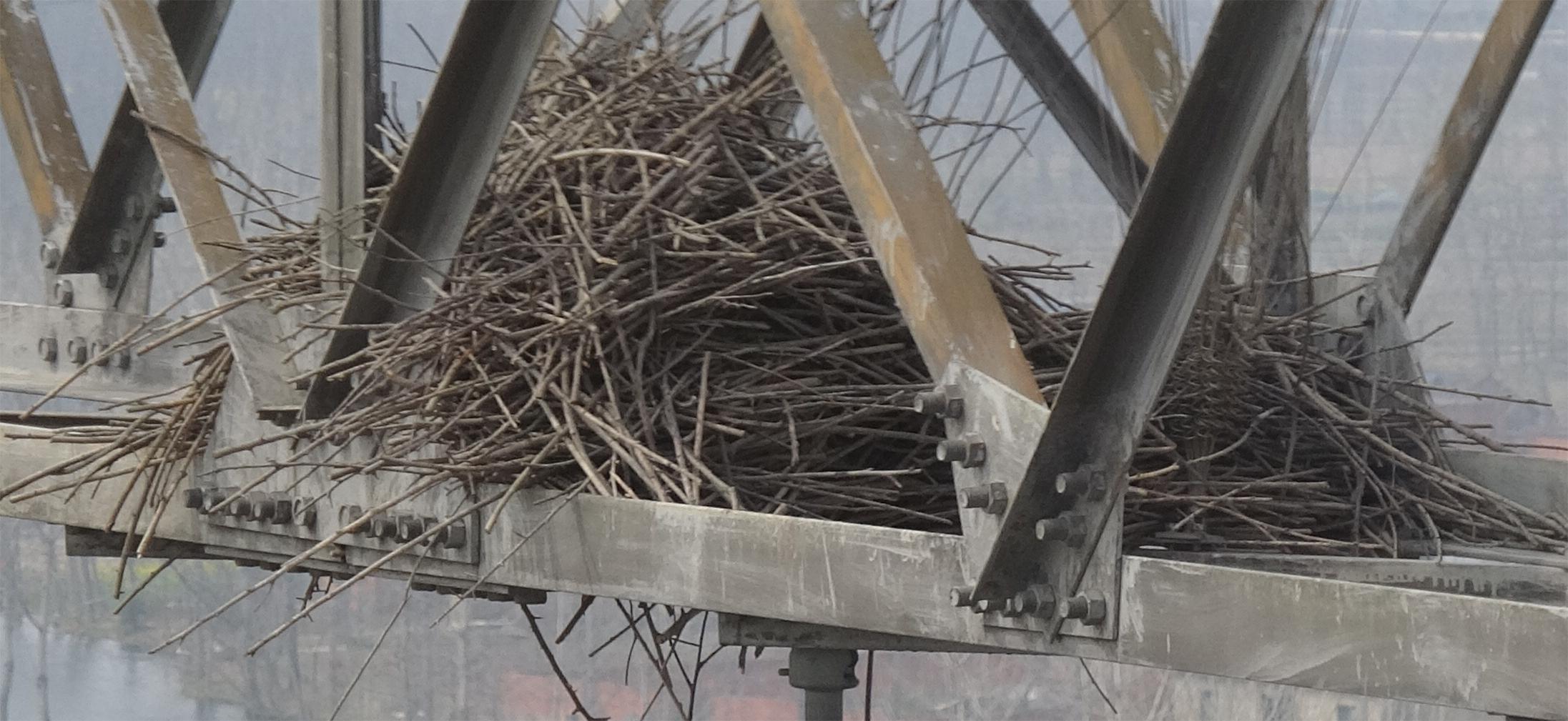

Supplement: Data S1 [file peerj-cs-10-2383-s001.zip › JPEGImages/1519785876905-ganta.jpg]

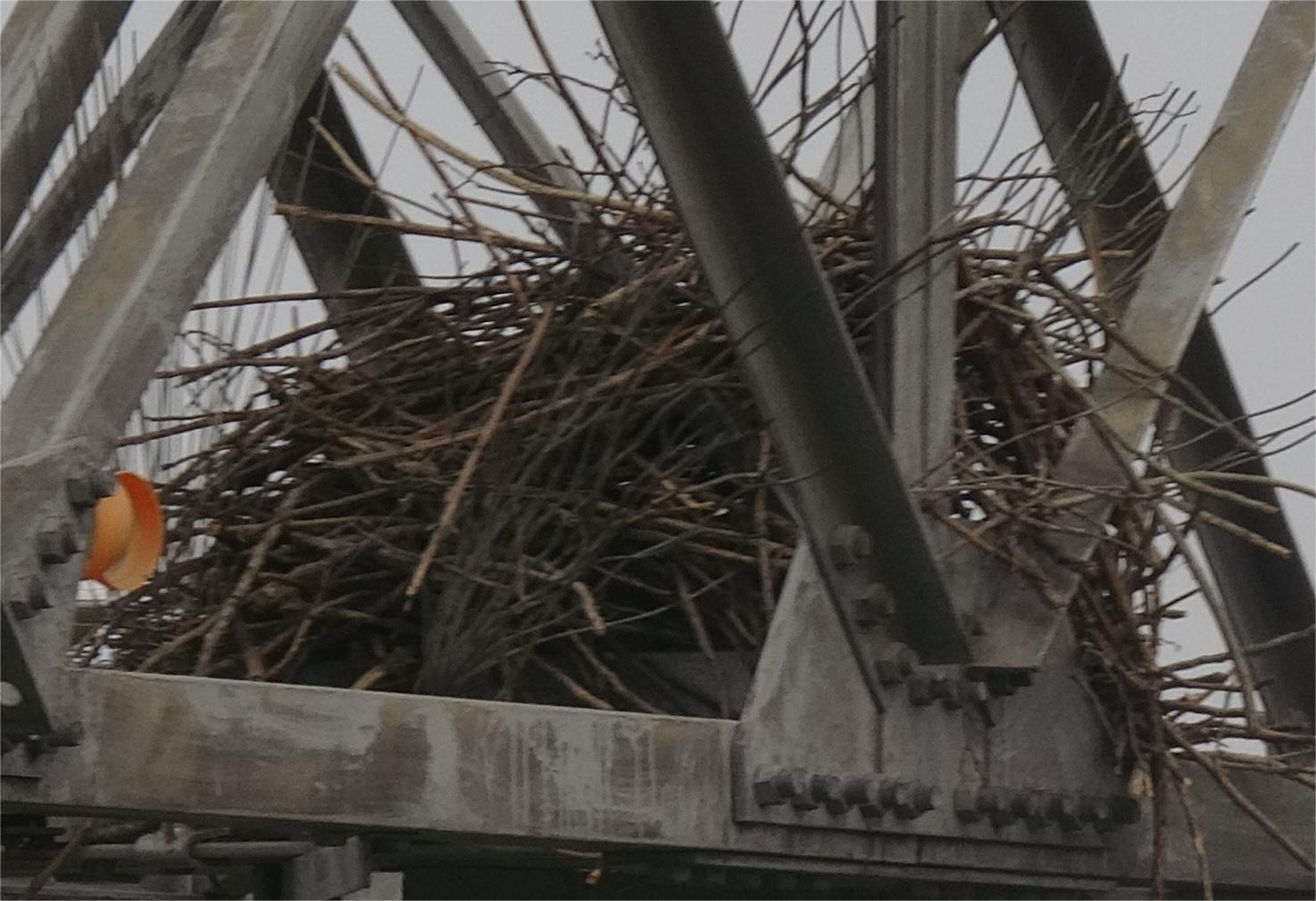

Supplement: Data S1 [file peerj-cs-10-2383-s001.zip › JPEGImages/1519785959889-ganta.jpg]

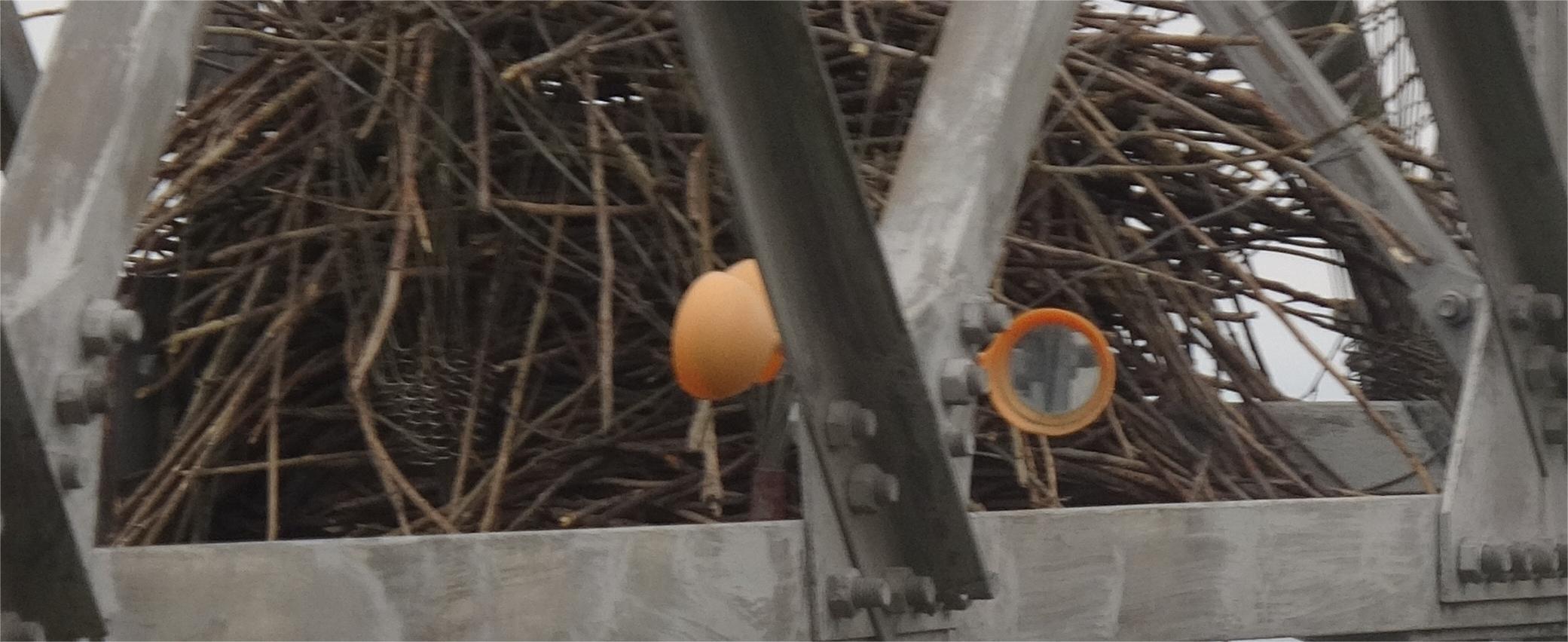

Supplement: Data S1 [file peerj-cs-10-2383-s001.zip › JPEGImages/1519786017582-ganta.jpg]

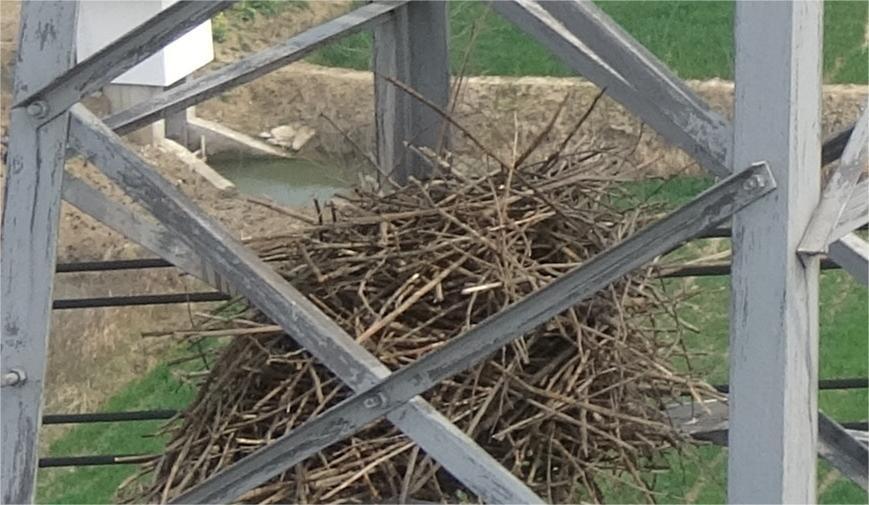

Supplement: Data S1 [file peerj-cs-10-2383-s001.zip › JPEGImages/1519786115161-ganta.jpg]

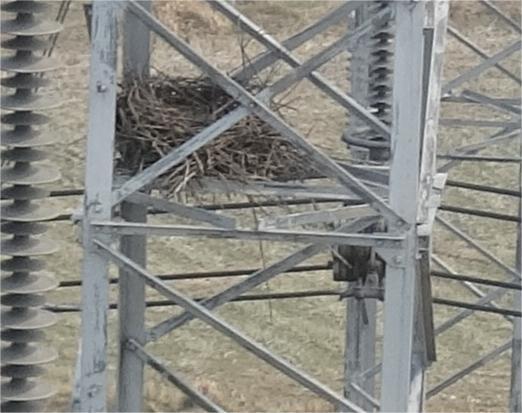

Supplement: Data S1 [file peerj-cs-10-2383-s001.zip › JPEGImages/1519786291739-ganta.jpg]

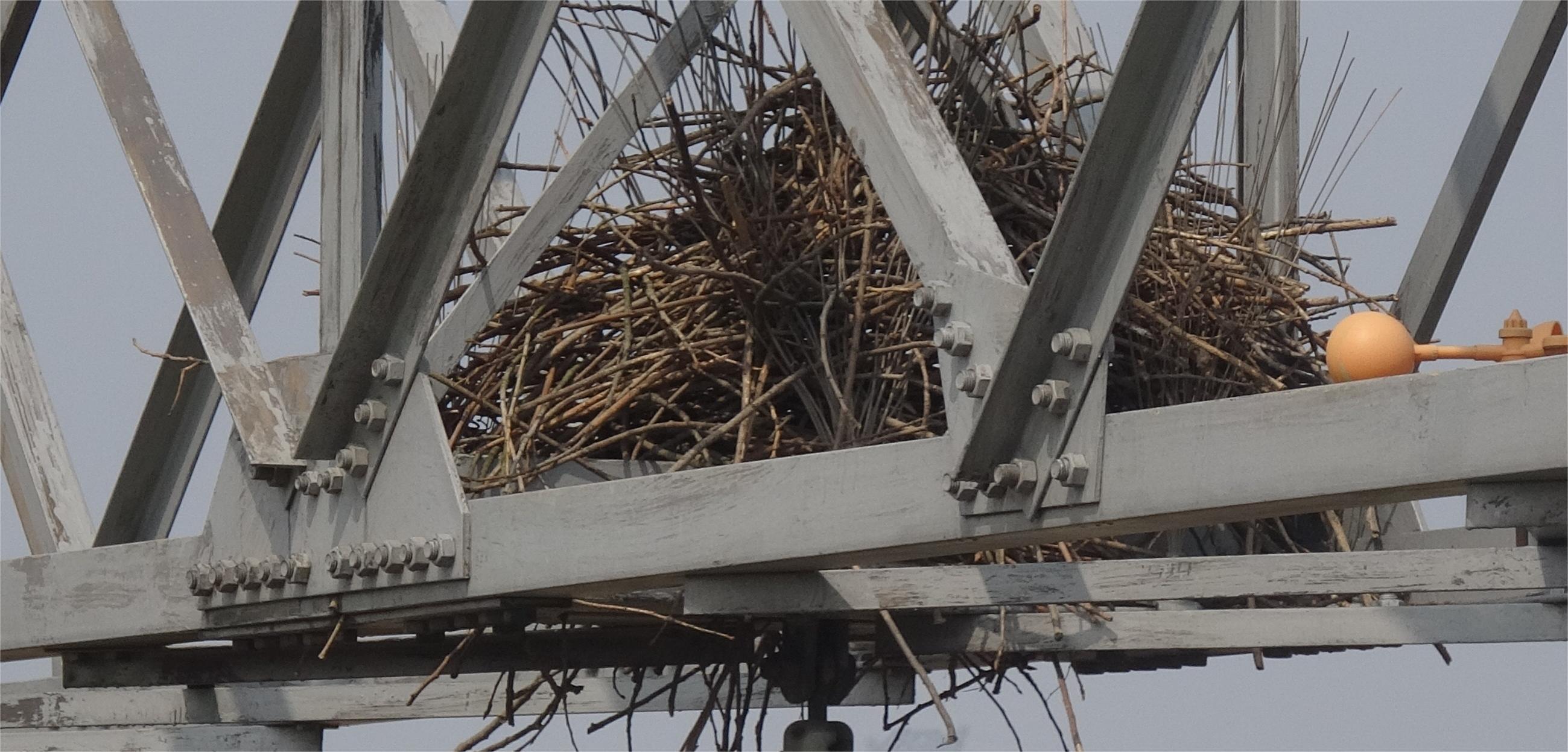

Supplement: Data S1 [file peerj-cs-10-2383-s001.zip › JPEGImages/1519786579887-ganta.jpg]

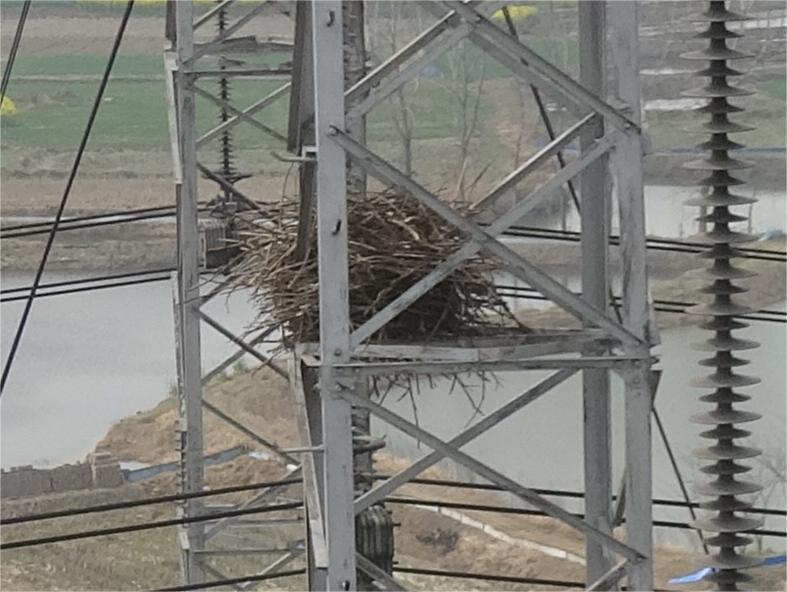

Supplement: Data S1 [file peerj-cs-10-2383-s001.zip › JPEGImages/1519786767753-ganta.jpg]

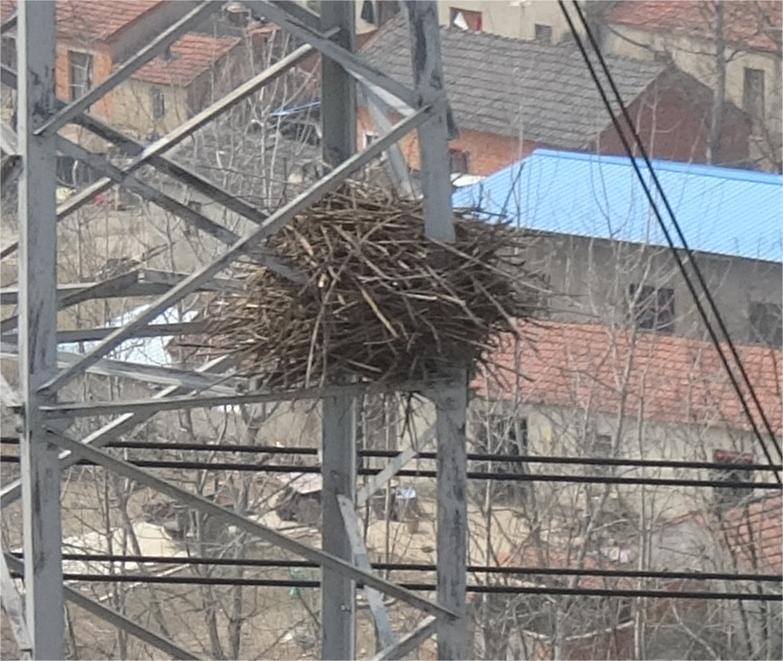

Supplement: Data S1 [file peerj-cs-10-2383-s001.zip › JPEGImages/1519786818596-ganta.jpg]

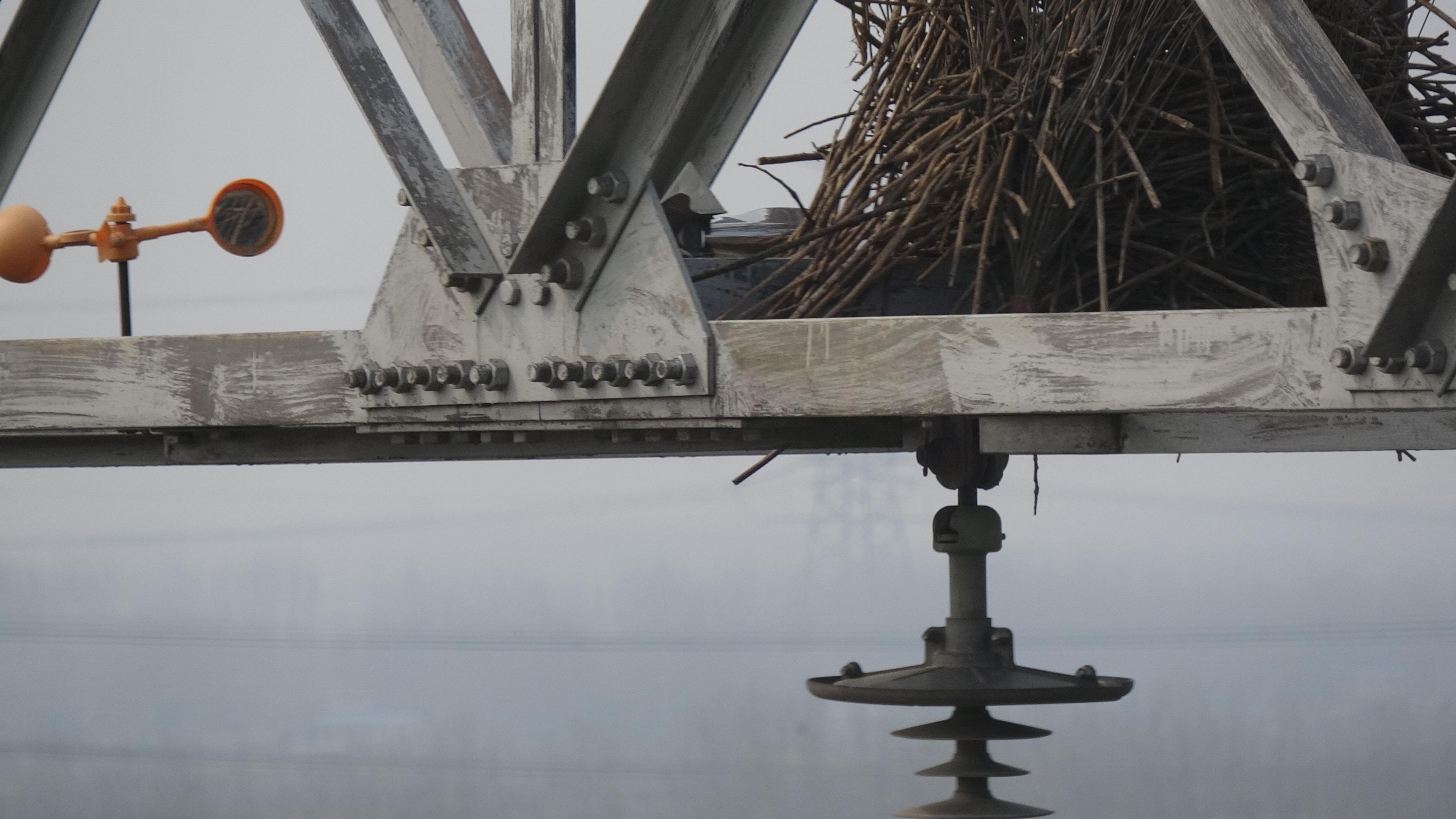

Supplement: Data S1 [file peerj-cs-10-2383-s001.zip › JPEGImages/1519787087798-ganta.jpg]

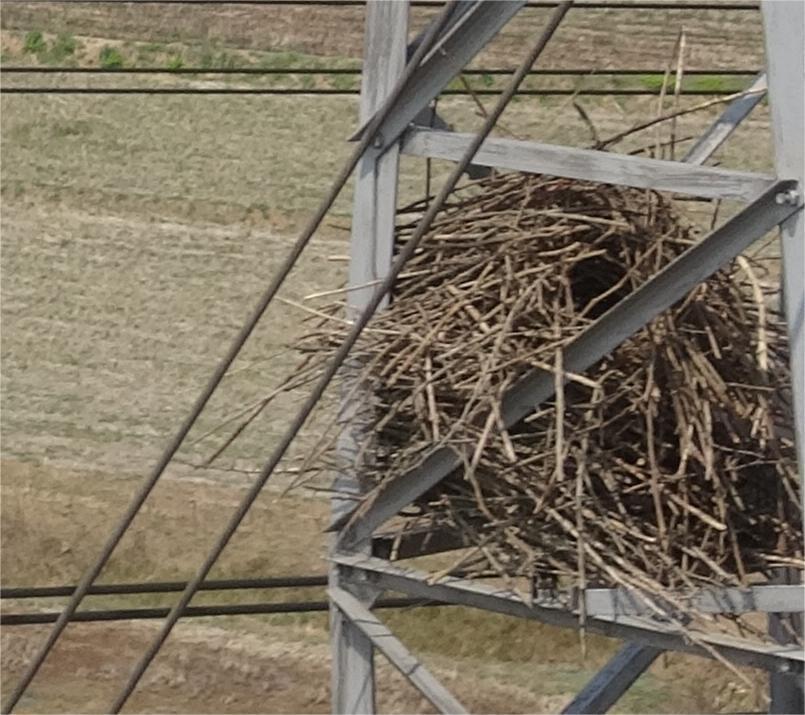

Supplement: Data S1 [file peerj-cs-10-2383-s001.zip › JPEGImages/1519787317733-ganta.jpg]

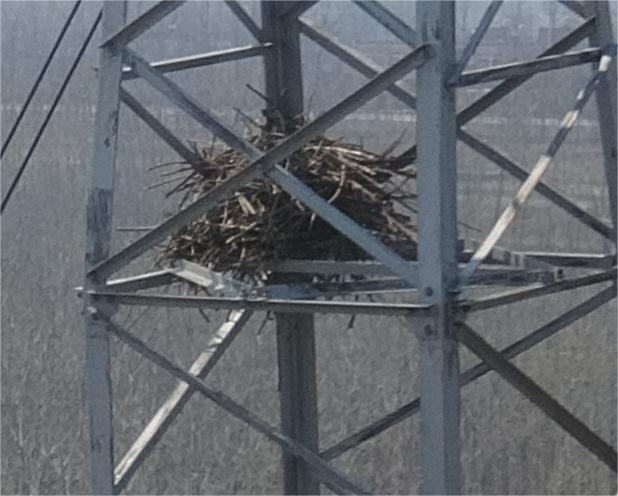

Supplement: Data S1 [file peerj-cs-10-2383-s001.zip › JPEGImages/1519787379928-ganta.jpg]

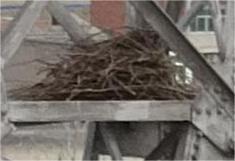

Supplement: Data S1 [file peerj-cs-10-2383-s001.zip › JPEGImages/1519787700201-ganta.jpg]

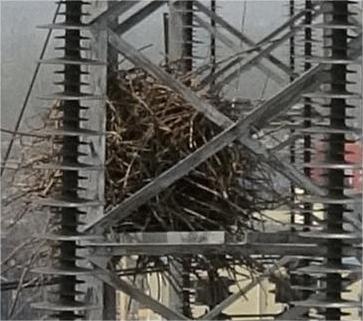

Supplement: Data S1 [file peerj-cs-10-2383-s001.zip › JPEGImages/1519787777845-ganta.jpg]

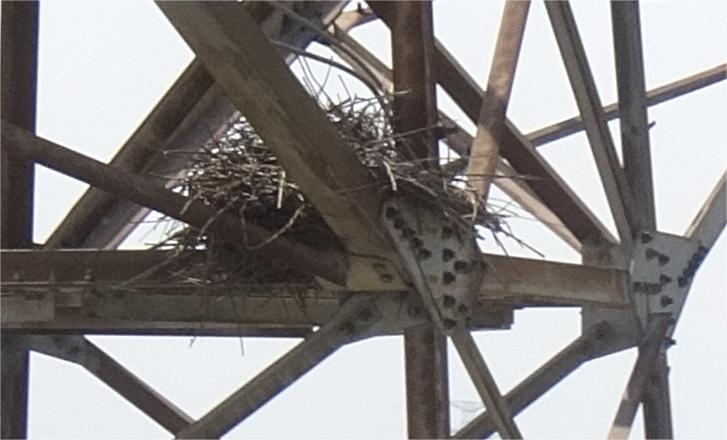

Supplement: Data S1 [file peerj-cs-10-2383-s001.zip › JPEGImages/1519787933676-ganta.jpg]

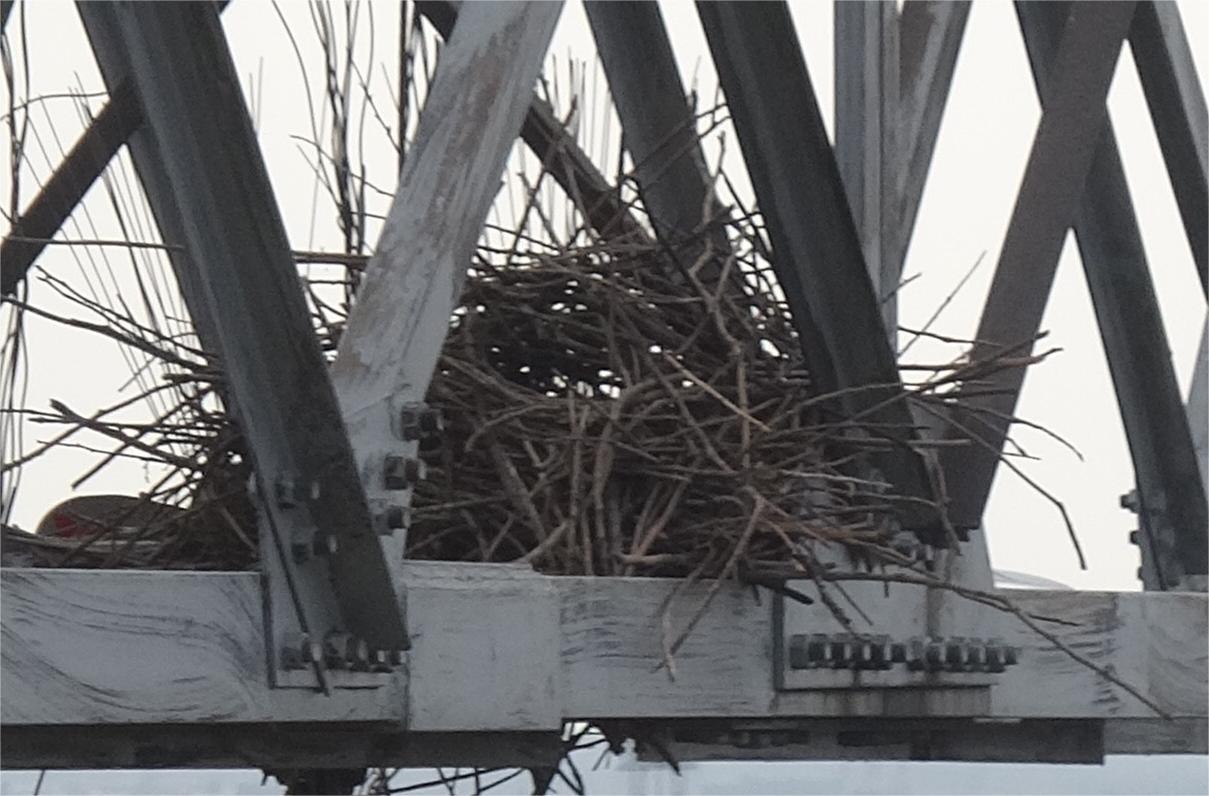

Supplement: Data S1 [file peerj-cs-10-2383-s001.zip › JPEGImages/1519788156220-ganta.jpg]

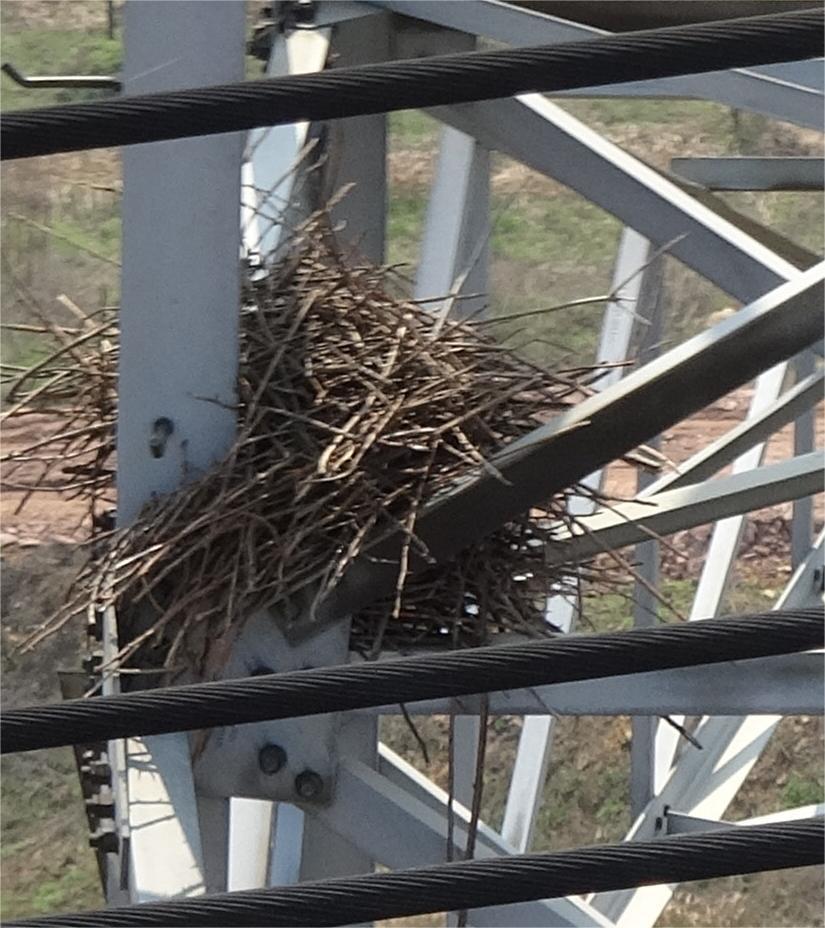

Supplement: Data S1 [file peerj-cs-10-2383-s001.zip › JPEGImages/1519797930447-ganta.jpg]

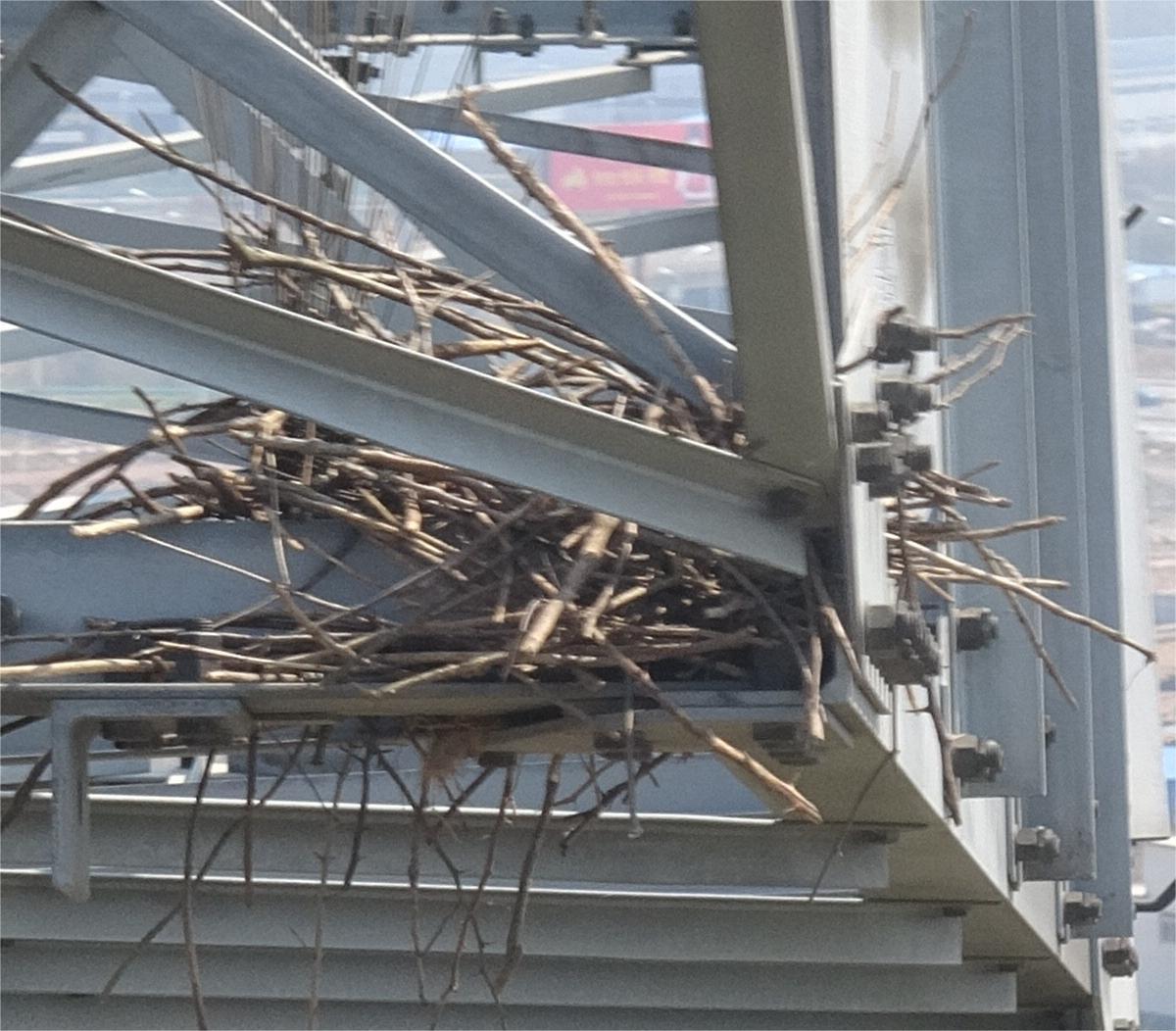

Supplement: Data S1 [file peerj-cs-10-2383-s001.zip › JPEGImages/1519798133671-ganta.jpg]

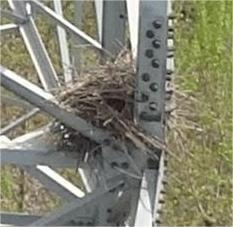

Supplement: Data S1 [file peerj-cs-10-2383-s001.zip › JPEGImages/1519798629619-ganta.jpg]

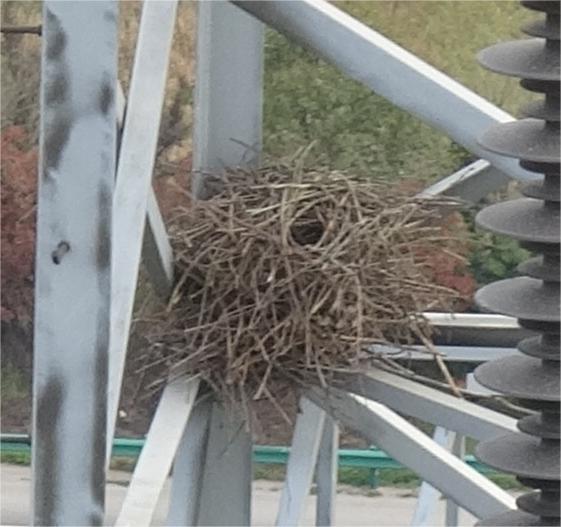

Supplement: Data S1 [file peerj-cs-10-2383-s001.zip › JPEGImages/1519798667034-ganta.jpg]

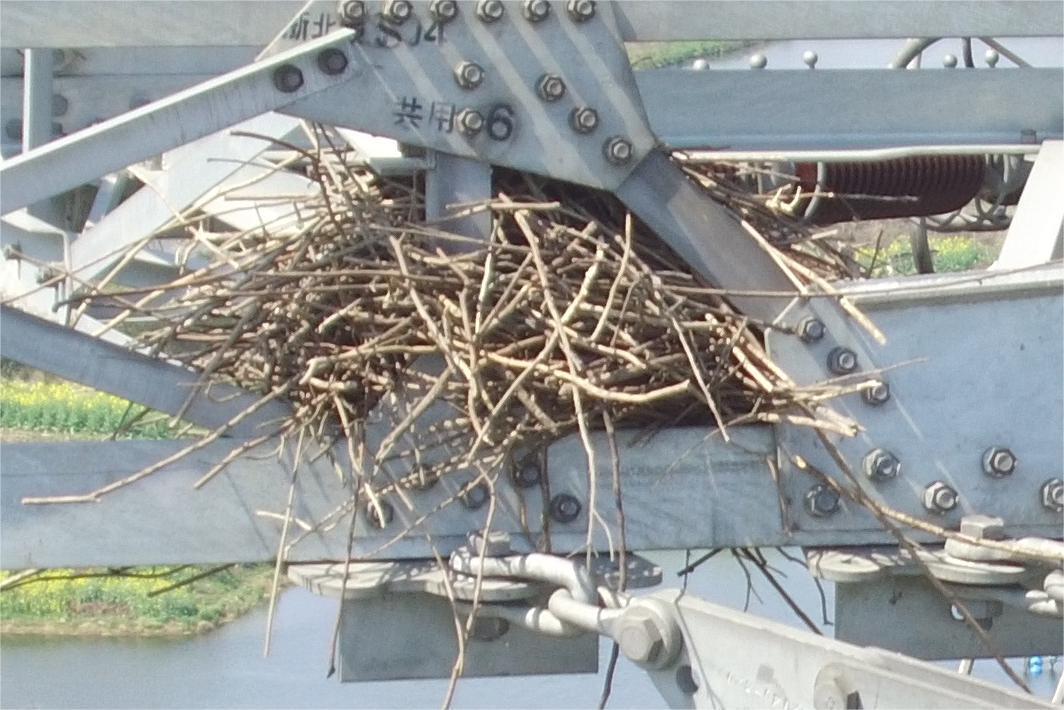

Supplement: Data S1 [file peerj-cs-10-2383-s001.zip › JPEGImages/1519798915324-ganta.jpg]

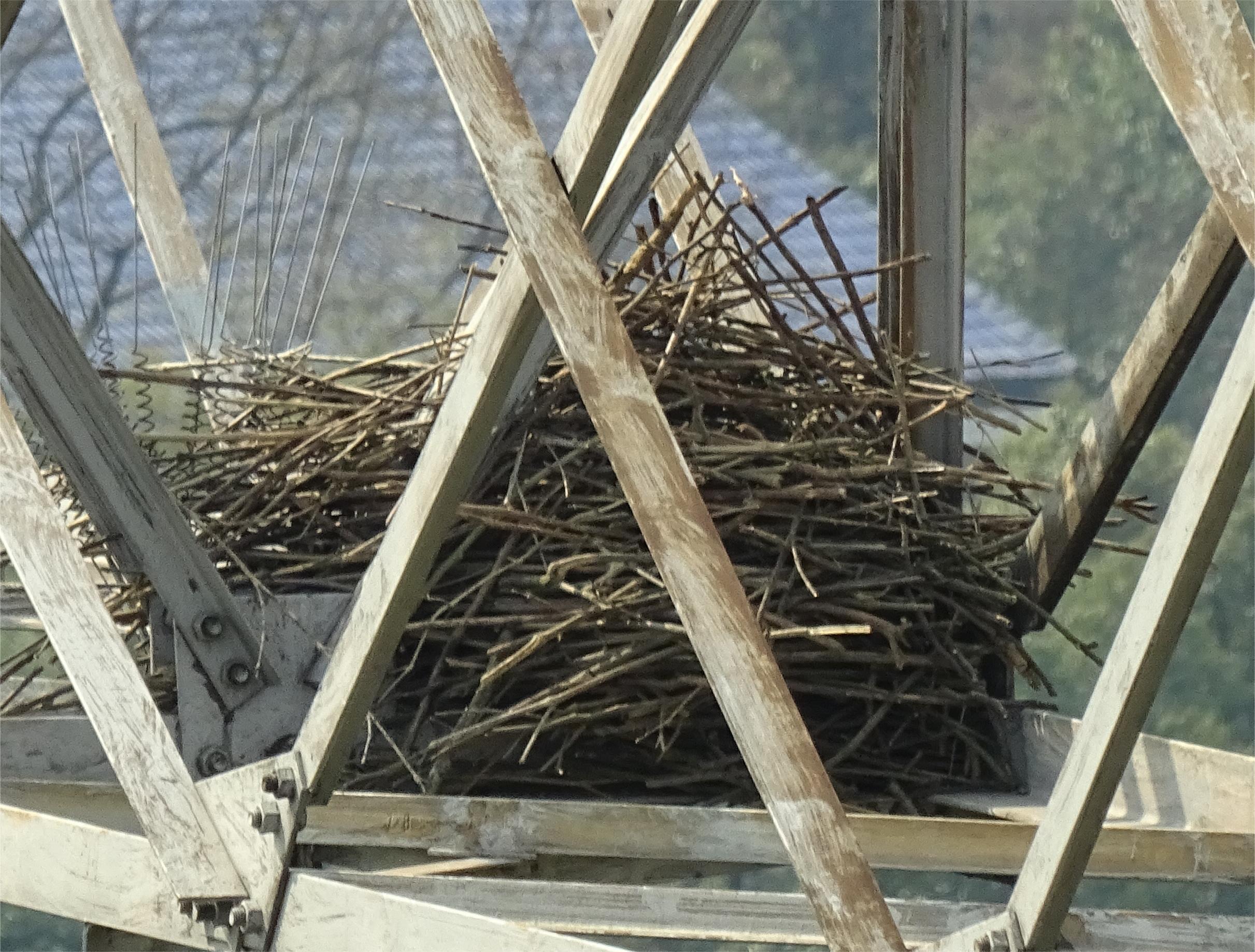

Supplement: Data S1 [file peerj-cs-10-2383-s001.zip › JPEGImages/1519799059056-ganta.jpg]

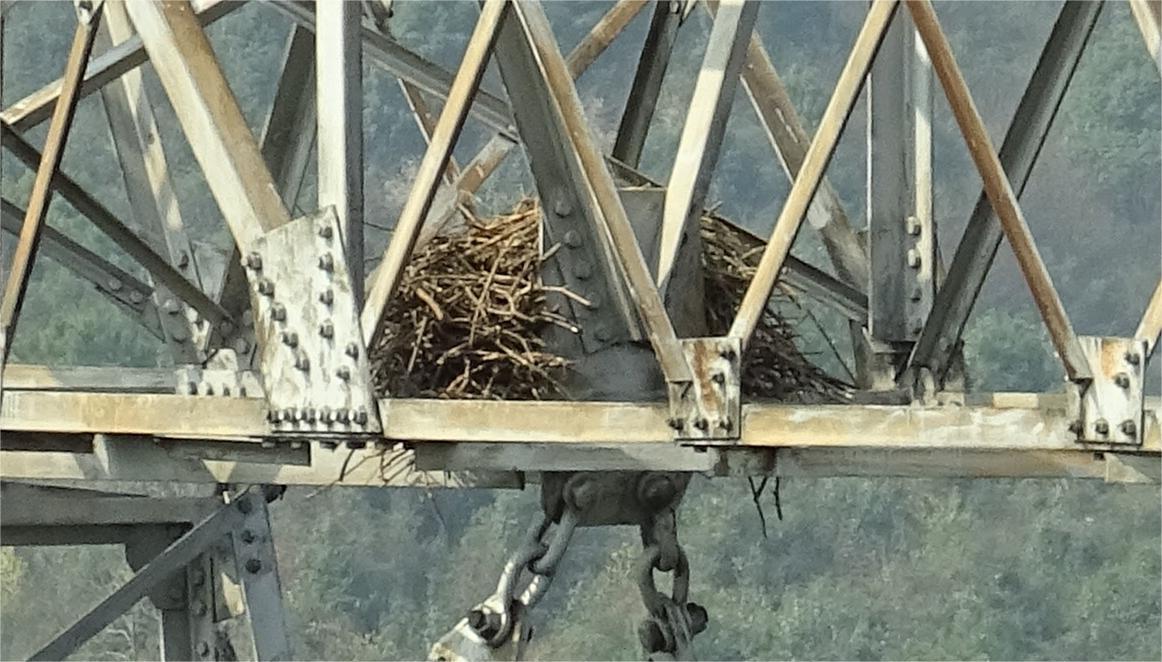

Supplement: Data S1 [file peerj-cs-10-2383-s001.zip › JPEGImages/1519799113836-ganta.jpg]

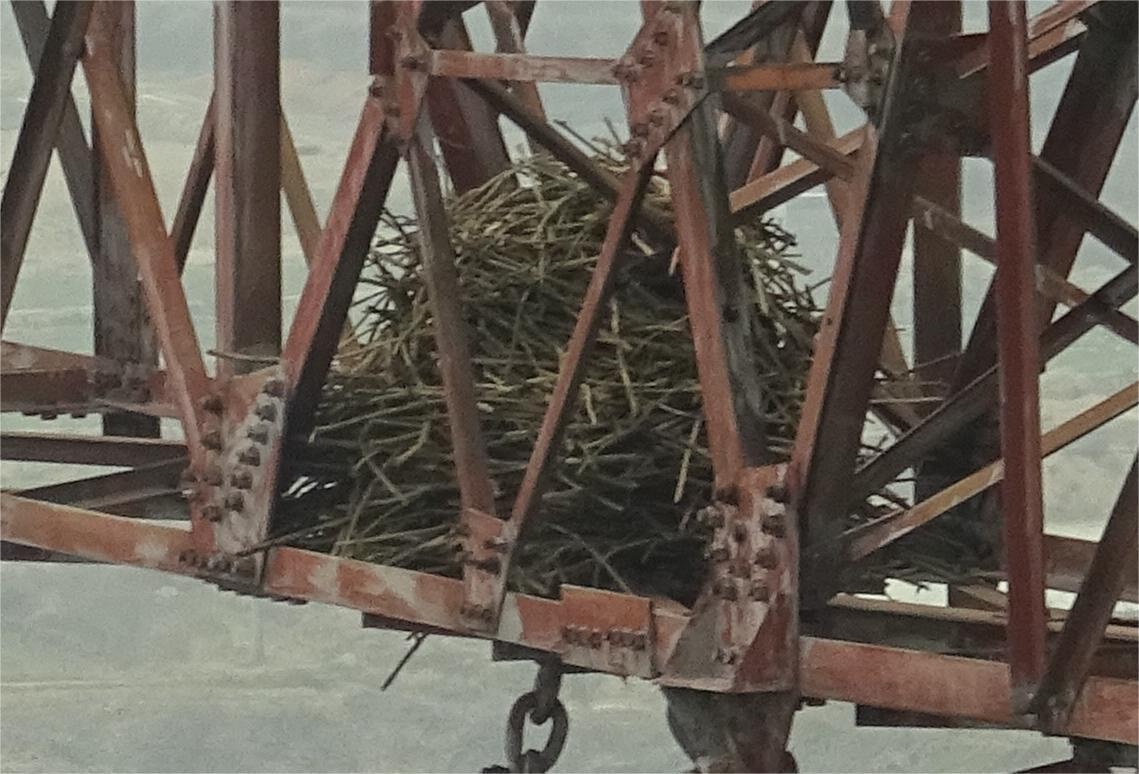

Supplement: Data S1 [file peerj-cs-10-2383-s001.zip › JPEGImages/1519799230446-ganta.jpg]

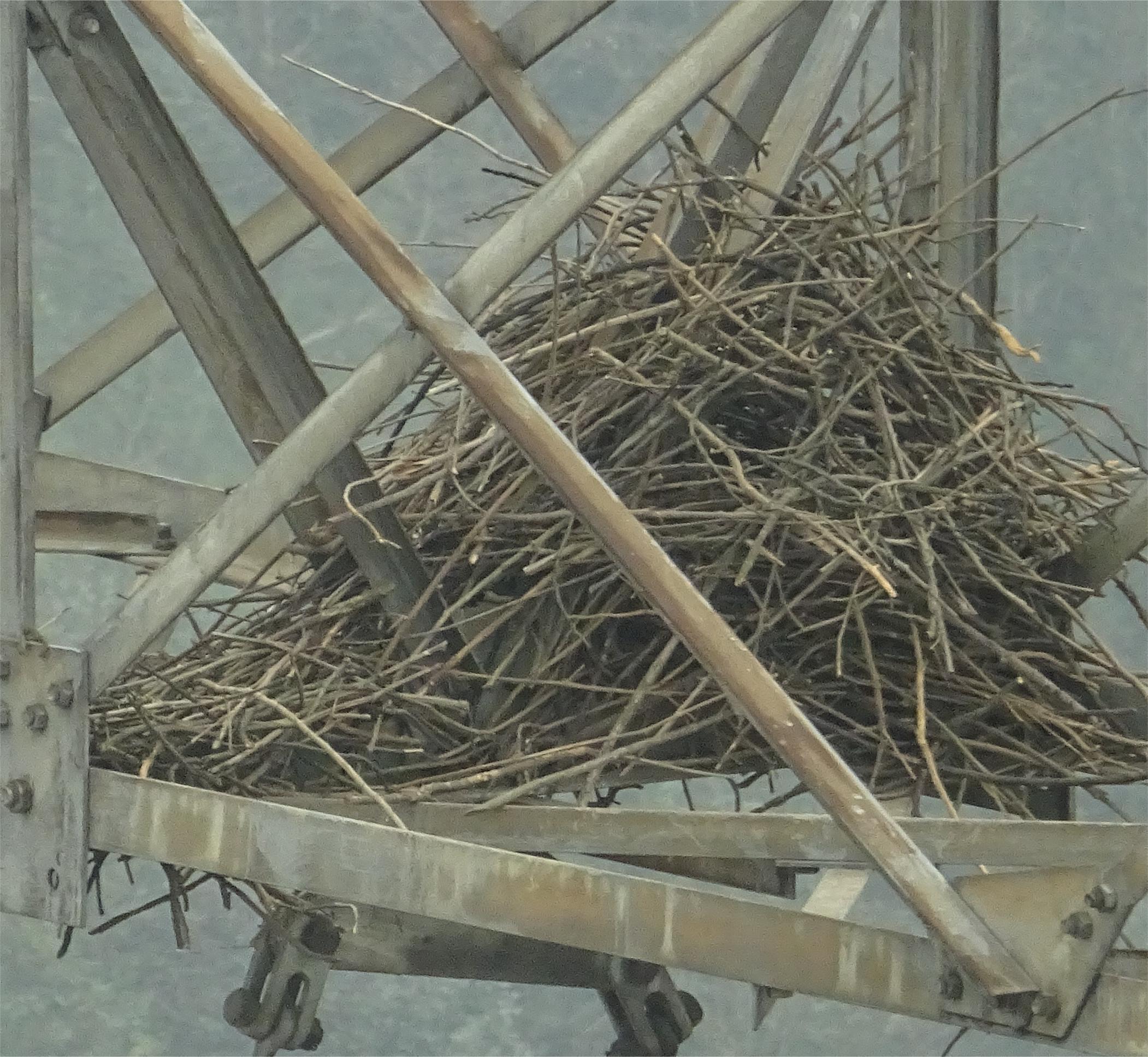

Supplement: Data S1 [file peerj-cs-10-2383-s001.zip › JPEGImages/1519799311060-ganta.jpg]

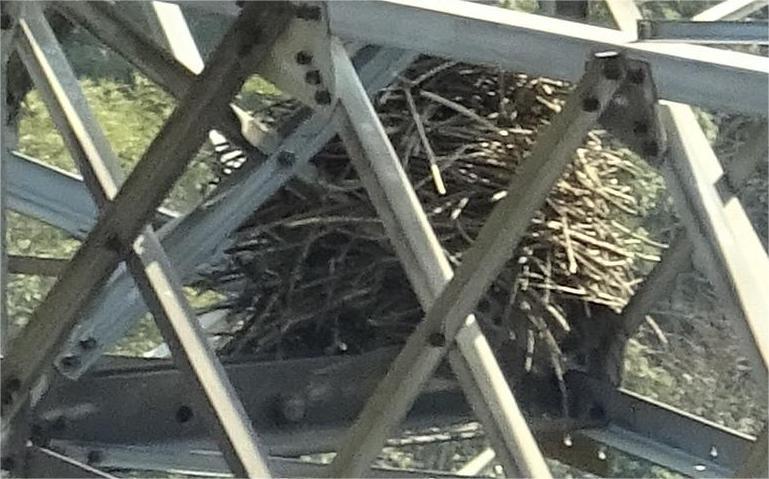

Supplement: Data S1 [file peerj-cs-10-2383-s001.zip › JPEGImages/1519799448624-ganta.jpg]

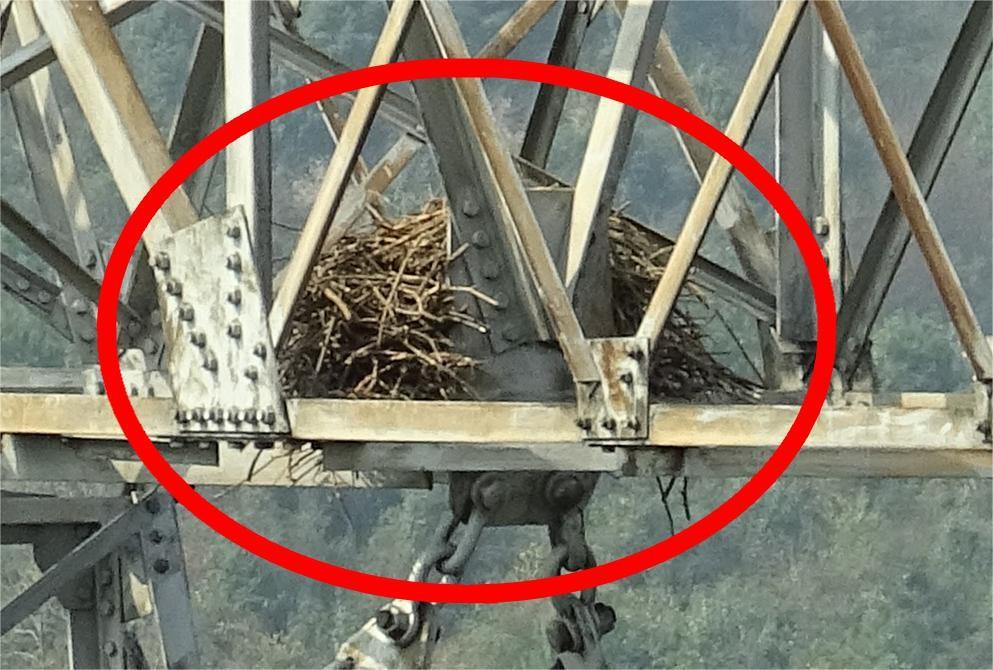

Supplement: Data S1 [file peerj-cs-10-2383-s001.zip › JPEGImages/1519799568049-ganta.jpg]

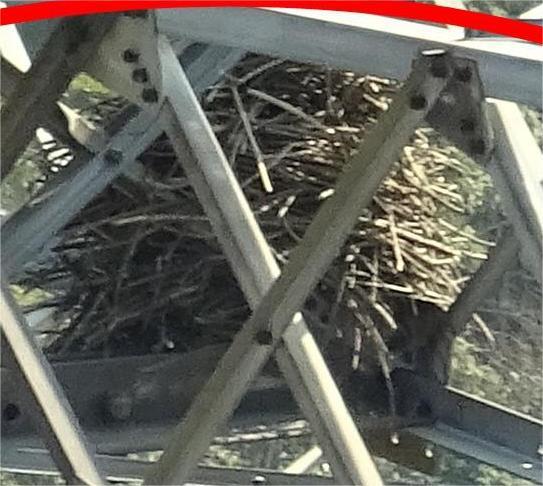

Supplement: Data S1 [file peerj-cs-10-2383-s001.zip › JPEGImages/1519799905756-ganta.jpg]

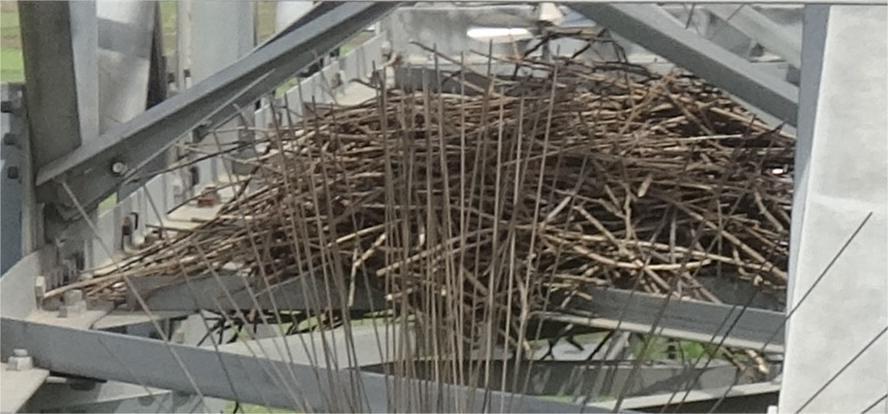

Supplement: Data S1 [file peerj-cs-10-2383-s001.zip › JPEGImages/1519800213614-ganta.jpg]

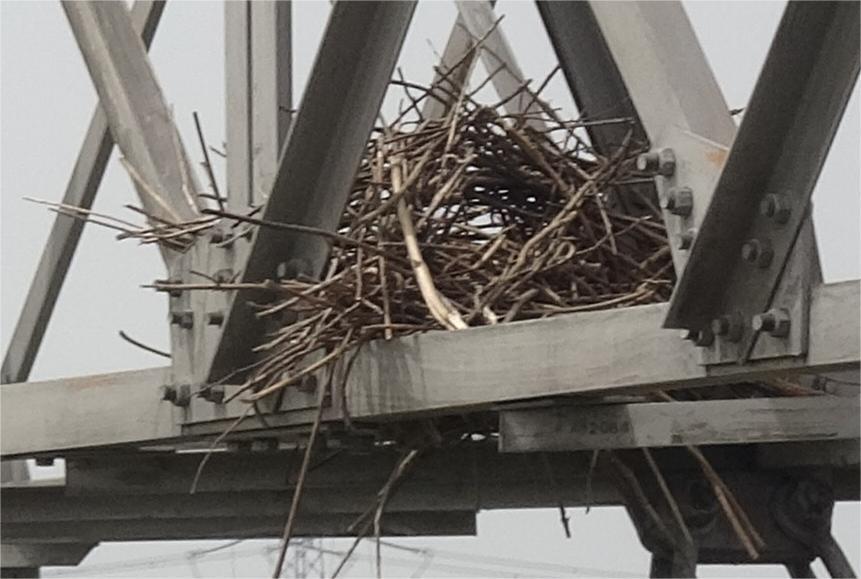

Supplement: Data S1 [file peerj-cs-10-2383-s001.zip › JPEGImages/1519800698717-ganta.jpg]

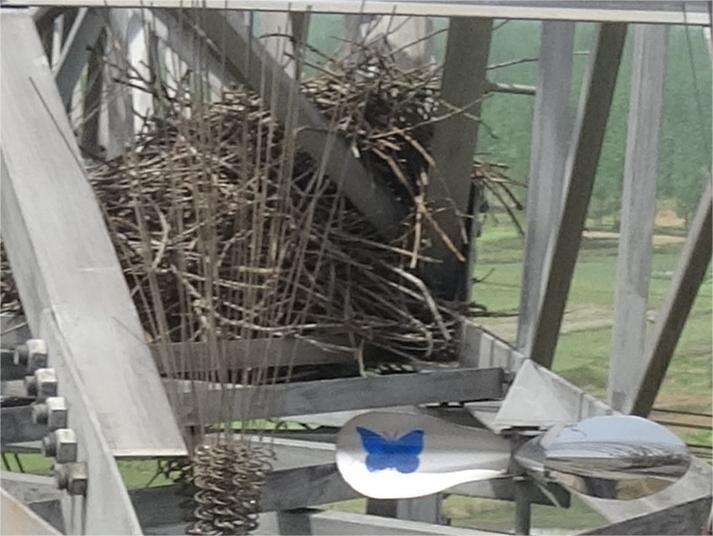

Supplement: Data S1 [file peerj-cs-10-2383-s001.zip › JPEGImages/1519800740886-ganta.jpg]

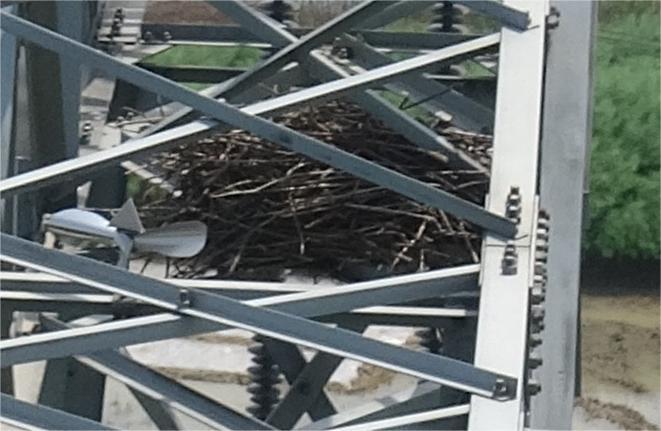

Supplement: Data S1 [file peerj-cs-10-2383-s001.zip › JPEGImages/1519800817852-ganta.jpg]

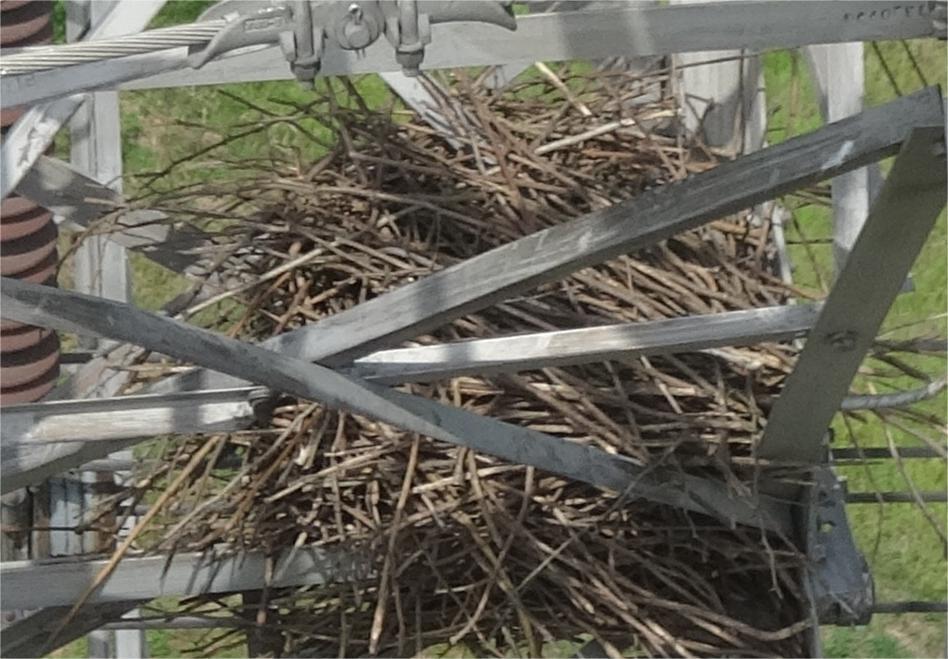

Supplement: Data S1 [file peerj-cs-10-2383-s001.zip › JPEGImages/1519800957386-ganta.jpg]

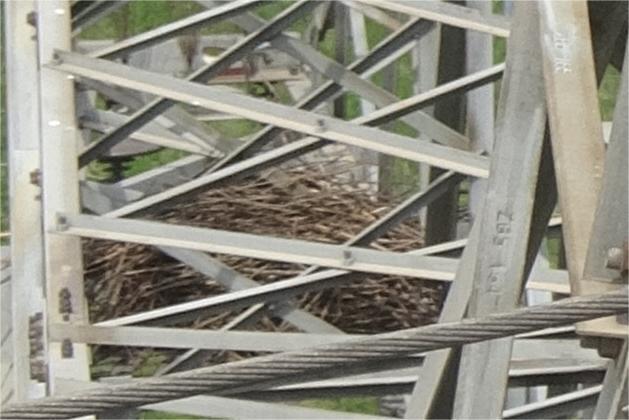

Supplement: Data S1 [file peerj-cs-10-2383-s001.zip › JPEGImages/1519801185349-ganta.jpg]

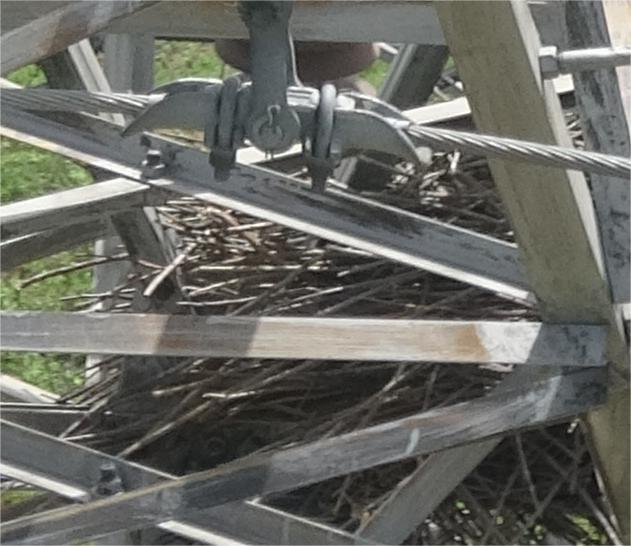

Supplement: Data S1 [file peerj-cs-10-2383-s001.zip › JPEGImages/1519801328968-ganta.jpg]

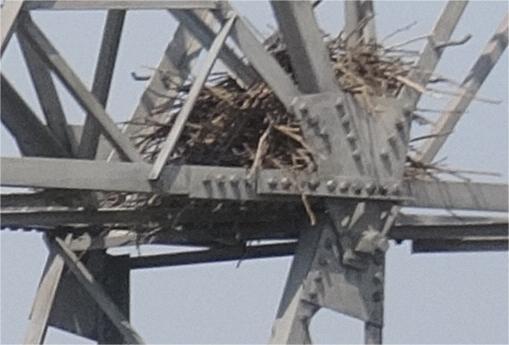

Supplement: Data S1 [file peerj-cs-10-2383-s001.zip › JPEGImages/1519801490698-ganta.jpg]

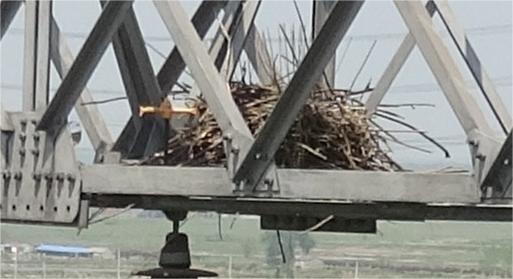

Supplement: Data S1 [file peerj-cs-10-2383-s001.zip › JPEGImages/1519801941025-ganta.jpg]

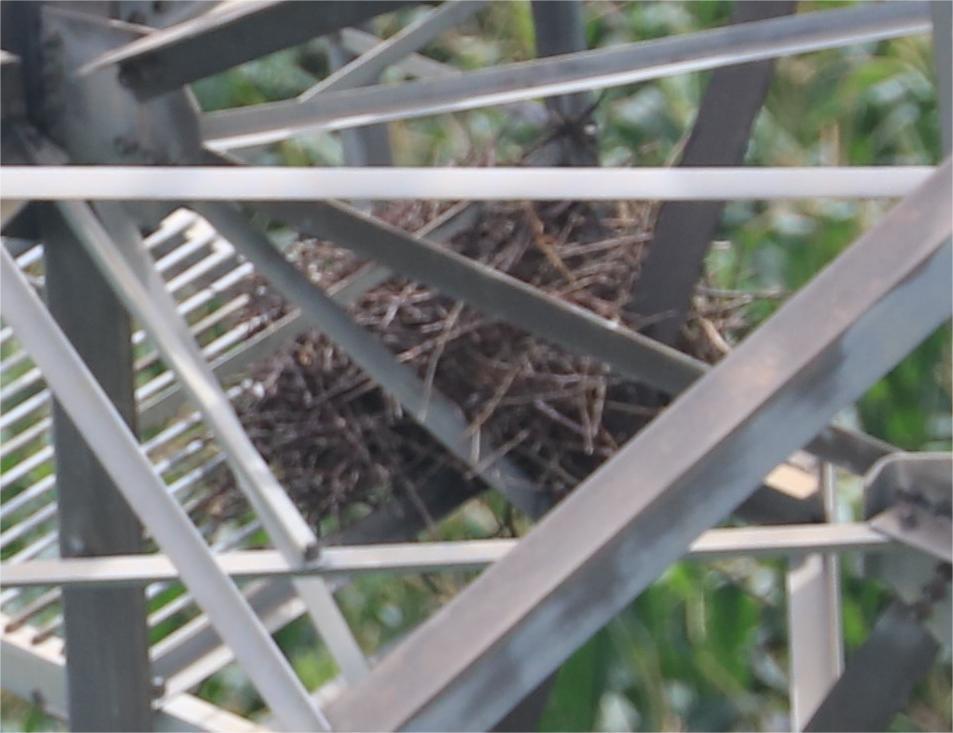

Supplement: Data S1 [file peerj-cs-10-2383-s001.zip › JPEGImages/1EWkXOa7i6Rl9BFKvzdxZLrUjngtmMPuGcoA8b45.jpg]

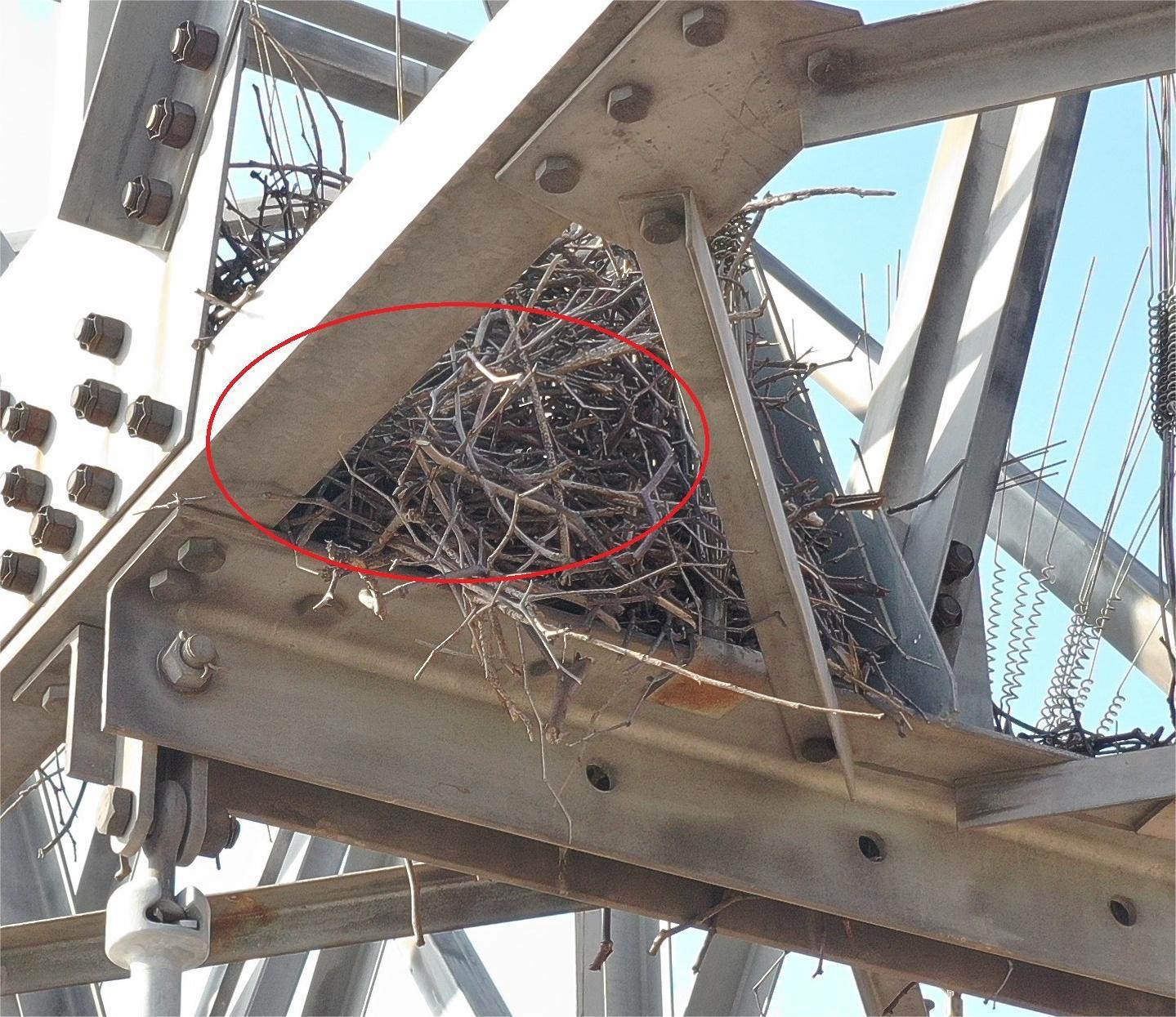

Supplement: Data S1 [file peerj-cs-10-2383-s001.zip › JPEGImages/1eCIEB6Q2YF3UTmftLsng7ziabPvdKN5VhSoHGyx.jpg]

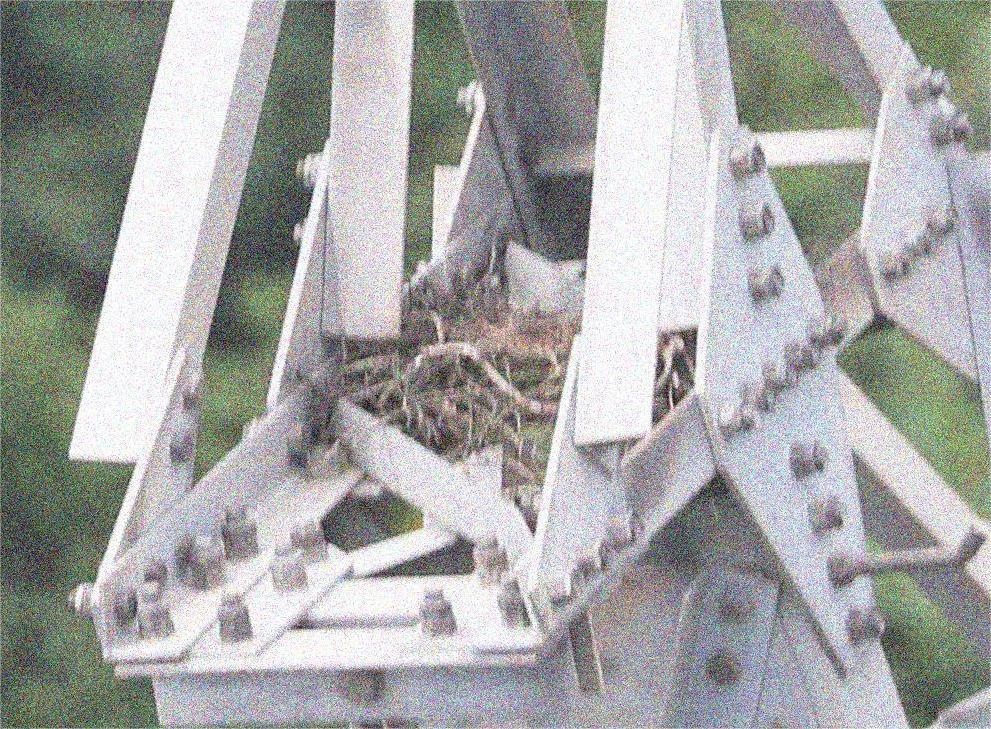

Supplement: Data S1 [file peerj-cs-10-2383-s001.zip › JPEGImages/1gsaO3CFH0VeNmLRzvQf5IPbpEWDnyTG2Y679uSd.jpg]

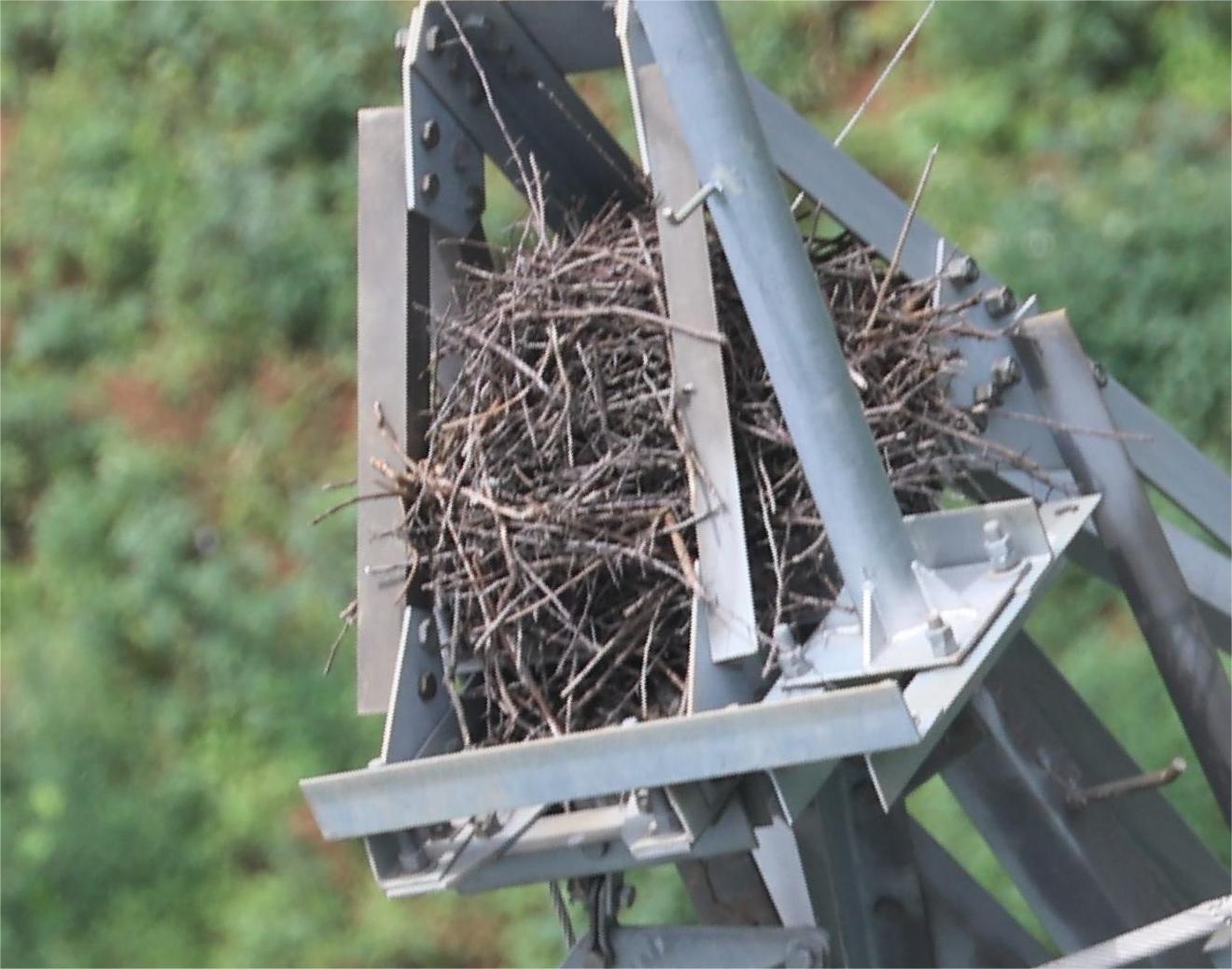

Supplement: Data S1 [file peerj-cs-10-2383-s001.zip › JPEGImages/1mPwtlFCOoigN5fIRjxZ0z3qupnQ2ave9GLDWbsB.jpg]

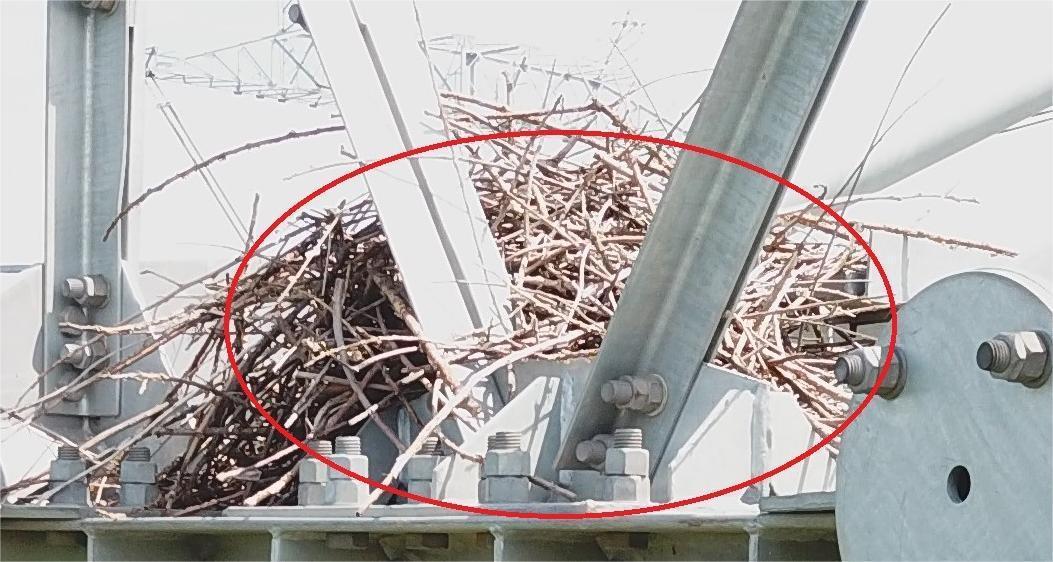

Supplement: Data S1 [file peerj-cs-10-2383-s001.zip › JPEGImages/1rSyC4Unlt8BseAmqz56D3IWPYJQVd7uaFgjfb2R.jpg]

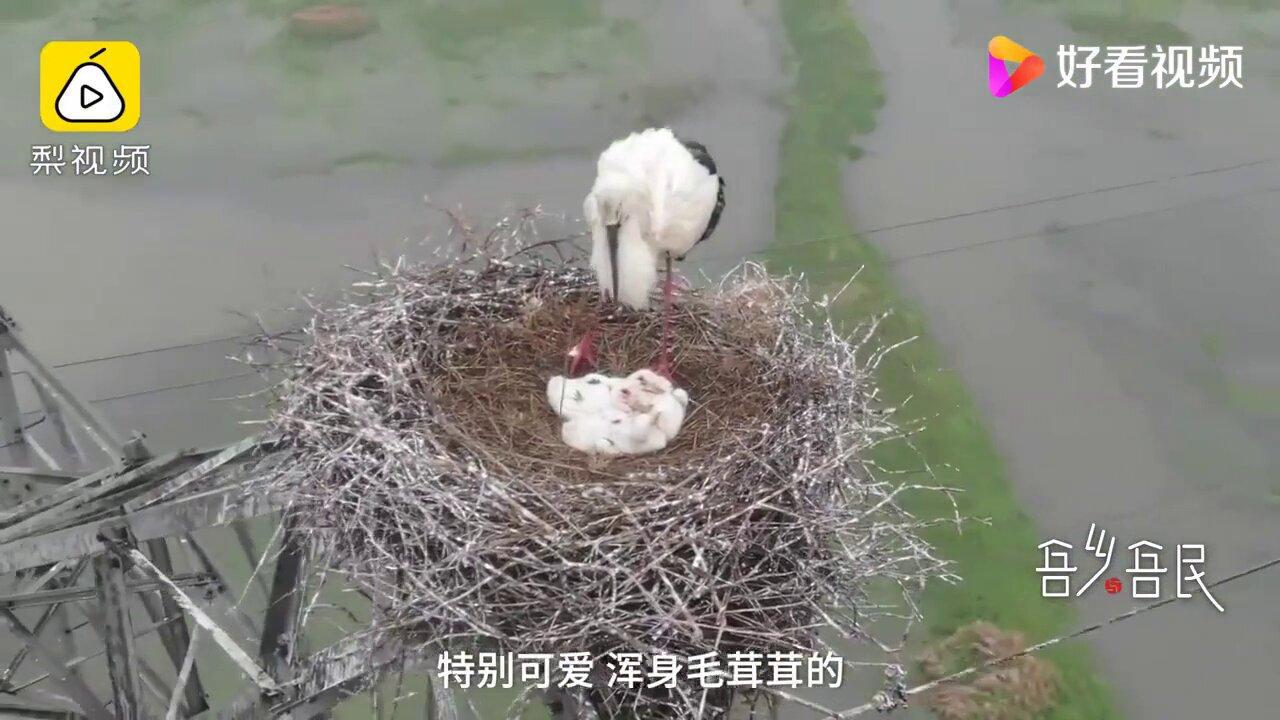

Supplement: Data S1 [file peerj-cs-10-2383-s001.zip › JPEGImages/2BL98HQ4Swm5ZPO0oAGikjbvRlhuFaKzdsUYyECM.jpg]

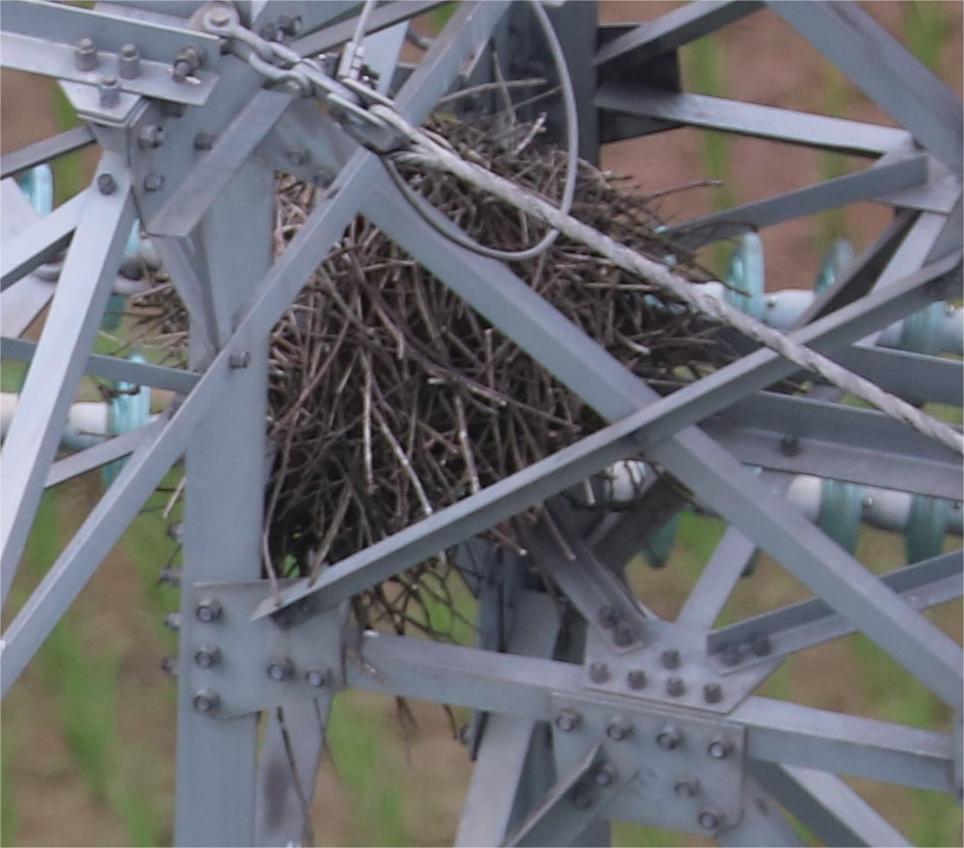

Supplement: Data S1 [file peerj-cs-10-2383-s001.zip › JPEGImages/2Dk9ZHESz8PKMwRcU7prfeFbaOv1gd5VqIuhTYxG.jpg]

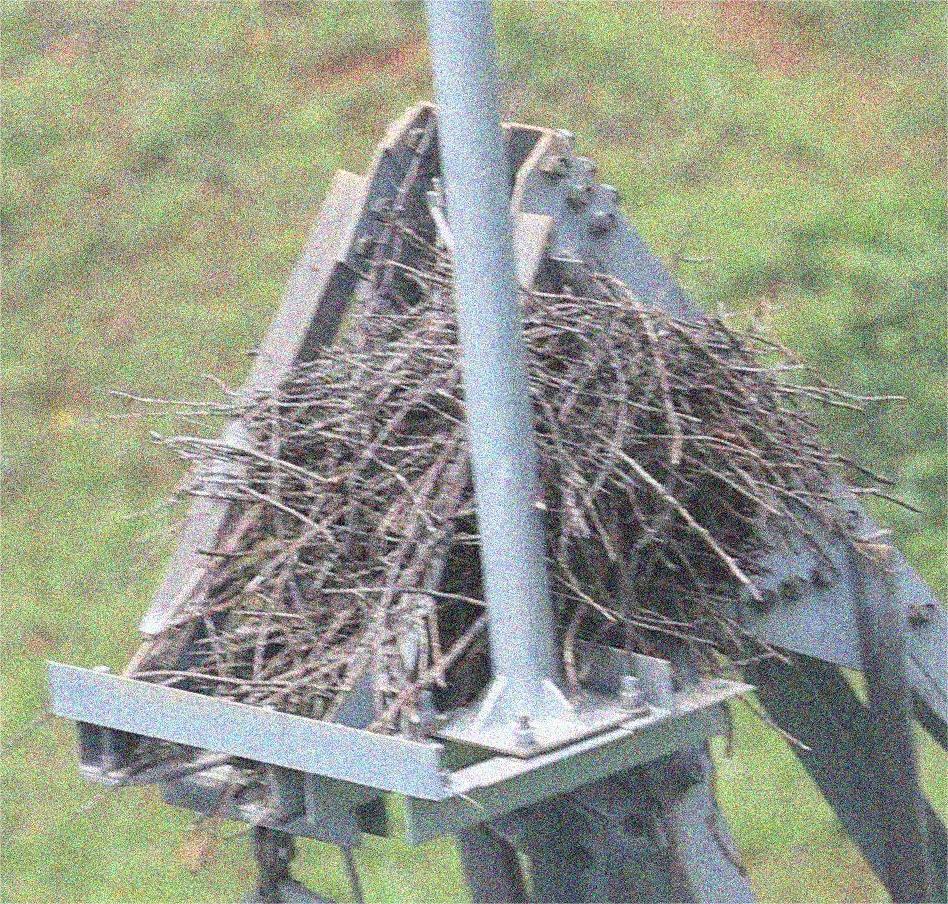

Supplement: Data S1 [file peerj-cs-10-2383-s001.zip › JPEGImages/2HpLZsekJV5TfY1vm0BSN6jRrlMnbOdF79qzXI4W.jpg]

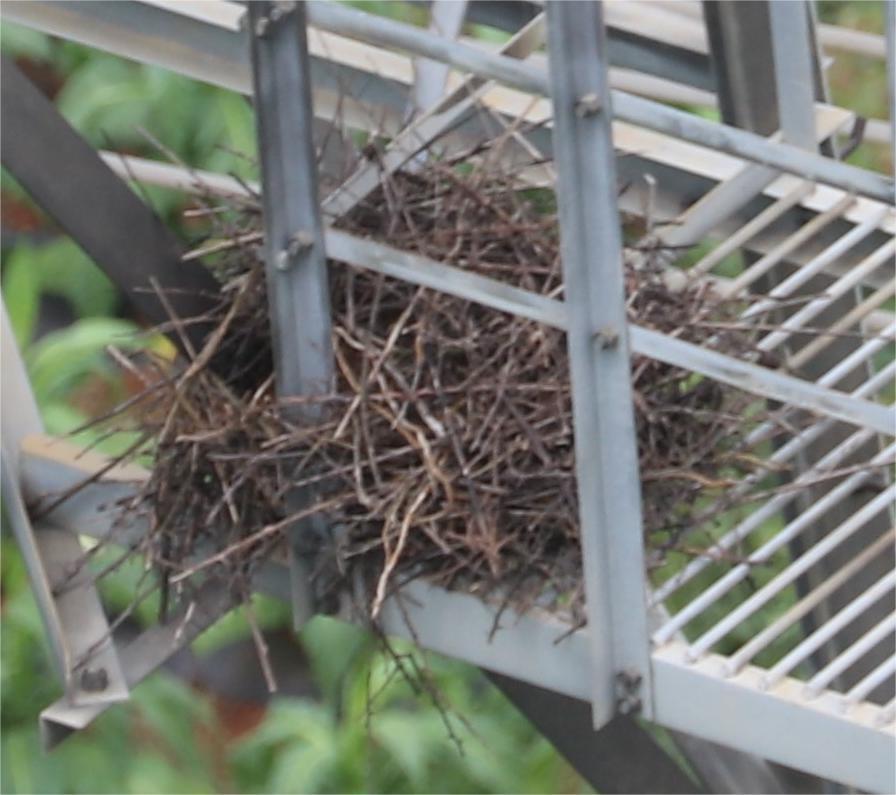

Supplement: Data S1 [file peerj-cs-10-2383-s001.zip › JPEGImages/2QqjEPDpRAkSv7gyLVHBOCibZ3KmU8cTIfx6MwF5.jpg]

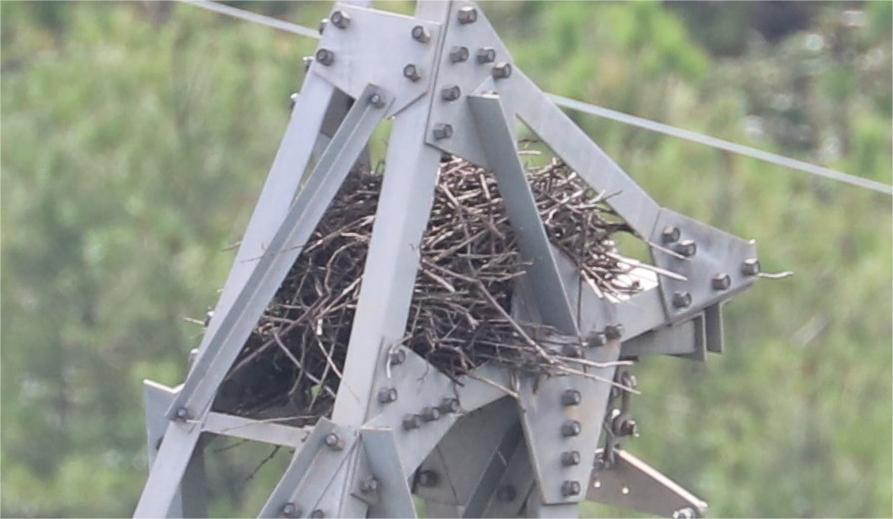

Supplement: Data S1 [file peerj-cs-10-2383-s001.zip › JPEGImages/2YyPdbQqoG1EamCxOD4Xrl5tRcNSUZi6hVpvWMeT.jpg]

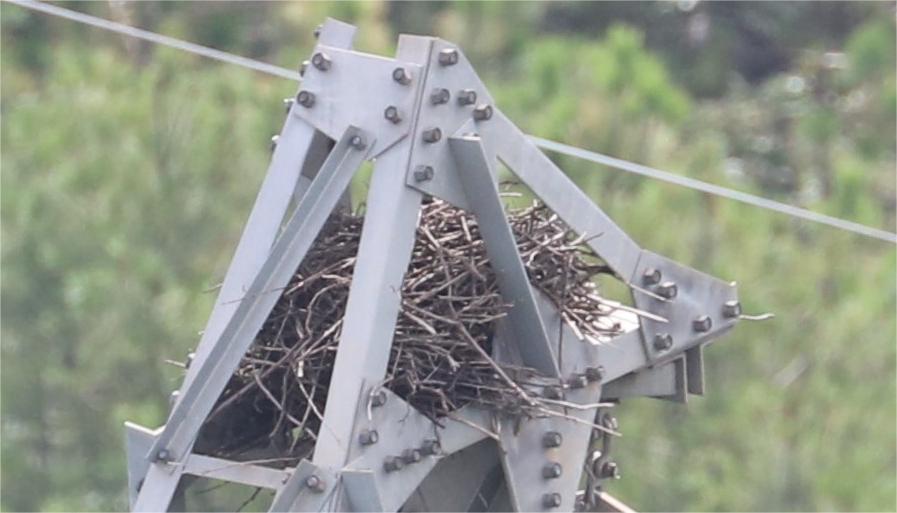

Supplement: Data S1 [file peerj-cs-10-2383-s001.zip › JPEGImages/2b0MAqwYFucigysd5azBnGWPXT4vJHLKC3pU9he1.jpg]

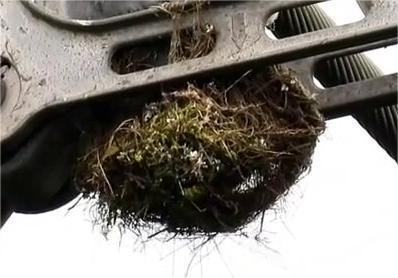

Supplement: Data S1 [file peerj-cs-10-2383-s001.zip › JPEGImages/2d4CByTfL7vHhx3lQXqzg9kr0nY1OpA5GwaIKiEs.jpg]

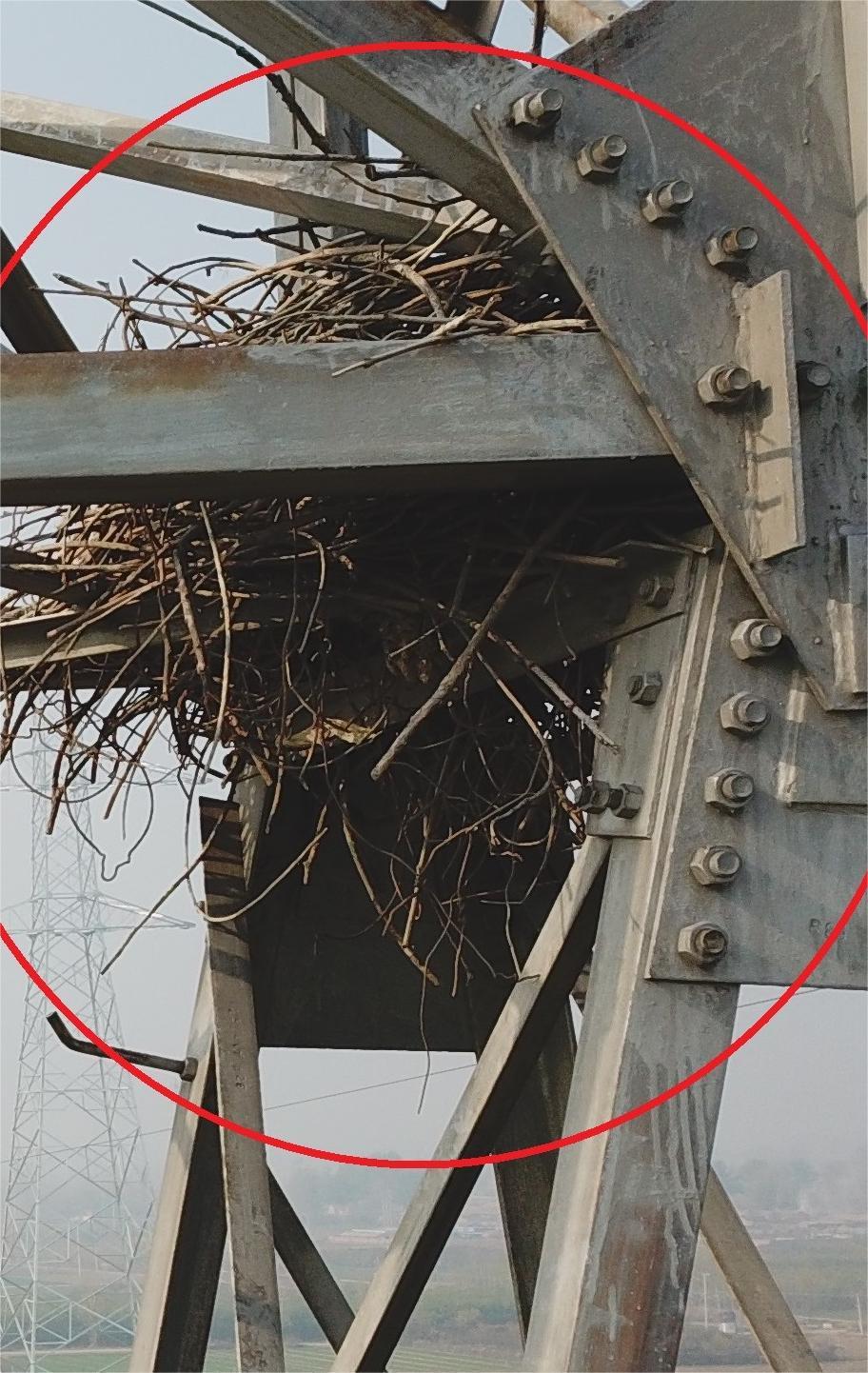

Supplement: Data S1 [file peerj-cs-10-2383-s001.zip › JPEGImages/2eULz1JMgnwoYuX6GpW0fhlDcaT3tPHm97dbE5RV.jpg]

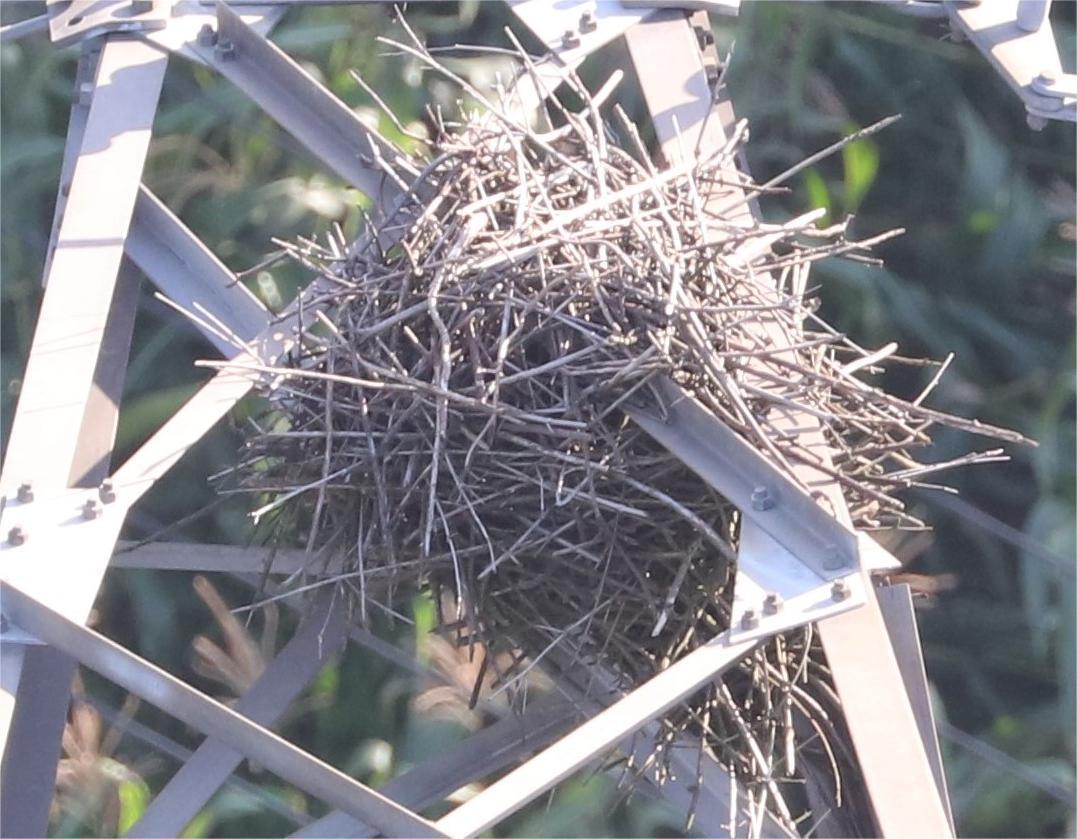

Supplement: Data S1 [file peerj-cs-10-2383-s001.zip › JPEGImages/3ObCXkJpNxWuy5VSKY6RiPdgEIZl27AT9hfeU8Ln.jpg]

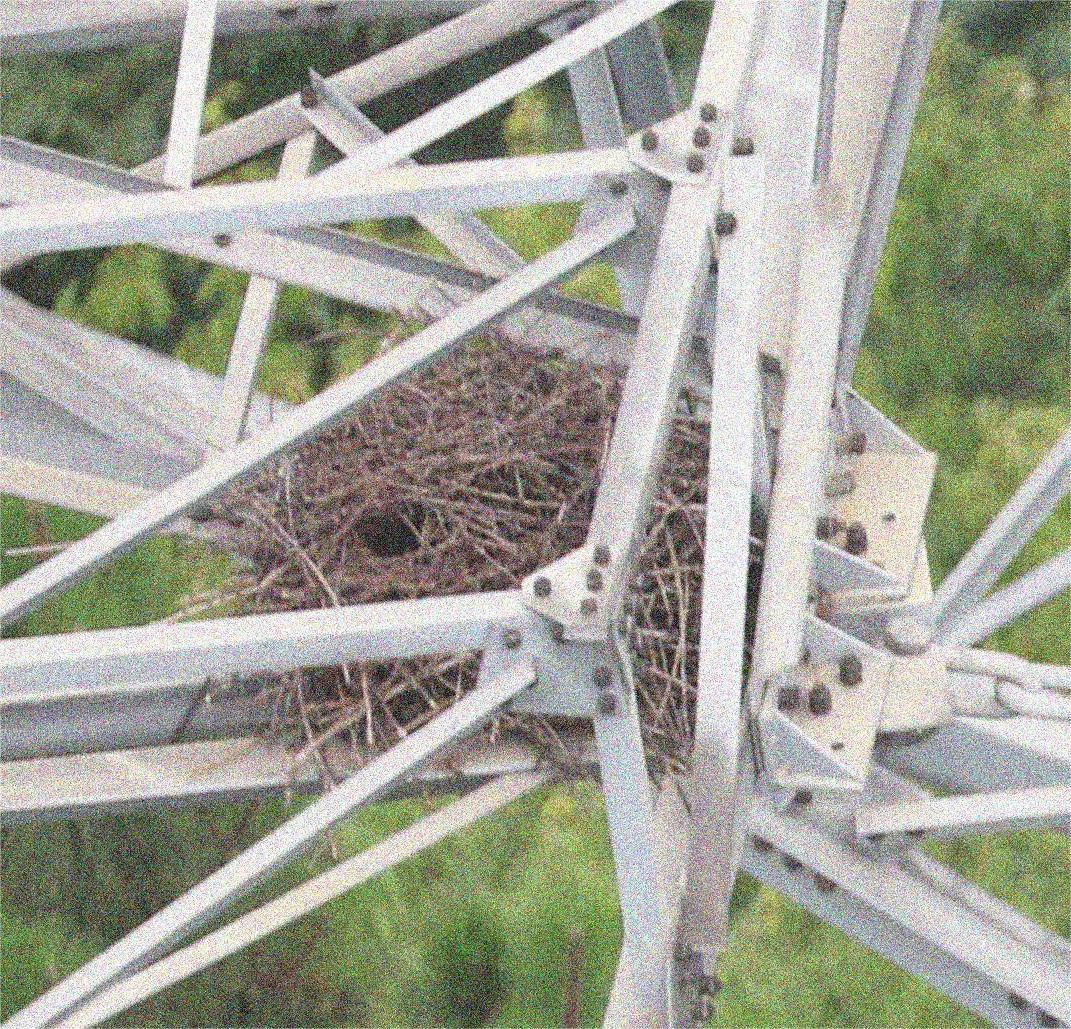

Supplement: Data S1 [file peerj-cs-10-2383-s001.zip › JPEGImages/3dRblyjGn6DXvUZKcoJwOB8NSCa7srfp5mkYHz4I.jpg]

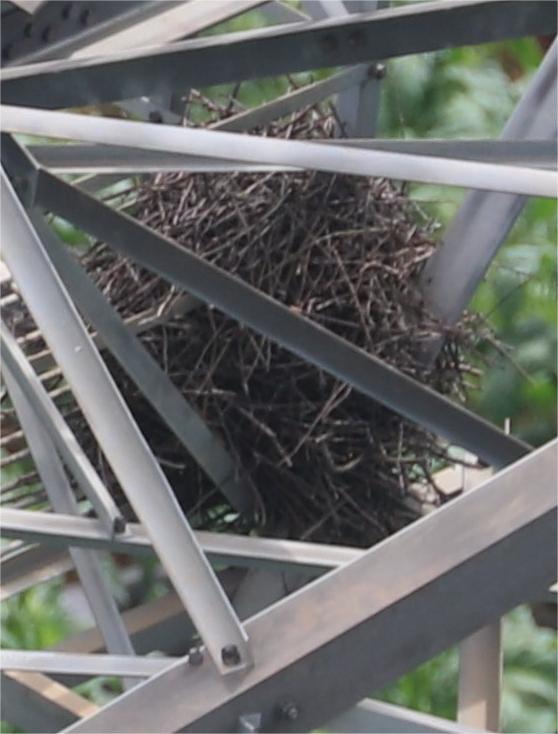

Supplement: Data S1 [file peerj-cs-10-2383-s001.zip › JPEGImages/3mY8G2B15pjfUTkyO0eASlsgFR9iZoQJbILqW7cV.jpg]

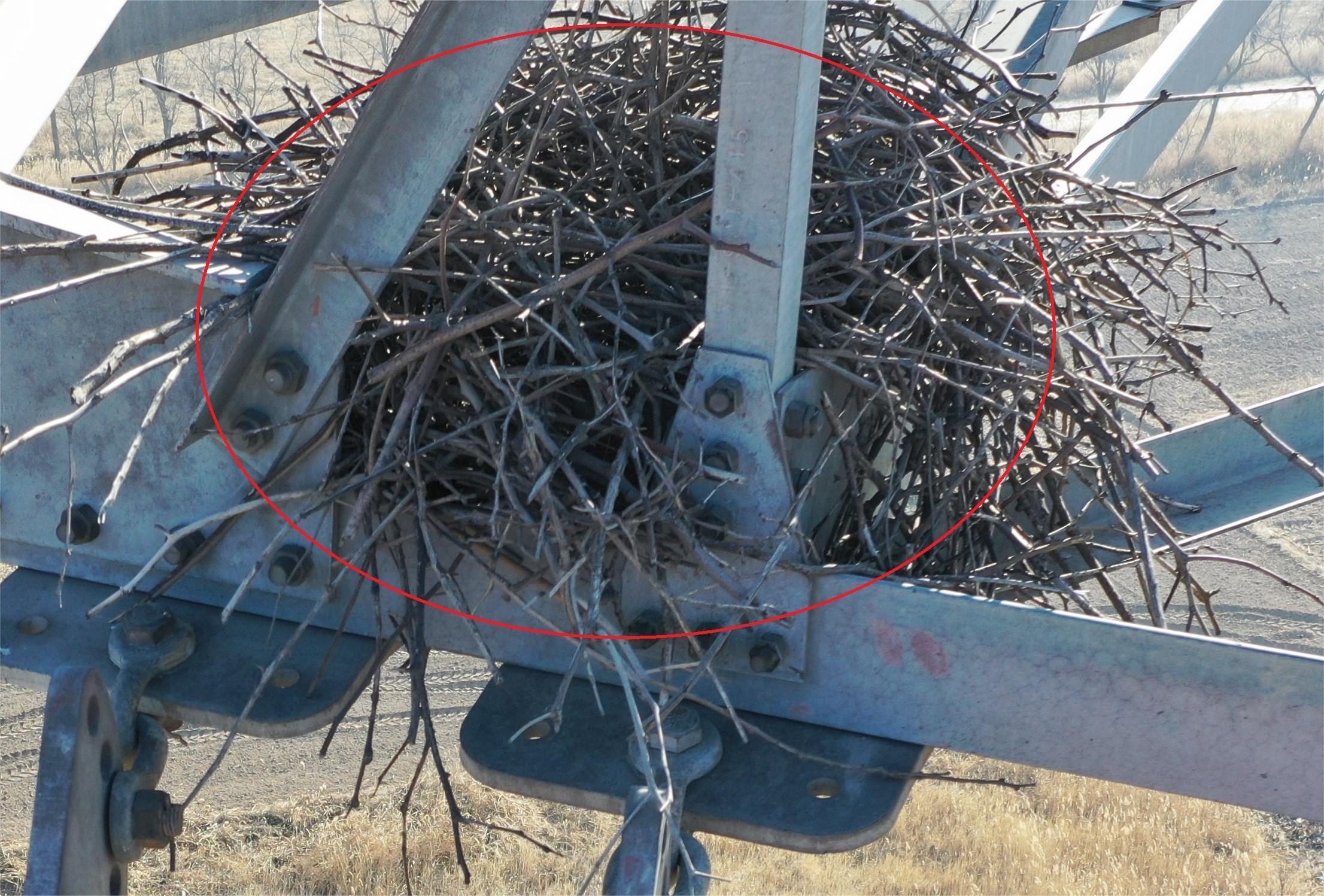

Supplement: Data S1 [file peerj-cs-10-2383-s001.zip › JPEGImages/3tmHuv7J61FTp0VGlEksRfgYhNI29daAw8bqznoy.jpg]

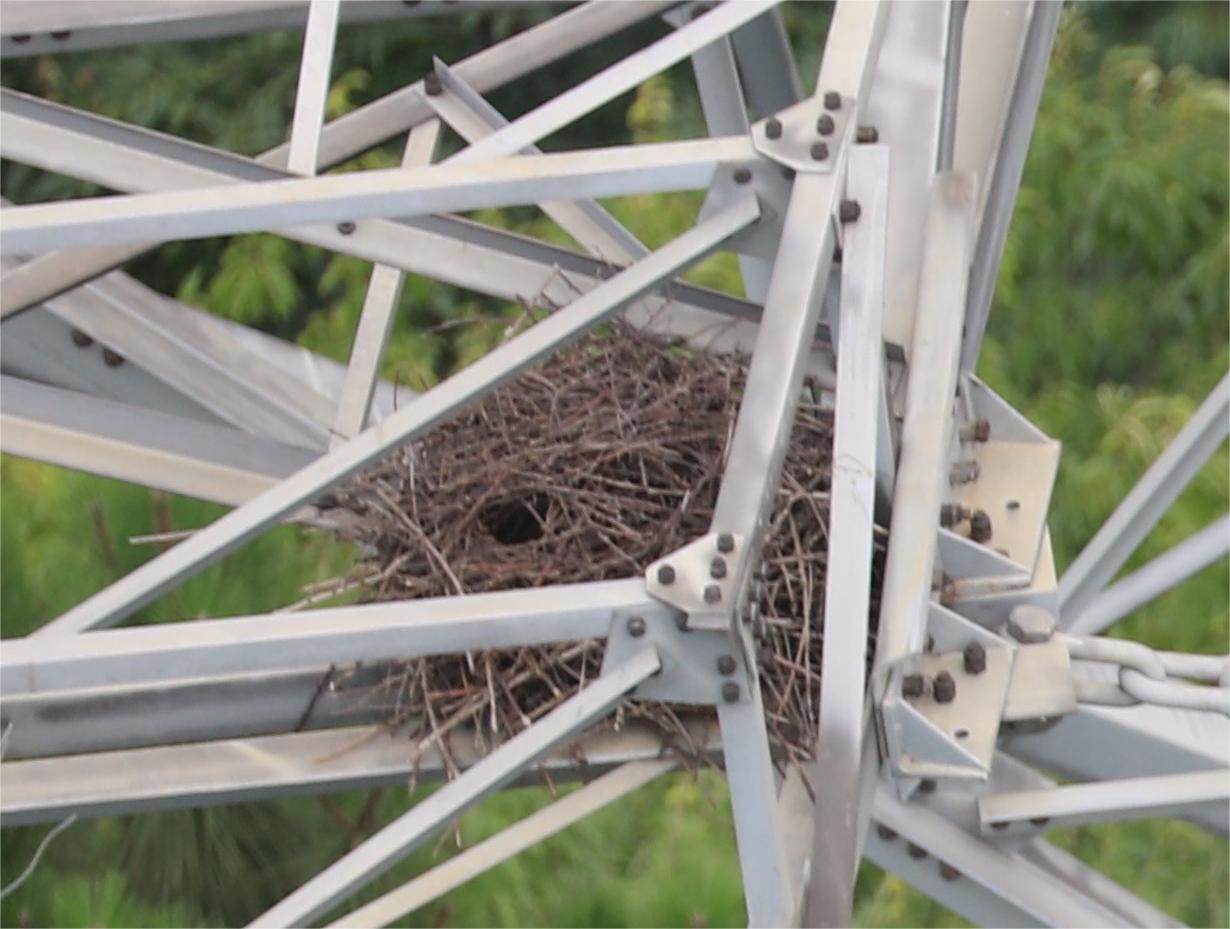

Supplement: Data S1 [file peerj-cs-10-2383-s001.zip › JPEGImages/4K3SElCZy7V9t0zMXnpNoiUBhQTAqa5jwcxvuWIJ.jpg]

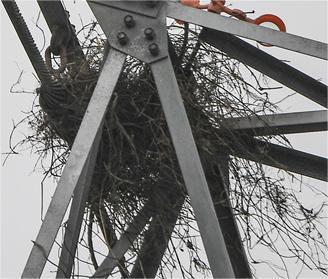

Supplement: Data S1 [file peerj-cs-10-2383-s001.zip › JPEGImages/4XbDnCpGKqNwJfmcQYWRerEx9Fh5ySBI7P8LHOvs.jpg]

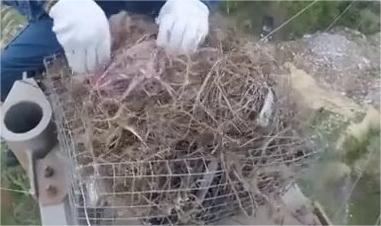

Supplement: Data S1 [file peerj-cs-10-2383-s001.zip › JPEGImages/4qpWNzmak9bRlU2D8wy1KiYPTEQCLgvthVsI0AHj.jpg]

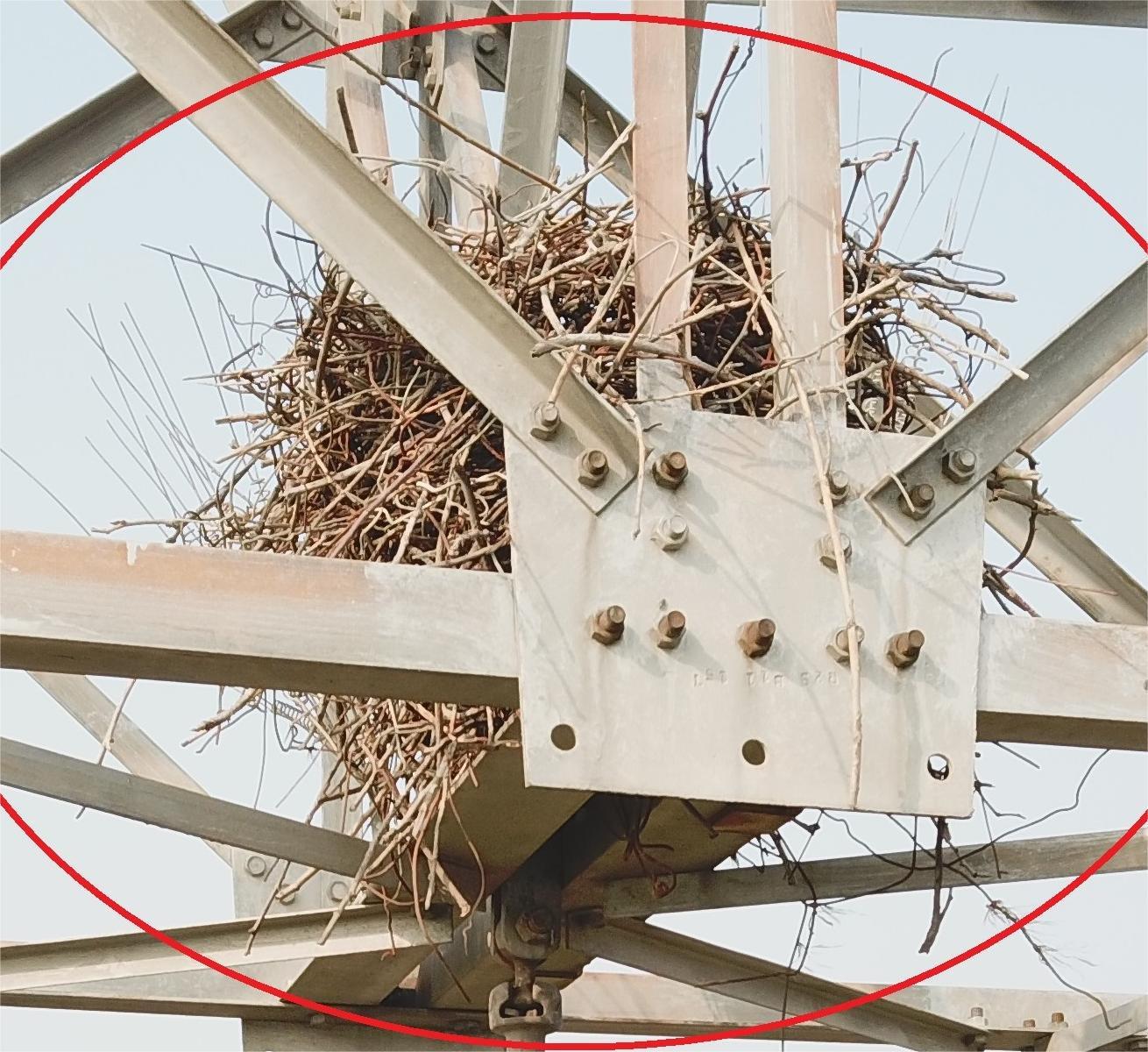

Supplement: Data S1 [file peerj-cs-10-2383-s001.zip › JPEGImages/4tq9dOEaJYfVx2nMQvz6LFcBjrUis1C8HwDoXR7b.jpg]

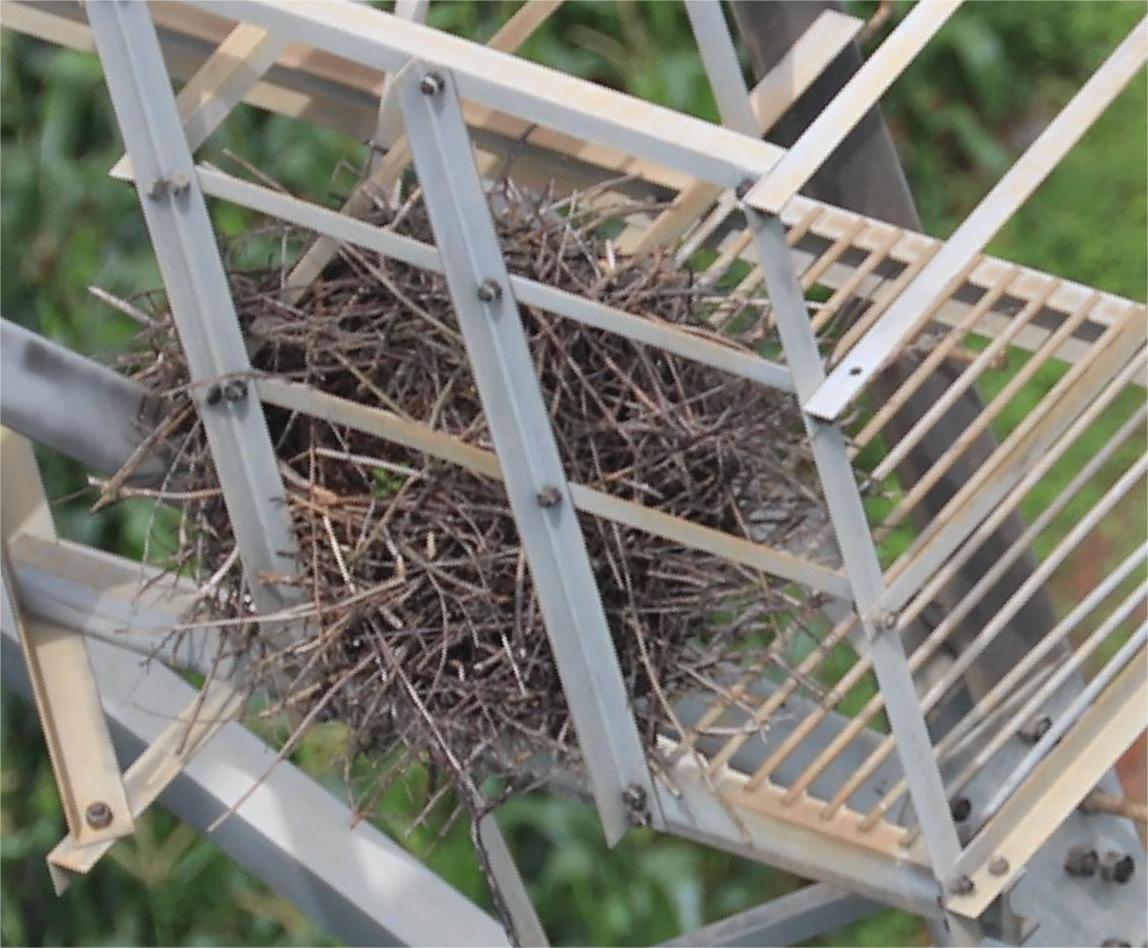

Supplement: Data S1 [file peerj-cs-10-2383-s001.zip › JPEGImages/5GTutFxjeVEMlwfo26SXdc0sO3JgpIRyWUqPKv1a.jpg]

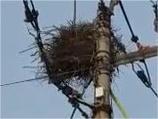

Supplement: Data S1 [file peerj-cs-10-2383-s001.zip › JPEGImages/5RPu1KDyQGVCe92sgndvXHmxj8p4BZtkFhzaMr63.jpg]

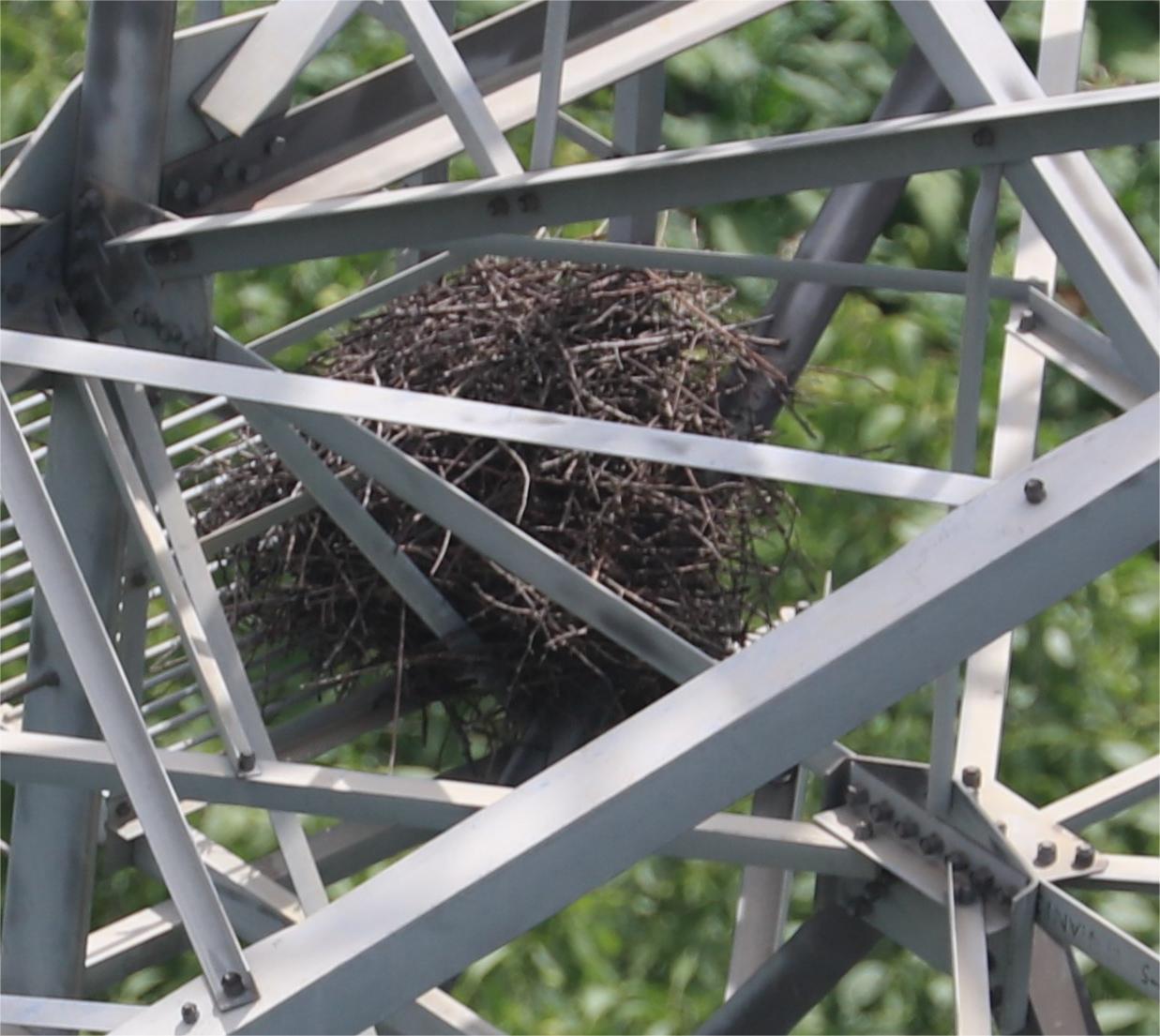

Supplement: Data S1 [file peerj-cs-10-2383-s001.zip › JPEGImages/5dO1jFgzAMp6BEWPTYRb84aSUVouDNn3vGmwClkh.jpg]

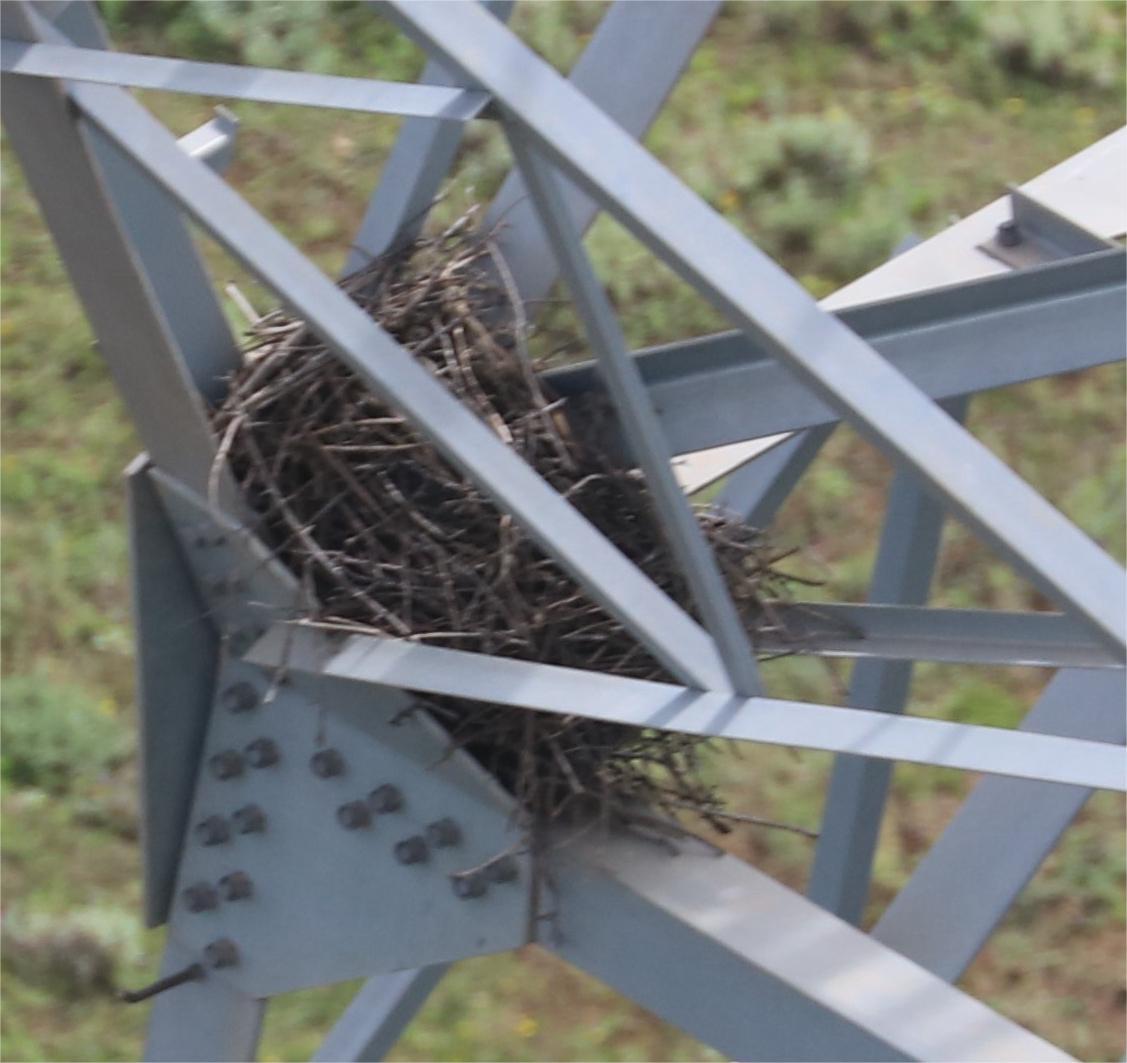

Supplement: Data S1 [file peerj-cs-10-2383-s001.zip › JPEGImages/5pWsMwkC60K8Ff2xhP9iBcUnbjyudLq4gQG1AY7X.jpg]

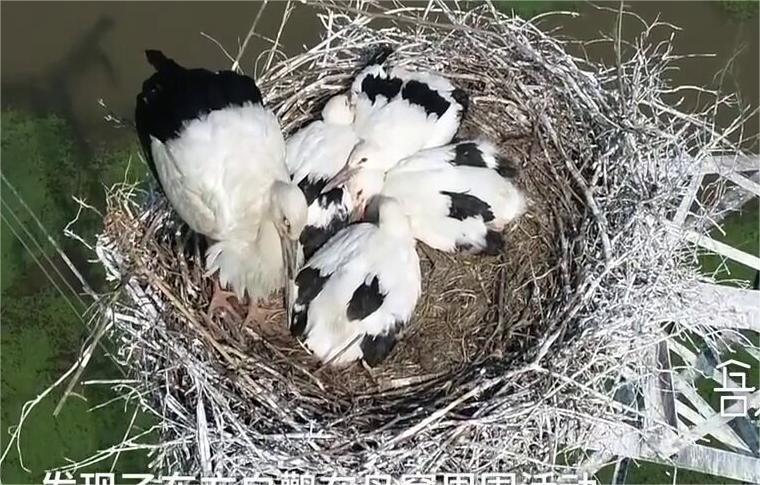

Supplement: Data S1 [file peerj-cs-10-2383-s001.zip › JPEGImages/6NtYzPFoQm2GvZf1eXpr4q5wSi9lLBVHaECsbnW8.jpg]

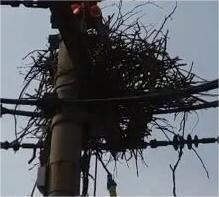

Supplement: Data S1 [file peerj-cs-10-2383-s001.zip › JPEGImages/6WtNQ5L8BsdFqeaSAwlhRJoYMyZk4VOn7D3Pjf1I.jpg]

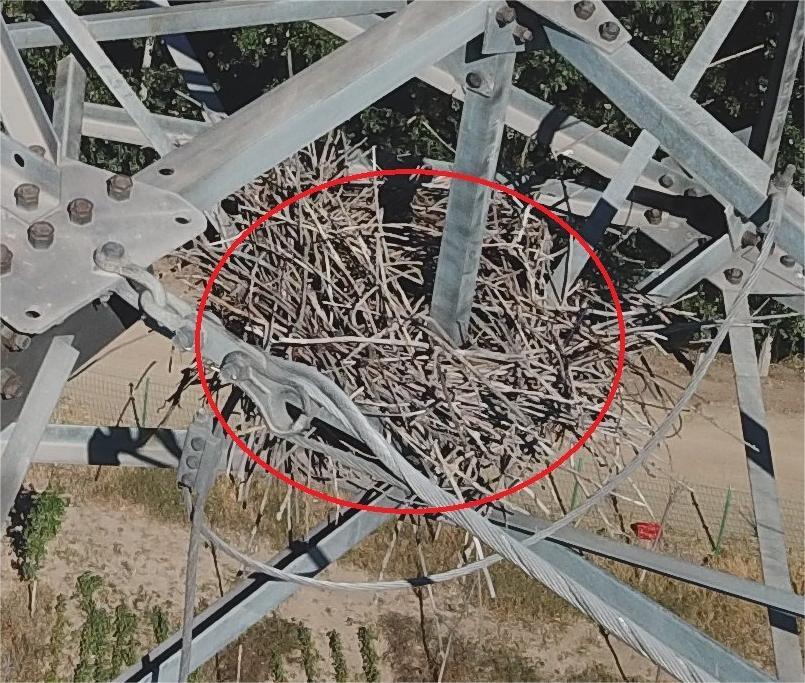

Supplement: Data S1 [file peerj-cs-10-2383-s001.zip › JPEGImages/6pSfn1gzPLUd7iFMZCsl2mTBvRY5bIuXhOQJjatq.jpg]

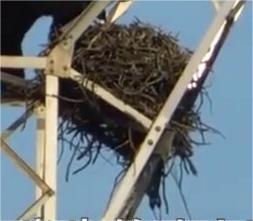

Supplement: Data S1 [file peerj-cs-10-2383-s001.zip › JPEGImages/6sOfIj4AFB9XvNtVo3bxGM5r0RWHmkS8Qa7Lznwc.jpg]

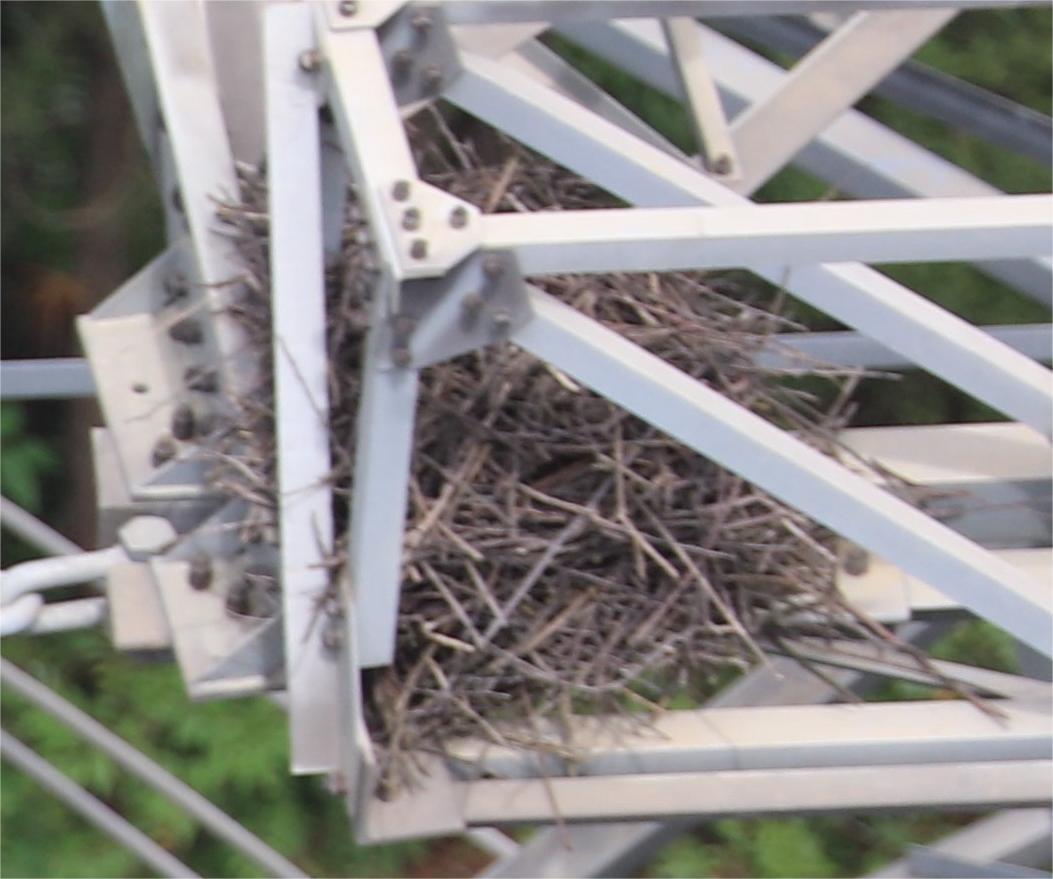

Supplement: Data S1 [file peerj-cs-10-2383-s001.zip › JPEGImages/6u0vaGzrqZ3eT8SxKtlsYP7EnBXQkLFVpJMW21cw.jpg]

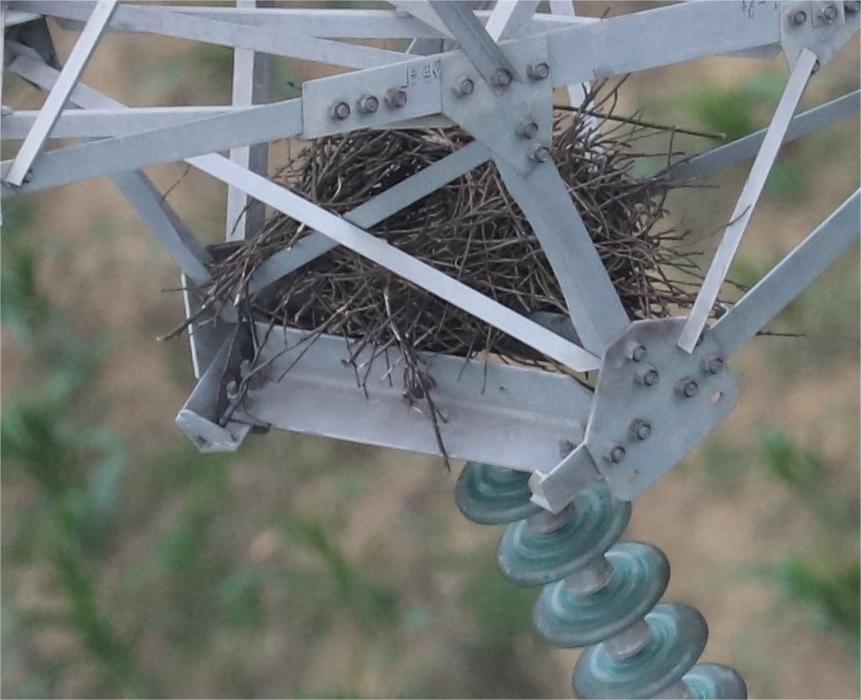

Supplement: Data S1 [file peerj-cs-10-2383-s001.zip › JPEGImages/7G8VTmUz4nIqb3sfvri5kHAKYEJXdO6xNMac0j1Q.jpg]

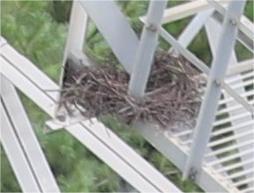

Supplement: Data S1 [file peerj-cs-10-2383-s001.zip › JPEGImages/7GlCpyk4AdsTHet0JKrX8bOjRiva61uDhYgNqWno.jpg]

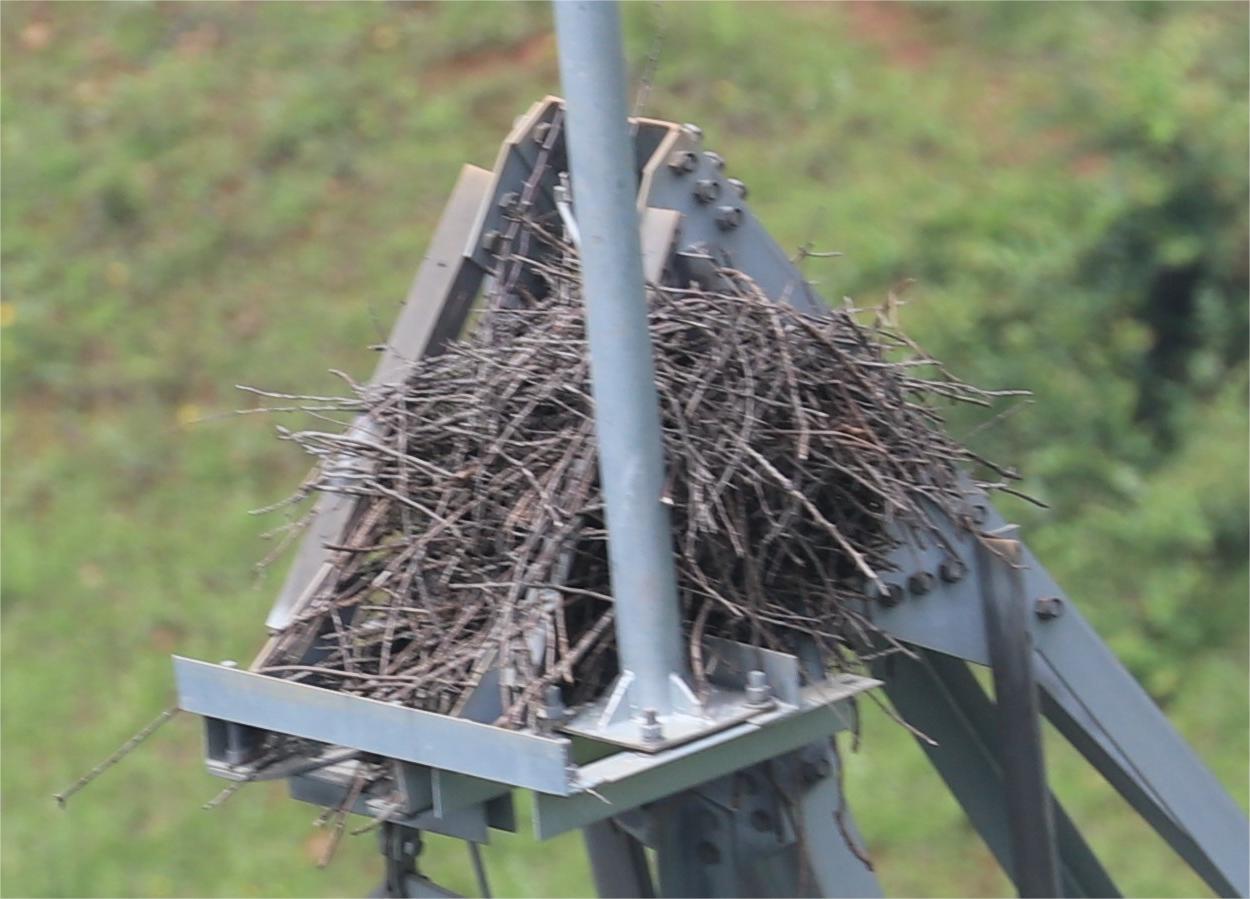

Supplement: Data S1 [file peerj-cs-10-2383-s001.zip › JPEGImages/7bRNBKw9LQ3Cq5vUPgxy8oc1ZTJSMp6fYIWadXsh.jpg]

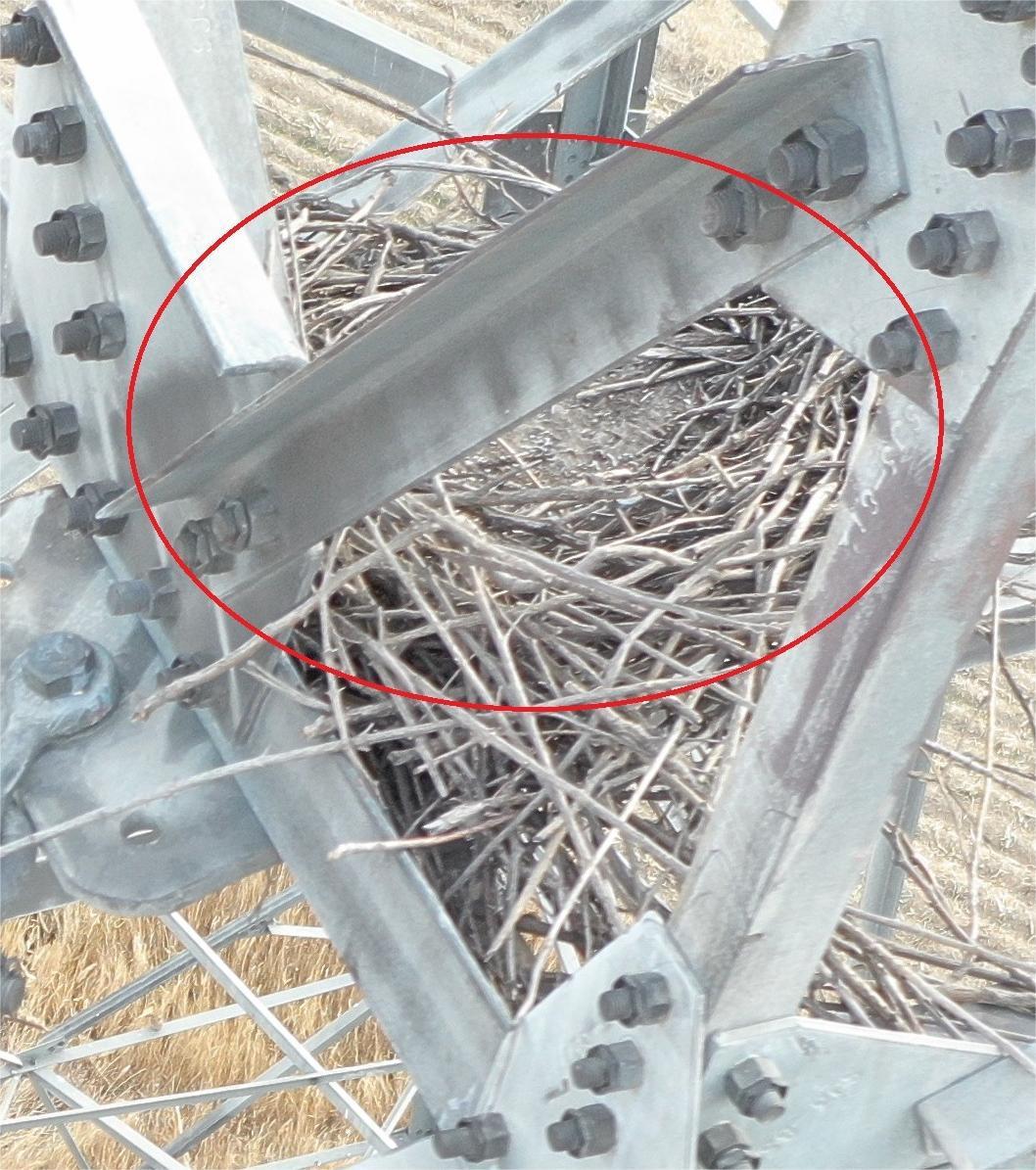

Supplement: Data S1 [file peerj-cs-10-2383-s001.zip › JPEGImages/7notshyVpuq0jMxbRJBWKQi3AS1XdPL2re9zfm8l.jpg]

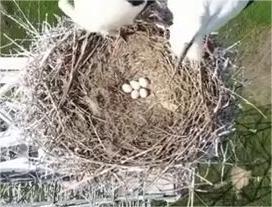

Supplement: Data S1 [file peerj-cs-10-2383-s001.zip › JPEGImages/7qloOEU51QGvZjnJwg4bK2W09zh6VsfadiLSRTDH.jpg]

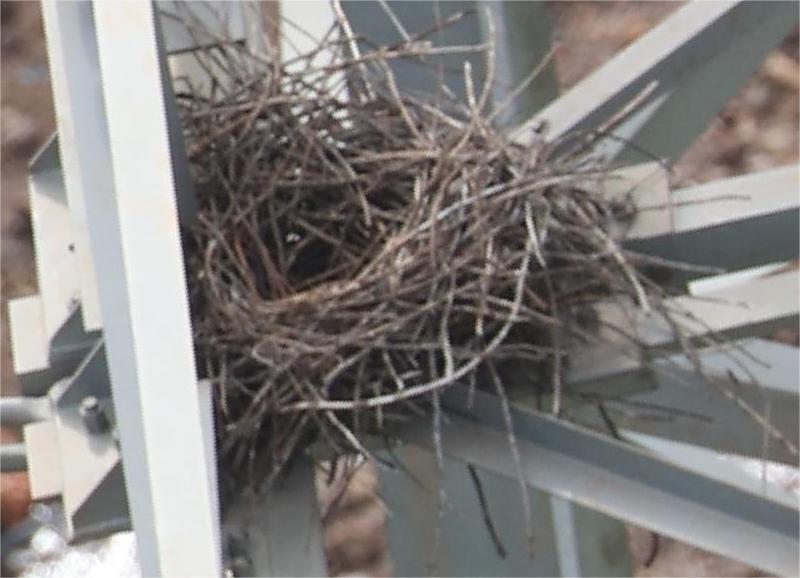

Supplement: Data S1 [file peerj-cs-10-2383-s001.zip › JPEGImages/8VEZiuQCopAfDtn60hUaNxSdkOWIgrYc9bR7ywMe.jpg]

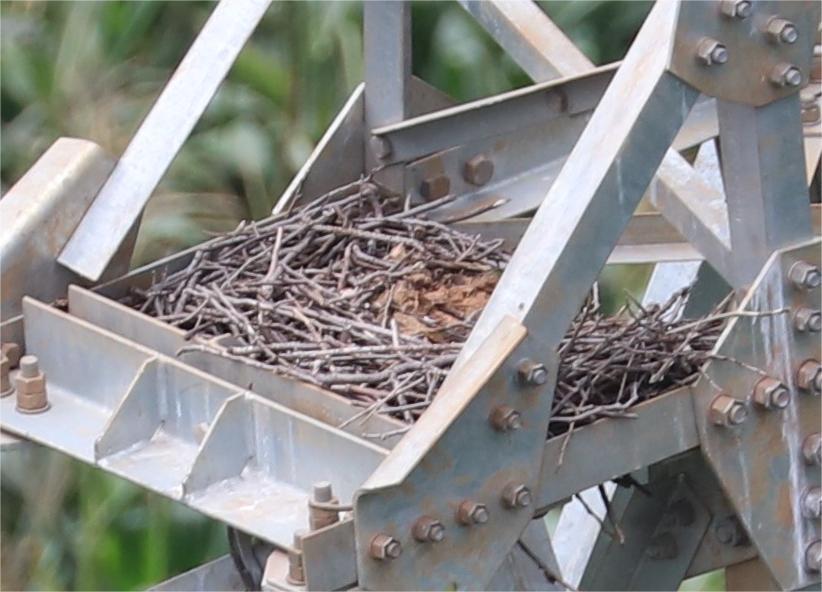

Supplement: Data S1 [file peerj-cs-10-2383-s001.zip › JPEGImages/8Wj0G15Y4rowKXBVDAyHb3u96qOtFimazsIcUNQl.jpg]

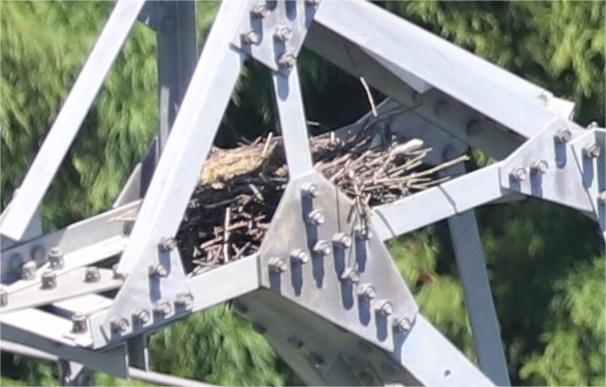

Supplement: Data S1 [file peerj-cs-10-2383-s001.zip › JPEGImages/8iXzFatpoAY6bjP29Jvdr3hkUEB14V5KqQCWLDxZ.jpg]

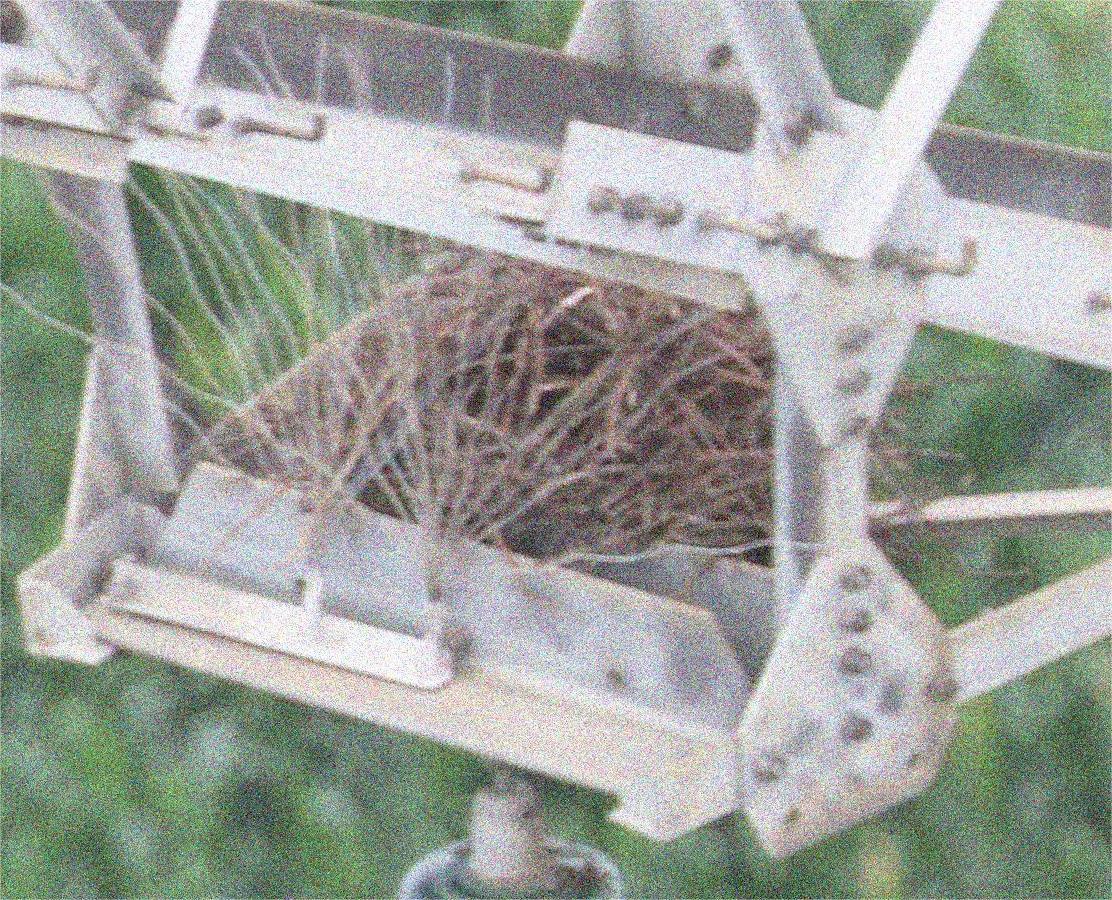

Supplement: Data S1 [file peerj-cs-10-2383-s001.zip › JPEGImages/8yHkqmtcLOXuYj7QNnfgVD9Z0Wdw4ApbR5hCP6l2.jpg]

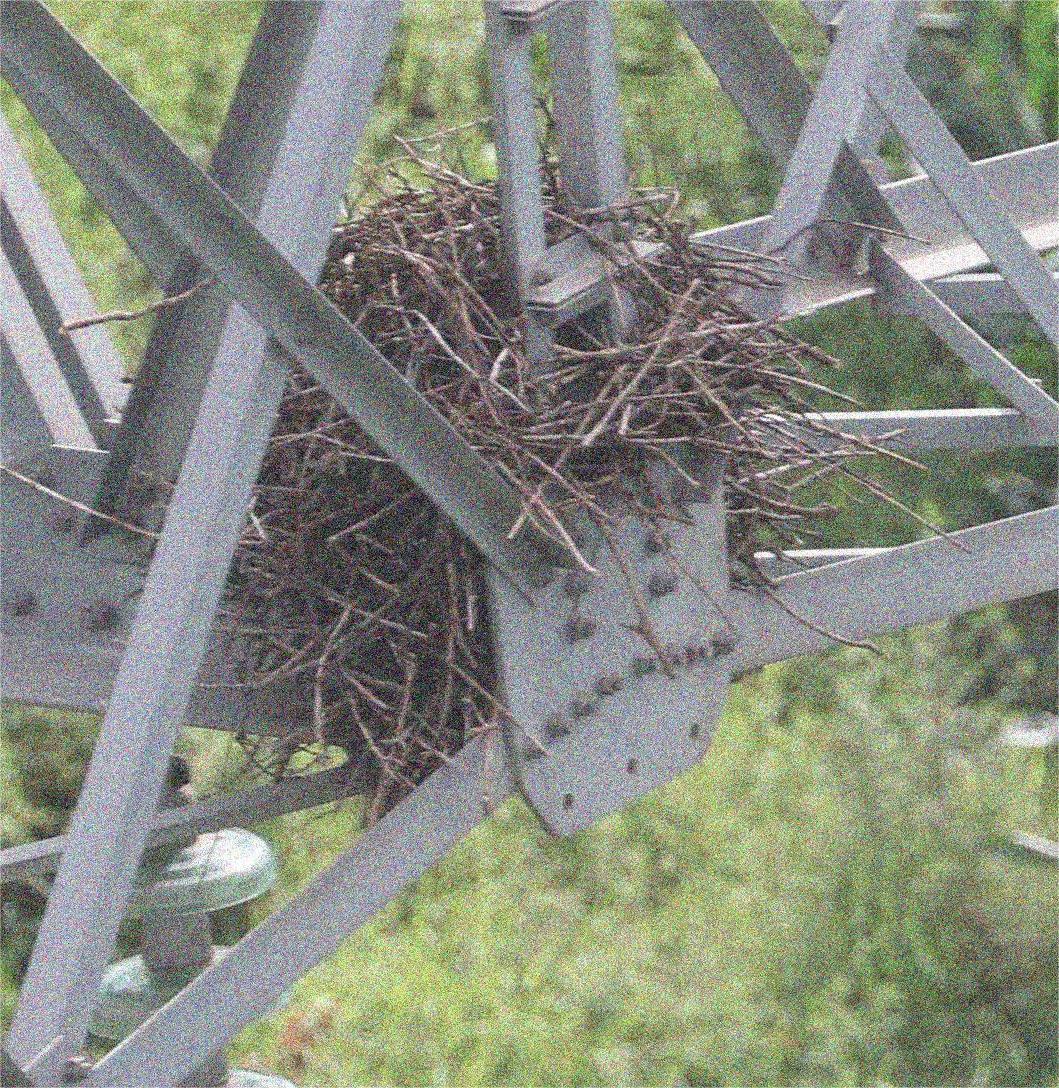

Supplement: Data S1 [file peerj-cs-10-2383-s001.zip › JPEGImages/8ysGSZYPCdpo0rvJRQ5tTjKHNhIbVz6MU4amDLni.jpg]

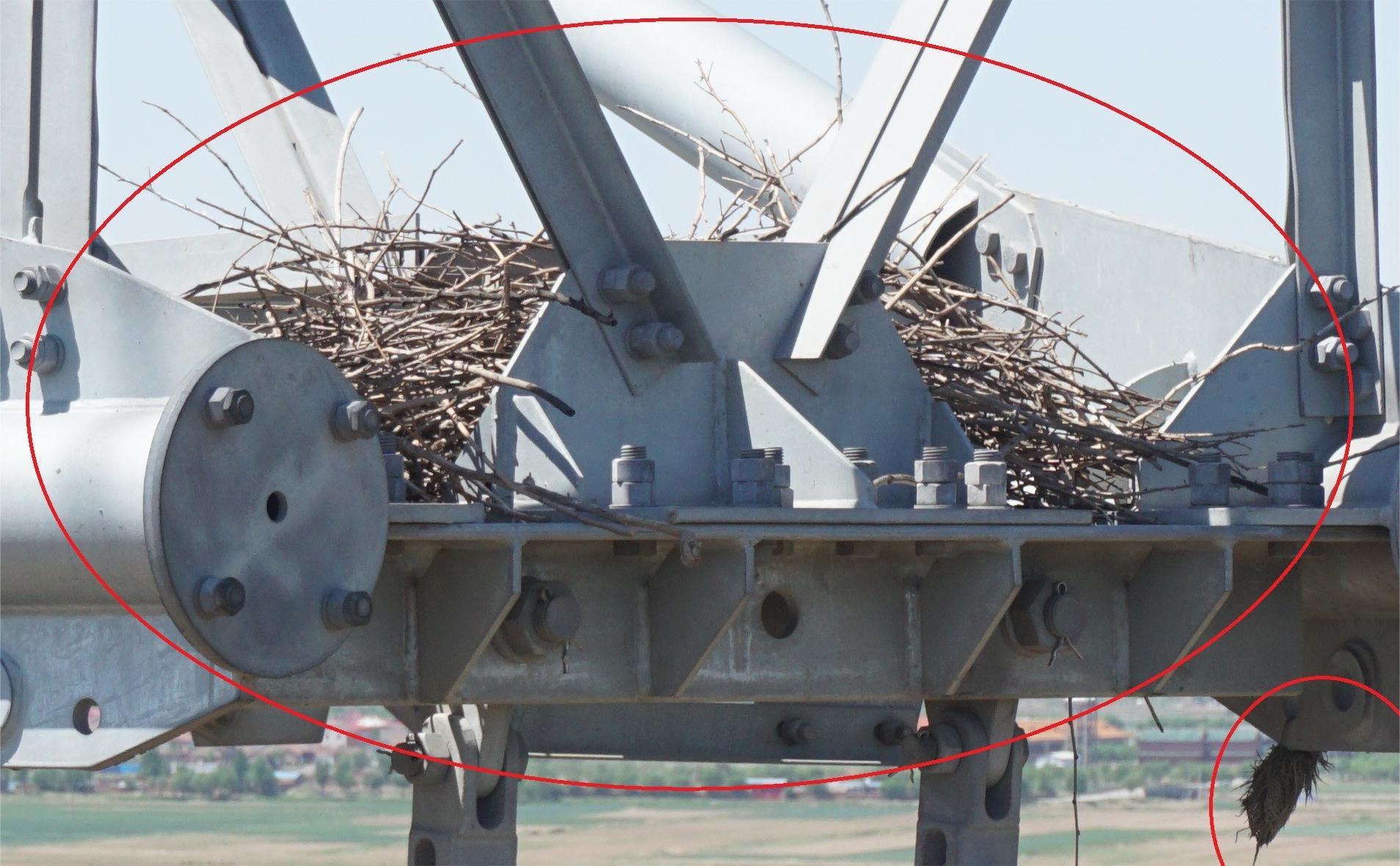

Supplement: Data S1 [file peerj-cs-10-2383-s001.zip › JPEGImages/8zAuX3vBrkJdFlZ0YqnHgSsD52WhGIU6CRaf9bOj.jpg]

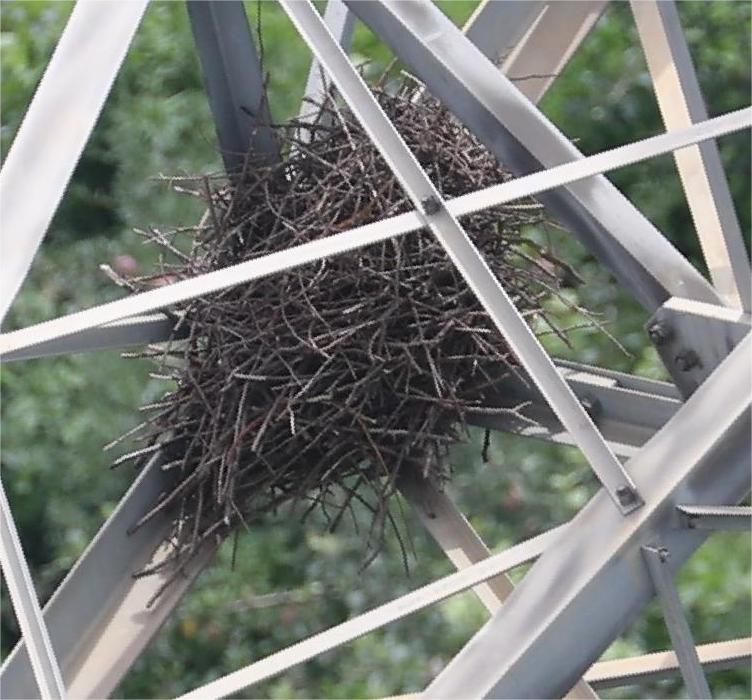

Supplement: Data S1 [file peerj-cs-10-2383-s001.zip › JPEGImages/9ty7MLBGec6NUlrsPZKSj1dmo8q5bWkDCR4Tgf3F.jpg]

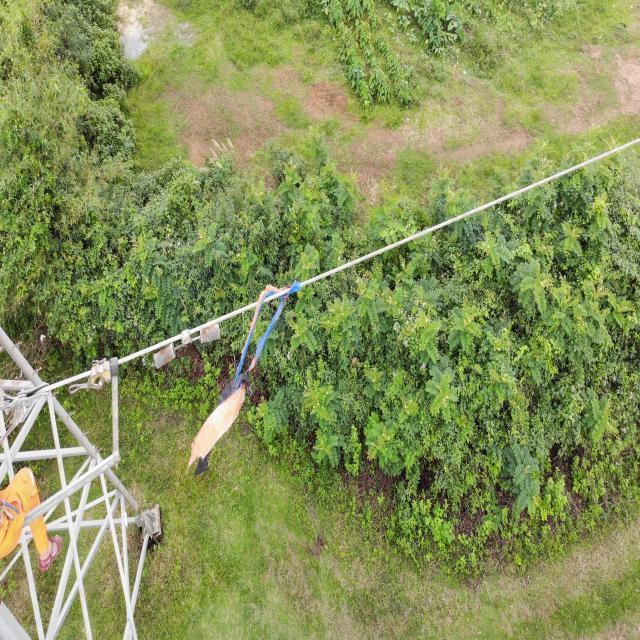

Supplement: Data S1 [file peerj-cs-10-2383-s001.zip › JPEGImages/DJI_0001_JPG.jpg]

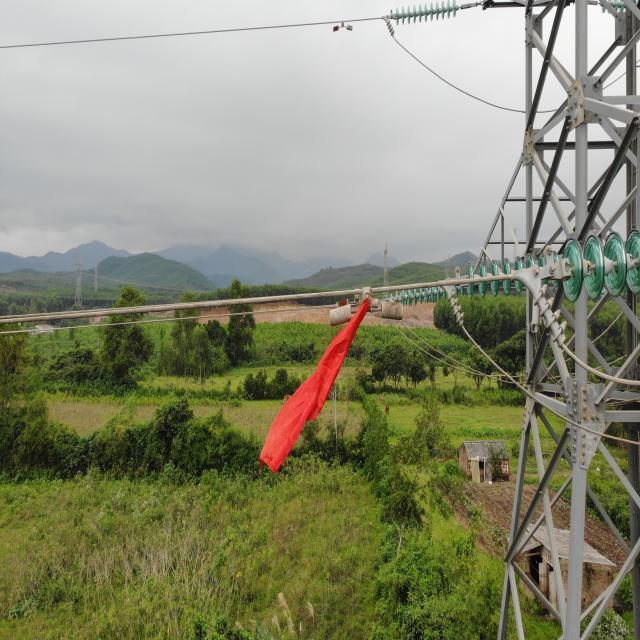

Supplement: Data S1 [file peerj-cs-10-2383-s001.zip › JPEGImages/DJI_0005_JPG.jpg]

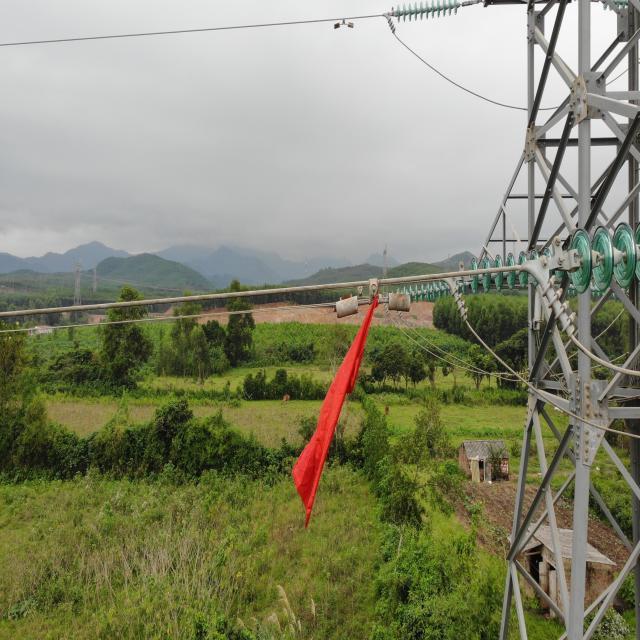

Supplement: Data S1 [file peerj-cs-10-2383-s001.zip › JPEGImages/DJI_0007_JPG.jpg]

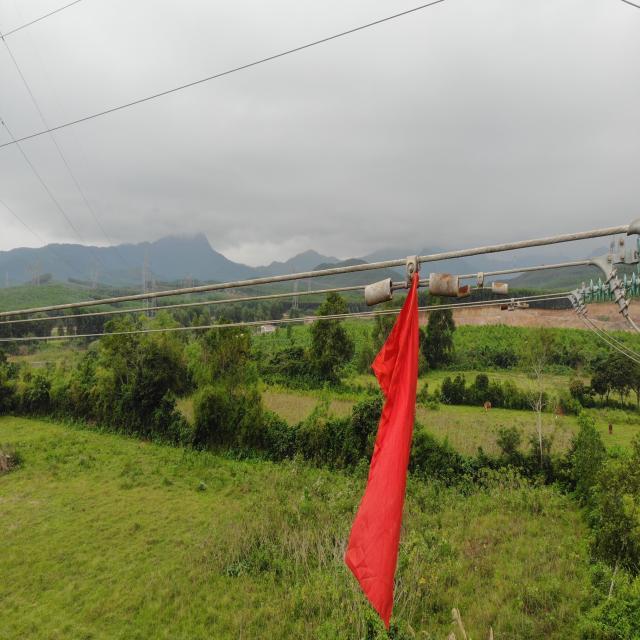

Supplement: Data S1 [file peerj-cs-10-2383-s001.zip › JPEGImages/DJI_0009_JPG.jpg]

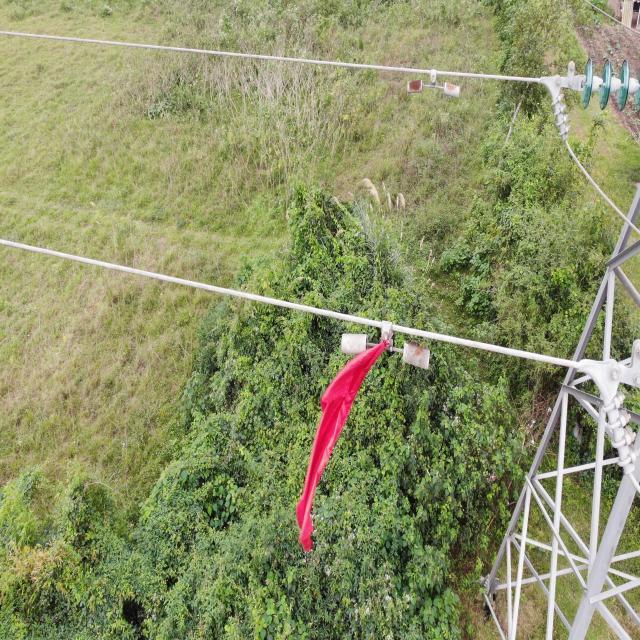

Supplement: Data S1 [file peerj-cs-10-2383-s001.zip › JPEGImages/DJI_0011_JPG.jpg]

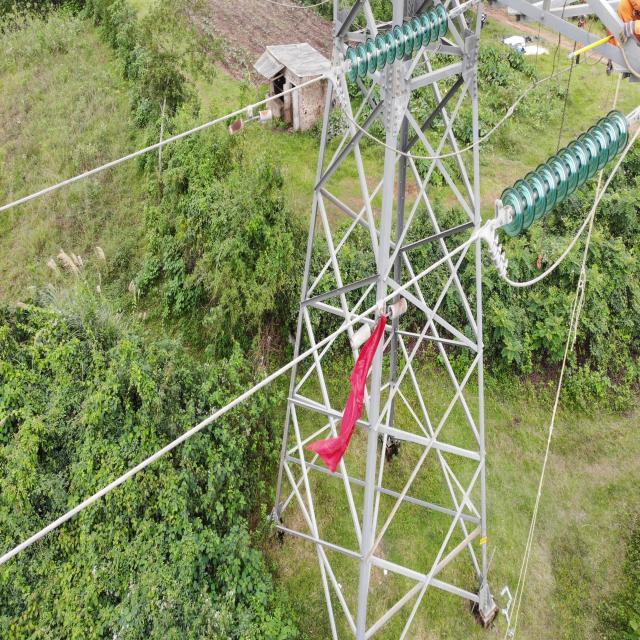

Supplement: Data S1 [file peerj-cs-10-2383-s001.zip › JPEGImages/DJI_0013_JPG.jpg]

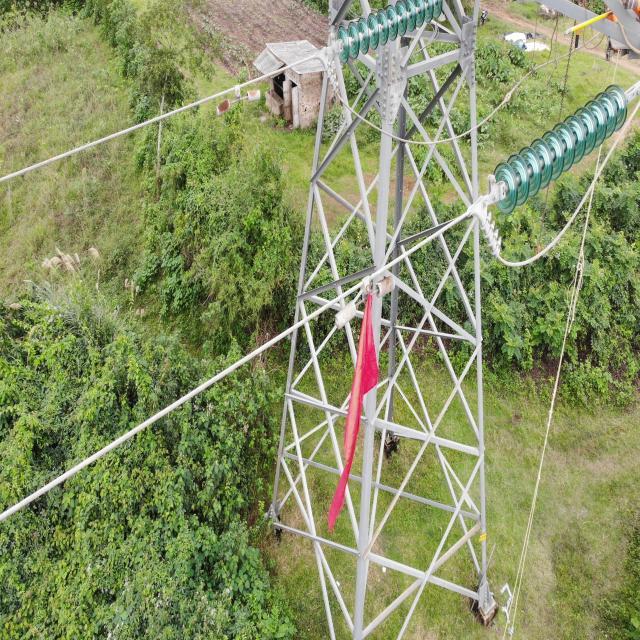

Supplement: Data S1 [file peerj-cs-10-2383-s001.zip › JPEGImages/DJI_0015_JPG.jpg]
